# Heavy-Atom Free *spiro* Organoboron Complexes As Triplet Excited States Photosensitizers for Singlet Oxygen Activation

**DOI:** 10.1021/acs.joc.1c01254

**Published:** 2021-09-01

**Authors:** Paulina H. Marek-Urban, Mateusz Urban, Magdalena Wiklińska, Klaudia Paplińska, Krzysztof Woźniak, Agata Blacha-Grzechnik, Krzysztof Durka

**Affiliations:** † Warsaw University of Technology, Faculty of Chemistry, Noakowskiego 3, 00-664 Warsaw, Poland; ‡ University of Warsaw, Faculty of Chemistry, Pasteura 1, 02-093 Warsaw, Poland; § Faculty of Chemistry, Silesian University of Technology, Strzody 9, 44-100 Gliwice, Poland

## Abstract

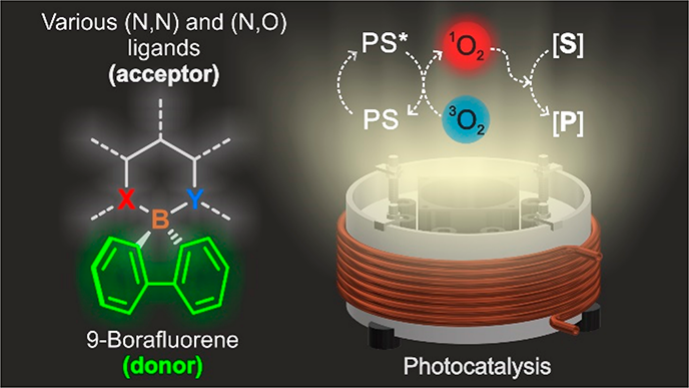

Herein, we present a new strategy for the development of efficient heavy-atom free singlet oxygen photosensitizers based on rigid borafluorene scaffolds. Physicochemical properties of borafluorene complexes can be easily tuned through the choice of ligand, thus allowing exploration of numerous organoboron structures as potent ^1^O_2_ sensitizers. The singlet oxygen generation quantum yields of studied complexes vary in the range of 0.55–0.78. Theoretical calculations reveal that the introduction of the borafluorene moiety is crucial for the stabilization of a singlet charge transfer state, while intersystem crossing to a local triplet state is facilitated by orthogonal donor–acceptor molecular architecture. Our study shows that quantitative oxidation of selected organic substrates can be achieved in 20–120 min of irradiation with only 0.05 mol % loading of a photocatalyst.

## Introduction

Singlet oxygen plays an essential role in a wide range of applications including photocatalysis,^1−3^ water and air purification,^4,5^ photodynamic therapy (PDT),^6−8^*in vivo* oxygen sensing,^9^ and bioimaging.^10^ Photosensitization of molecular oxygen involves light-excitation of the photoactive substance followed by its interconversion to a triplet state and further energy transfer to triplet oxygen (^3^O_2_) generating highly reactive oxygen species such as singlet oxygen (^1^O_2_) or radical anion (O_2_^•–^). To achieve high photosensitizing efficiency, the target triplet photosensitizer (PS) should be characterized by high absorption in the visible or NIR region, high triplet state interconversion probability, low rates of phosphorescence, and high photostability. Although certain groups of compounds such as porphyrins, phthalocyanines,^11−13^ fullerene-based conjugates,^5,14,15^ and heavy-metal atom complexes^16^ fulfill the aforementioned criteria, they may not be attractive for wider application due to the problems associated with their synthesis, processability, environmental hazard, and high cost of production, especially when industrial scales are taken into consideration. For these reasons, small-molecule, heavy atom-free organic photosensitizers constitute particularly interesting alternatives.

**Chart 1:**
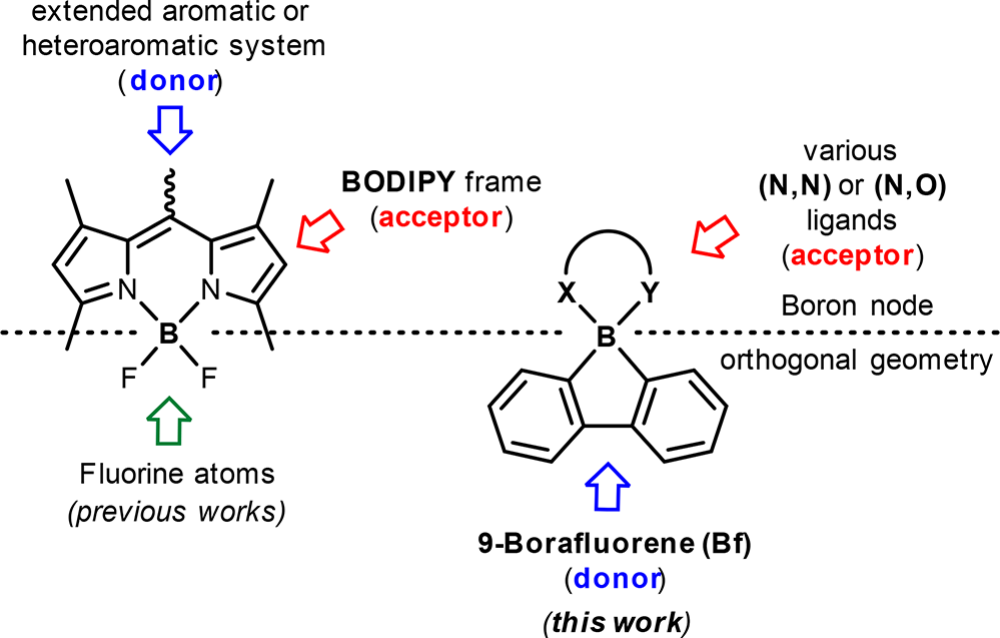
Compact Donor–Acceptor Organoboron Photosensitizers

Due to their controlled structure–property tunability, modular structure, and feasible synthesis, organoboron complexes seem to be ideal candidates for application as singlet oxygen photosensitizers, yet most of them de-excite via fluorescence emission.^17,18^ Exceptionally, Pischel et al. demonstrated that (C,N) chelate dimesitylboron complexes based on arylisoquinoline ligand can be used for singlet oxygen production with a moderate singlet oxygen quantum yield value of 0.34.^19^ In addition, selected boron dipyrromethanes (BODIPYs) were proven as efficient triplet state PSs.^20−25^ Filatov et al. showed that the formation of a triplet state can be strongly accelerated in the compact donor–acceptor BODIPY dyads.^26−28^ In such systems, intramolecular photoinduced electron transfer (PeT) facilitates the formation of charge transfer state (^1^CT), which is followed by its direct conversion to the lowest triplet excited state (^3^LE) via spin–orbit charge transfer intersystem crossing (SOCT-ISC). This approach offers a lot of advantages including simple molecular design, feasible synthesis, and long triplet state lifetimes. Nonetheless, the scope of the investigated structures is mostly limited to BODIPYs bearing polyaromatic hydrocarbon (anthracene, perylene) or dibenzo(hetero)arene functionality (carbazole, phenoxazine, phenothiazine) at the *meso* position of the BODIPY frame (Chart 1).^29−40^

Taking advantage of the rapidly growing chemistry of 9-borafluorene,^41^ herein, we propose a new strategy for the structural design of the efficient triplet state photosensitizers that can be easily adopted by numerous classes of boron complexes. We have employed *spiro* 9-borafluorene complexes, where a tetracoordinated boron atom acts as a natural separator between borafluorene (donor) and orthogonally aligned ligand (acceptor) sites (Chart 1).

**Chart 2:**
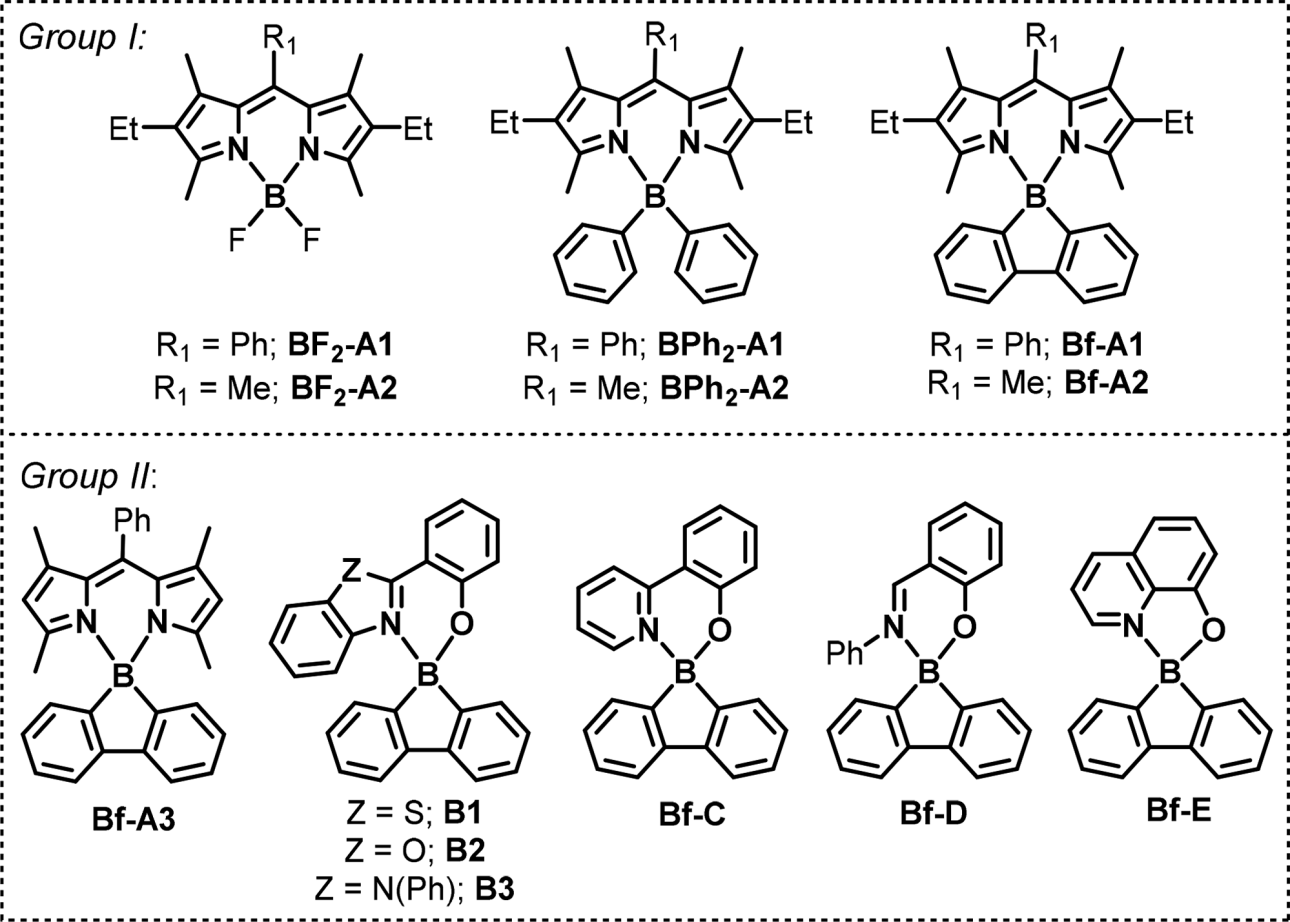
Structures of Studied PSs

In order to show the universal character of the proposed approach, our study encompasses different classes of chelate ligands selected from the large number of already known structures, taking the criteria of their literature popularity,^17,18^ structural simplicity, synthetic feasibility, or commercial availability. In order to elucidate the advancement in replacing fluorine atoms or two separated organic groups with a π-conjugated system, in the first step, we have compared the photosensitizing capabilities of respective difluoroboron (BF_2_-A1, BF_2_-A2), diphenylboron (BPh_2_-A1, BPh_2_-A2), and finally borafluorene (Bf-A1, Bf-A2) complexes (group I, Chart 2). This was followed by further studies over a larger group of borafluorene complexes constituting different ligand types (Bf-A3, Bf-B1, Bf-B2, Bf-B3, Bf-C, Bf-D, Bf-E), which allowed selection of the most active PSs (group II, Chart 2). Our studies are supplemented by theoretical calculations, which shed light on the mechanism of triplet state formation and role of the borafluorene unit. Finally, the photocatalytic properties were evaluated in singlet-oxygen mediated oxidations of model organic substrates.

## Results and Discussion

The syntheses of BODIPY dyads were performed according to the known or modified literature protocols.^42−48^ The borafluorene-based BODIPYs Bf-A1, Bf-A2, and Bf-A3 were obtained from the corresponding dipyrromethene ligand precursors (A1-H, A2-H, A3-H) and 9-chloroborafluorene in the presence of base (*i*-Pr)_2_NEt. The remaining considered borafluorene complexes were synthesized in a similar way following a procedure reported by us.^49^

**Figure 1:**
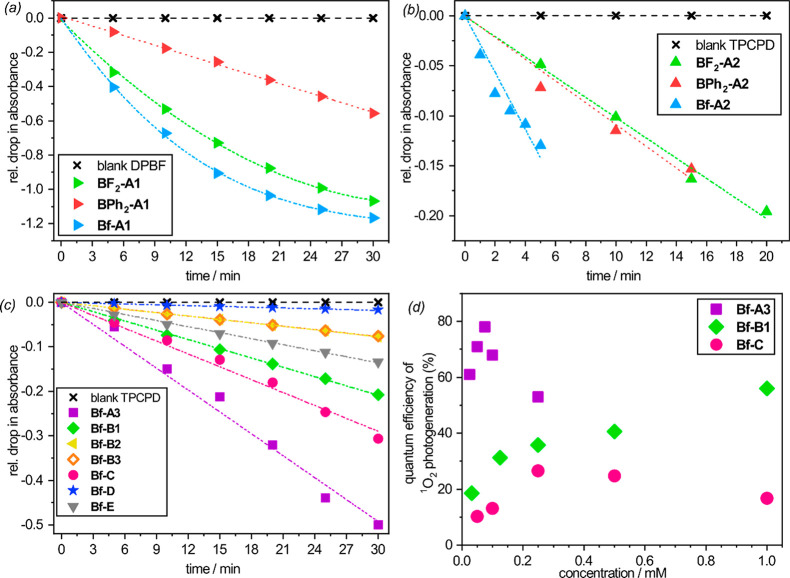
Relative drop in absorbance of (*a*) DPBF at 412 nm and TPCPD (*b*, *c*) at 510 nm. In order to avoid extensive overlapping of absorbance bands of the singlet oxygen probe and PS, A1 complexes were measured with a DPBF probe, while for remaining complexes TPCPD was used. (*d*) Singlet oxygen quantum yield as a function of concentration determined with TPCPD as a singlet oxygen probe.

We commenced our study by comparing the photosensitization activity of Bf-BODIPY complexes Bf-A1 and Bf-A2 with their difluoroboron (BF_2_-A1, BF_2_-A2) and diphenylboron (BPh_2_-A1, BPh_2_-A2) counterparts (group I, Chart 2). The singlet oxygen sensitization monitored by UV–vis spectroscopy in the presence of a singlet oxygen probe (tetraphenylcyclopentadienone, TPCPD, or 1,3- diphenylisobenzofuran, DPBF) shows the superiority of the borafluorene singlet oxygen production rate over nonannulated systems (Figure 1a,b). The singlet oxygen quantum yields (QY^O^, Table 1) for Bf-A1 and Bf-A2 are 0.39 (0.05 mM) and 0.55 (0.05 mM), respectively, which are generally higher compared to the values obtained for the corresponding difluoro- and diphenylboron complexes (Table 1, Supporting Information).

**Table 1:** Photophysical Data for the Studied Boron Complexes in DCM Solutions (λ_abs_, Absorption Wavelength; λ_em_, Emission Wavelength; QY^F^, Fluorescent Quantum Yield; QY^O^, Quantum Yield of Singlet Oxygen Generation)

complex	λ_abs_/nm	λ_em_/nm	QY^F^^a^^,^^b^	QY^O^^c^
BF_2_-A1	525	539	0.83^a^	0.37^d^^,^^h^^,^^j^^,^^m^
BPh_2_-A1	517	530	0.06^a^	0.28^d^^,^^h^^,^^j^^,^^m^
Bf-A1	520	533	0.33^a^	0.39^d^^,^^h^^,^^j^^,^^m^
BF_2_-A2	520	538	0.95^a^	0.34^d^^,^^i^^,^^j^^,^^m^
BPh_2_-A2	513	549	0.70^a^	0.15^d^^,^^i^^,^^j^^,^^m^
Bf-A2	516	539	0.75^a^	0.55^d^^,^^i^^,^^j^^,^^m^
Bf-A3	501	511	0.20^b^	0.78^e^^,^^i^^,^^j^^,^^m^
Bf-B1	400^49^	496^49^	0.22^49^	0.56^f^^,^^i^^,^^k^^,^^n^
Bf-C	362^49^	482^49^	0.28^49^	0.27^g^^,^^i^^,^^l^^,^^n^

a Rhodamine 6G (EtOH).

b Fluorescein (0.1 M NaOH).

c Concentrations were adjusted to reach maximum QY^O^:

d *c* = 0.050 mM;

e *c* = 0.075 mM;

f *c* = 1.000 mM;

g *c* = 0.250 mM. Singlet-oxygen marker:

h 1,3-diphenylisobenzofuran (DPBF);

i 2,3,4,5-tetraphenylcyclopentadienone (TPCPD). Irradiation source:

j laser 532 nm;

k laser 445 nm;

l laser 365 nm. QY^O^ in the reference to

m methylene blue;

n phenalenone.

Concordantly, this is accompanied by the decrease of fluorescent quantum yield (QY^F^) with respect to BF_2_ complexes (QY^F^(BF_2_-A1) = 0.83 vs QY^F^(Bf-A1) = 0.33; QY^F^(BF_2_-A2) = 0.95 vs QY^F^(Bf-A2) = 0.75). Notably, BPh_2_ complexes exhibit the lowest QY^O^ and QY^F^ among studied boron complexes. This, however, can be rationalized by the depopulation of singlet excited states via nonradiative deexcitation pathways resulted from free rotation of phenyl groups.

To further investigate the effectivity of singlet oxygen generation over borafluorene-based complexes, we have extended our research by various classes of (N,N) and (N,O) chelate ligands (group II, Chart 2). To our surprise, we have found that all considered Bf complexes, except Bf-D, can be used for singlet oxygen sensitization, although effectivity of this process strongly depends on the ligand electronic features (Figure 1c). Thus, for subsequent studies, we have selected the most active representatives of Bf complexes including Bf-A1, Bf-B1, and Bf-C. The most efficient singlet-oxygen photosensitizer Bf-A3 with singlet oxygen quantum yield (QY^O^) reaching 0.78 in 0.075 mM DCM solution belongs to the BODIPY family. The addition of two ethyl groups at the 2,6 position (Bf-A1) or replacement of the phenyl ring with a methyl group (Bf-A2) decreases singlet oxygen quantum yields, although they still remain at a relatively high level.

The observed ^1^O_2_ generation efficiency is concentration-dependent (Figure 1d). In the case of Bf-A3, it reaches a maximum at 0.075 M in DCM and decreases rapidly at slightly higher concentrations. This can be attributed to the strong tendency of BODIPY dyes for aggregation leading to the quenching of excited states.^50^ In addition to BODIPY’s family, Bf-B1 and Bf-C complexes have also proven to be efficient singlet oxygen PSs. Despite their lower activity in highly diluted solutions, they are significantly less vulnerable to aggregation effects, which makes them more useful in concentrate solutions. In the case of Bf-B1, the singlet oxygen quantum yields systematically increase with the concentration reaching 0.56 at 1 mM (the highest tested).

**Figure 2:**
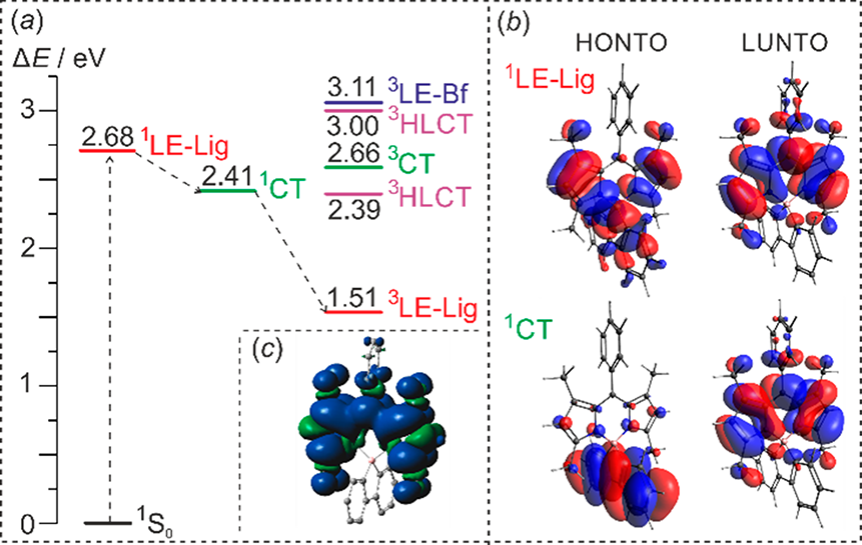
(*a*) Calculated energy diagram demonstrating the photophysical processes in Bf-A3. (*b*) Natural transition orbitals for ^1^CT and ^1^LE-Lig states. (*c*) Isosurface of spin density at optimized lowest-energy triplet state geometry ^3^LE-Lig (*iso* = 0.0004). The molecular states are abbreviated as follows: ^1^S_0_, singlet ground state; ^1^LE-Lig, singlet locally excited state on ligand site; ^3^LE-Lig, triplet locally excited state on ligand site; ^3^LE-Bf, triplet locally excited state on borafluorene site; ^1^CT, singlet charge transfer state; ^3^CT, triplet charge transfer state; ^3^HLCT, triplet hybridized local and charge transfer excited state. Color coding: locally exited state (red), charge transfer state (green), hybridized local and charge transfer state (blue). HONTO and LUNTO relate to the highest occupied and lowest unoccupied natural transition orbital, respectively.

The overall geometry of Bf complexes is determined by the rigid borafluorene core, to which planar ligands are attached almost orthogonally (Figure S38). According to our previous studies, such complexes are not entirely rigid as they possess some degree of conformational mobility related to in-plane and out-of-plane movements of ligand and borafluorene moieties as well as borafluorene-ligand twisting.^49^ Theoretical calculations (B3LYP/6-31+G(d)) performed for Bf-B1 and Bf-C reveal that HOMO–1 and LUMO are located on the ligand and are responsible for observed electron transition to a locally exited state ^1^LE-Lig (Figures S39, S40). The HOMO confined on a perpendicularly aligned borafluorene is elevated with respect to HOMO–1 by about 0.25 eV. It may contribute to the subsequent processes such as photoinduced electron transfer (PeT) leading to the population of a more stable singlet charge transfer state (^1^CT; Figure S46). In turn, in Bf-A3, the HOMO and HOMO–1 are reversed with respect to molecular sites. Nonetheless, the orbital distribution for the molecule in its excited state resembles the situation observed for Bf-B1 and Bf-C (Figure S44). Likewise, the ^1^CT state is more stable than ^1^LE-Lig (Figure 2). A different situation is observed for diphenylboron BPh_2_ complexes, where the ^1^LE-Lig state is the lowest energy excited singlet state (Figure S46) implicating that phenyl groups are not involved in photophysical processes. This is in line with their lower QY^O^ values. Accordingly, the photophysical behavior of arylisoquinoline dimesitylboron complex, studied in detail by Pischel et al., resulted from the electronic structure of the ligand, while mesityl groups were not engaged.^19^ In this sense, the conjugation of boron aromatic groups is a key factor defining the photophysical behavior of borafluorene dyads, which distinguishes them from difluoroboron and other nonannulated organoboron systems.

The calculations on spin density distribution and natural transition orbital (NTO) analysis indicate that the most stable triplet state presumably responsible for observed singlet oxygen generation is localized on the ligand (^3^LE-Lig; Figures S48–S55). The energy of this state for Bf-B1 and Bf-C equals 2.11 and 2.38 eV, respectively, and it significantly decreases to 1.51 eV for Bf-A3. We suppose that ISC may occur between ^1^CT and ^3^LE-Lig according to a similar mechanism operating in compact donor–acceptor BODIPY dyads (i.e., SOCT-ISC). It is facilitated by the change of the total angular momentum, which compensates for the change in electron spin angular momentum. However, the obtained spectroscopic results are puzzling. The emission spectra and fluorescent quantum yields are not dependent on solvent polarity as typically observed for donor–acceptor BODIPYs featuring SOCT-ISC (Table S1). Thus, further studies are required to deeply understand the mechanism behind observed photoactivations.

In the next step, Bf-A3, Bf-B1, and Bf-C complexes were employed as photosensitizers for ^1^O_2_-mediated oxidation of selected organic molecules including 2-furoic acid (FA), 9,10-dimethylanthracene (DMA), thioanisole (PhSMe), and triphenylphosphine (PPh_3_). To ensure stable reaction conditions (same light intensity, reaction temperature, and air accessibility), we have constructed a photoreactor designed for small-scale photooxidation test reactions (Figure S7). Photocatalysts were irradiated with white (Bf-A3), 395 nm UV (Bf-B1), and 365 nm UV (Bf-C) 26 W LED stripes in line with their absorbance maxima. Conversion was monitored by ^1^H NMR (Figures S16–S19).

We commenced our exploration with optimization of the solvent and photocatalyst loading. The optimal activity of Bf-A3 was achieved at the minimal 0.05 mol % loading with respect to the substrate (Figure S13). To ensure comparability of results, the remaining complexes were tested using the same loading (0.05 mol %). We have also performed test reactions with commercially available photocatalysts: Rose Bengal (RB) and 5,10,15,20-tetraphenylporphyrin (TPP), both PSs, were irradiated with white 26 W LED stripes. The solvent optimization indicates that oxidation of FA and PPh_3_ is strongly favored in polar solvents (Figures S9–S11). In contrast, the highest conversion rates for DMA were observed in CHCl_3_, while the oxidation of thioanisole is the most efficient in a 2:1 MeOH/MeCN mixture.

**Figure 3:**
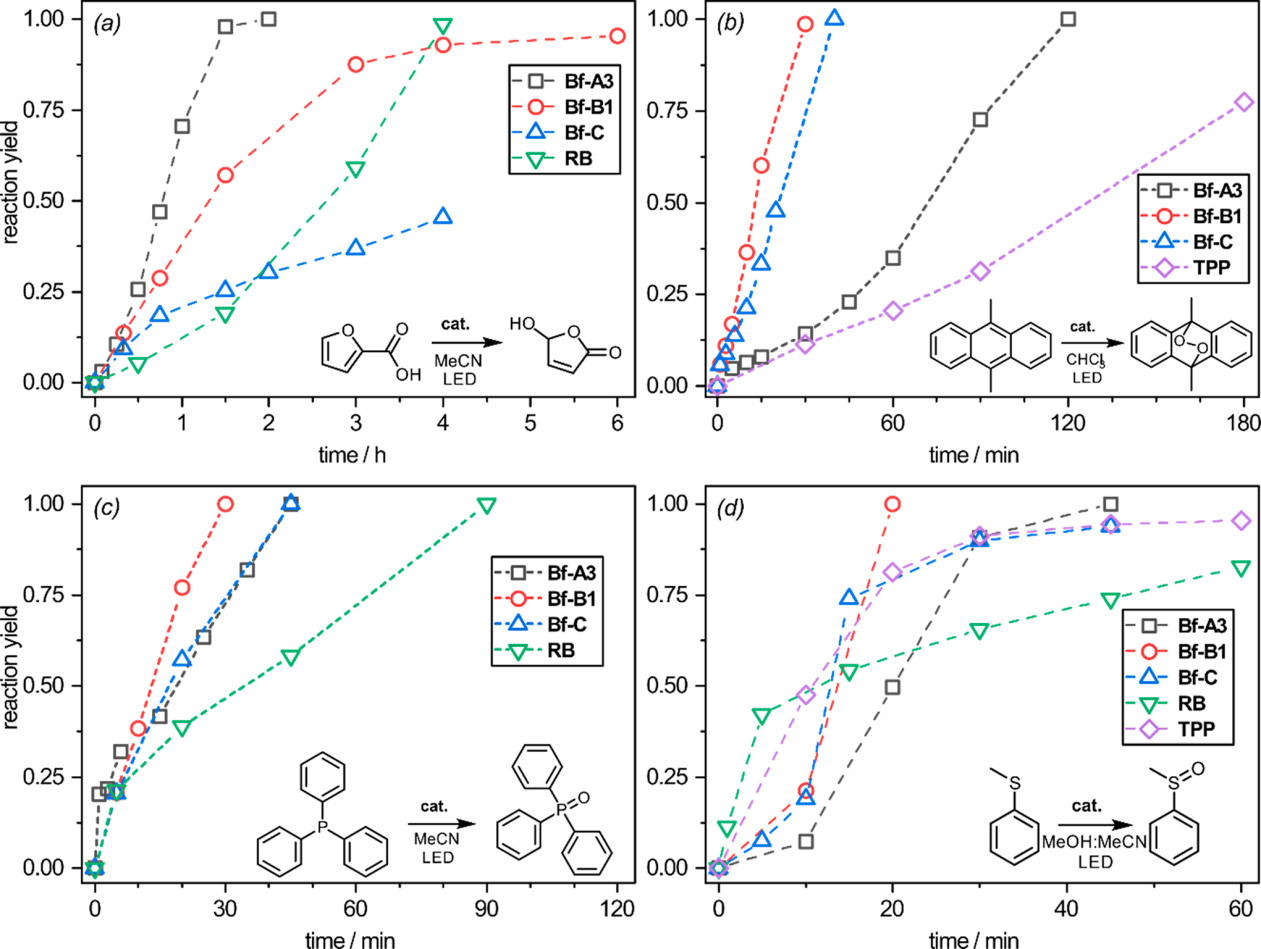
(*a*) Reaction profiles for the catalytic oxidation of (*a*) FA; (*b*) DMA; (*c*) PPh_3_; (*d*) PhSMe with Bf-A3 (gray square), Bf-B (red circle), Bf-C (blue triangle up), RB (green triangle down), and TPP (purple diamond). Reaction profiles for FA and DMA oxidation with TPP and RB, respectively, were not determined due to the problems associated with their solubility in MeCN (TPP) and CHCl_3_ (RB). Conditions: *T* = 25 °C; constant stirring and irradiation; substrate concentration *c* = 12 mg/mL, 0.05% mol PS. Irradiation source: white (Bf-A3, RB, TPP), 395 nm UV (Bf-B1), and 365 nm UV (Bf-C) 26 W LED stripes. Solvent: FA (MeCN), DMA (CHCl_3_), PPh_3_ (MeCN), PhSMe (MeOH/MeCN 2:1). Numerical data are stored in Table 2.

Having established optimal reaction conditions, we have conducted photocatalytic oxidation of FA, DMA, PhSMe, and PPh_3_ with borafluorene complexes and commercial photocatalysts (Figure 3). Our results show that the Bf-A3 complex quantitatively oxidize 2-furoic acid to 5-hydroxyfuran-2(5H)-one within 2 h with a turnover frequency (TOF) of 1000 h^–1^, which outperforms both RB and TPP (Table 2). The remaining photocatalysts were less effective, reaching a conversion of 95% with Bf-B1 and 45% with Bf-C after 4 h. The reaction of singlet oxygen with DMA proceeds according to the same mechanism as oxidation of FA ([4 + 2] cycloaddition). Nevertheless, substantial differences were observed within the tested group of PSs. To our surprise, Bf-A3 was less efficient toward DMA oxidation than Bf-B1 and Bf-C. The latter systems quantitatively convert DMA within only 30–40 min. Notably, the process is partially limited by air diffusion; with more vigorous stirring and turbulent air flow, the reaction can be completed within 15 min (TOF = 8000 h^–1^).

**Table 2:** Photooxidation Reactions Promoted by Bf-A3, Bf-B1, Bf-C, and Commercially Available RB and TPP under Optimized Conditions

substrate	PS	solvent	time/min	conversion^a^/%	TOF/h^–1^
FA	Bf-A3	MeCN	120	100	1000
	Bf-B1	MeCN	240	90	450
	Bf-C	MeCN	240	45	225
	RB	MeCN	360	99	495
DMA	Bf-A3	CHCl_3_	120	100	1000
	Bf-B1	CHCl_3_	30	99	3950
	Bf-C	CHCl_3_	40	100	3000
	TPP	DCM	360	100	335
PhSMe	Bf-A3	MeCN/ MeOH (1:2)	60	100	2000
	Bf-B1	MeCN/ MeOH (1:2)	20	100	6000
	Bf-C	MeCN/ MeOH (1:2)	30	94	3755
	RB	MeOH	60	100^b^	1660
	TPP	MeCN/ MeOH (1:2)	45	96	2550
PPh_3_	Bf-A3	MeCN	45	100	2665
	Bf-B1	MeCN	30	100	4000
	Bf-C	MeCN	45	100	2665
	RB	MeCN	90	100	1335
	TPP	DCM	120	100	1000

a Conditions: *T* = 25 °C; constant stirring and irradiation; substrate concentration: *c* = 12 mg/mL, 0.05% mol PS. Irradiation source: white (Bf-A3, RB, TPP), 395 nm UV (Bf-B1), and 365 nm UV (Bf-C) 26 W LED stripes.

b 7:3 sulfoxide to sulfone mixture was obtained.

The selective oxidation of thioanisole to (methylsulfinyl)benzene was achieved within only 20 min with Bf-B1, 30 min (94% conversion) with Bf-C, and 1 h using Bf-A3 in 2:1 MeOH/MeCN. For comparison, >95% conversion was obtained after 2 h with TPP and 1 h with RB (MeOH), albeit photooxidation with RB is less selective, giving a 7:3 mixture of sulfoxide (PhS(O)Me) and sulfone (PhS(O)_2_Me). Finally, the rate of PPh_3_ photooxidation was comparable for all three Bf complexes with 100% conversion achieved in 30–45 min, while RB and TPP were proven to be less efficient with full conversion achieved in 1.5 and 2 h, respectively.

Due to the susceptibility of the B–C bond toward oxidation as well as the labile character of the B–N dative bond, organoboron complexes can be vulnerable to hydrolytic and photochemical degradation. Furthermore, electron-rich functional groups and π-conjugated systems are prone to oxidation with ^1^O_2_. This can be a serious drawback of previously studied *meso*-functionalized BODIPYs featuring SOCT-ISC. Therefore, we have evaluated the photochemical and hydrolytic stability of all studied compounds, including the reference catalysts (Table 3). Obtained results demonstrate that the stability of BODIPYs Bf-A3 is the highest among studied complexes with half-life times (*t*_0.5_) of 529 min in CHCl_3_, 264 min in MeCN, and 1056 min in MeOH/MeCN (2:1). In comparison, 2,6-diodinated BODIPY derivatives half-decompose after 30–60 min of irradiation.^51^ Bf-C with a *t*_0.5_ of 208 min in CHCl_3_ and 251 min in MeCN is also sufficiently stable. Both complexes were resistant toward hydrolysis, regardless of the solvent used. In turn, fast decomposition of Bf-B1 with a *t*_0.5_ of 17 and 72 min accelerated by its hydrolysis was observed in CHCl_3_ and MeCN, respectively.

**Table 3:** Calculated Half-Time Stability Values (*t*_0.5_) for Bf-A3, Bf-B1, Bf-C, RB, and TPP upon Irradiation and in Darkroom (Values in Italic). Absorption maxima used for the *t*_0.5_ stimation are provided in brackets

	MeCN	CHCl_3_	MeOH/MeCN (2:1)
Bf-A3	264 (497)	529 (501)	1056 (498)
	*stable (497)*	*stable (501)*	*stable (498)*
Bf-B1	72 (396)	17 (400)	20 (393)
	207 (396)	*stable (400)*	490 (393)
Bf-C	251 (356)	208 (362)	283 (352)
	*stable (356)*	*stable* (362)	*stable* (352)
RB	35 (557)	a	278 (555)^b^
	*stable (557)*		5753 (555)^b^
TPP	a	967 (418)	1392 (415)
		*stable (418)*	*stable (415)*

a Compound is not soluble in given solvent.

b Pure MeOH was used due to the low solubility of the compound in MeOH/MeCN mixture.

## Conclusions

In conclusion, we have introduced a new class of heavy-atom free triplet state photosensitizers based on borafluorene–ligand dyads. The proposed conception benefits from the natural separation of the ligand and organoboron moieties through a boron node that allows the liberation of the ligand from simultaneous donor–acceptor role. We have demonstrated that the latter can be selected from a plethora of already known structures including popular (N,N) and (N,O) chelate ligands going beyond BODIPY compounds. Such structural diversification would also allow circumvention of the common problems of BODIPY dyes associated with aggregation effects. At this point, it is important to note that the structure of the ligand neither is specific nor requires special functionalization, which was one of the criteria met in previously studied heavy-atom free boron photosensitizers. The photophysical behavior of Bf-dyads mostly results from the electronic features of borafluorene donor and orthogonal alignment of donor and acceptor moieties, all together enhancing intersystem crossing to the triplet state. The photo-oxidation experiments clearly show that borafluorene dyads are 2- to 3-times more effective than common photosensitizers. We believe that this approach would allow reinvestigation of the large family of boron complexes as potent triplet state photosensitizers, which would provide inspiration in exploring their further usability in PDT and heterogeneous catalytic processes.

## Experimental Section

### General Comment

All the used reagents were purchased from Merc, TCI, and Alfa Aesar. DCM, hexane, and THF were purified using MBraun SPS and stored over 3 Å molecular sieves. Starting materials (substituted pyrroles, *n*-BuLi (2.5 M in hexane), acetyl chloride, benzaldehyde, *p*-toluenesulfonic acid monohydrate (*p*-TsOH), 2,3-dichloro-5,6-dicyano-1,4-benzoquinone (DDQ), (*i*-Pr_2_)NEt, BF_3_·OEt, NEt_3_, and bromobenzene were used as received without additional purification. Reactions and manipulations involving air and moisture-sensitive reagents were carried out under an argon atmosphere. Except Bf-A1 and Bf-A2, the syntheses of BODIPYs were performed according to slightly modified literature procedures. The syntheses of B-phenyl BODIPYs BPh_2_-A1 and BPh_2_-A2 were described previously by Thompson et al. and utilize the selective substitution of fluorine atoms attached to the boron atom with phenyl groups using a phenylmagnesium bromide reagent.^45^ A similar system comprising a 4-methylphenyl substituent located at the *meso* position of the BODIPY frame, namely, 2,6-diethyl-4,4-diphenyl-1,3,5,7-tetramethyl-8-(4-methylphenyl)-4-bora-3a,4a-diaza-s-indacene, was obtained by Kubota et al.^52^ using excess phenyllithium. However, we decided to follow Thompson’s procedure due to its higher selectivity. The syntheses of the remaining borafluorene complexes (Bf-B1, Bf-B2, Bf-B3, Bf-C, Bf-D, and Bf-E) were described by us recently.^49^ We already have the necessary amounts of all samples.

^1^H, ^11^B, and ^13^C NMR spectra were recorded on Bruker Advance III 300 MHz and Agilent NMR 400 MHz DDR2 spectrometers. ^1^H and ^13^C chemical shifts were referenced to TMS using known chemical shifts of solvent residual peaks. ^11^B NMR chemical shifts are given relative to BF_3_·Et_2_O. In the ^13^C NMR spectra, the resonances of boron-bound carbon atoms were not observed in most cases as a result of their broadening by the quadrupolar boron nucleus. Deuterated solvents were dried with 3 Å molecular sieves, and samples were prepared under an argon atmosphere. HR-MS analyses were performed on a GCT Premier mass spectrometer equipped with an EI ion source and a Maldi SYNAPT G2-S HDMS spectrometer equipped with an ESI ion source.

### Synthesis

#### (*Z*)-3-Ethyl-5-[(4-ethyl-3,5-dimethyl-2H-pyrrol-2-ylidene)phenylmethyl]-2,4-dimethyl-1H-pyrrole, A1-H^42^

Benzaldehyde (1.5 mL, 15 mmol) was added to a solution of 3-ethyl-2,4-dimethylpyrrole (4 mL, 30 mmol) in DCM (100 mL) at room temperature (r.t.). The solution turned deep yellow. After stirring for 10 min, *p*-TsOH was added (*ca.* 10 mg). The mixture was stirred overnight at r.t. A DDQ (3.12 g, 14 mmol) suspension in DCM (40 mL) was added to the reaction mixture and stirred for 30 min. The reaction was quenched with water (30 mL), and after vigorous stirring, phases were separated. The organic layer was washed with brine and dried over anhydrous MgSO_4_. A deep red solution was filtered, and solvents were removed under a vacuum. The crude product was purified using column chromatography with Al_2_O_3_ as a stationary phase and a DCM eluent. The obtained residue was washed with hexane to form an almost black greenish solid; yield, 2.32 g (47%). ^1^H NMR (300 MHz, CDCl_3_, ppm): δ 13.02 (s, 1H), 7.46–7.41 (m, 3H), 7.35–7.31 (m, 2H), 2.35 (bs, 6H), 2.30 (q, *J* = 7.5 Hz, 4H), 1.22 (bs, 6H), 1.00 (t, *J* = 7.6 Hz, 6H).

#### 2,6-Diethyl-1,3,5,7-tetramethyl-8-phenyl-4,4-difluoro-4-bora-3a,4a-diaza-s-indacene, BF_2_-A1^43^

(*i*-Pr)_2_NEt (7.2 mL, 42 mmol) was added to a solution of A1-H (2.32 g, 7 mmol) in DCM (100 mL) at r.t. After stirring for 30 min, BF_3_OEt_2_ (7.8 mL, 63 mmol) was added dropwise. The mixture was stirred, overnight at r.t. The reaction was quenched with water (30 mL) and after vigorous stirring, phases were separated. The organic layer was washed with saturated NaHCO_3_ solution (3 × 30 mL) and dried with anhydrous MgSO_4_. The dark red solution was filtered, and solvents were removed under a vacuum. The crude product was purified using column chromatography (hexane/CHCl_3_ = 1:1). The obtained residue was washed with hexane to form a burgundy powder; yield, 0.54 g (20%). ^1^H NMR (400 MHz, CDCl_3_, ppm): δ 7.46–7.49 (m 3H), 7.31–7.22 (m, 2H), 2.53 (s, 6H), 2.30 (q, *J* = 7.6 Hz, 4H), 1.27 (s, 6H), 0.98 (t, *J* = 7.6 Hz, 6H).

#### 2,6-Diethyl-1,3,5,7,8-pentamethyl-4,4-difluoro-4-bora-3a,4a-diaza-s-indacene, BF_2_-A2^44^

Acetyl chloride (1.8 mL, 25 mmol) was added to a solution of 3-ethyl-2,4-dimethylpyrrole (6.8 mL, 50 mmol) in DCM (60 mL). The solution turned deep red. The mixture was refluxed using a water bath in an argon atmosphere for 2.5 h. After cooling to room temperature, Et_3_N (20 mL, 140 mol) was added. After 10 min of stirring, the mixture was diluted with DCM (30 mL), and BF_3_OEt_2_ (27 mL, 220 mol) was added dropwise at r.t. The mixture was stirred overnight, extracted with saturated NaHCO_3_ solution (3 × 30 mL), and dried with anhydrous Na_2_SO_4_. After filtration, volatiles were removed under a vacuum to obtain a dry solid. The crude product was purified using column chromatography (hexane/CHCl_3_ = 1:1). The obtained residue was washed with hexane to form an orange powder, yielding 3.12 g (39%). ^1^H NMR (300 MHz, CDCl_3_, ppm): δ 2.61 (s, 9H), 2.42 (q, *J* = 7.6 Hz, 4H), 2.35 (s, 6H), 1.06 (t, *J* = 7.6 Hz, 6H).

#### 2,6-Diethyl-1,3,5,7-tetramethyl-4,4,8-triphenyl-4-bora-3a,4a-diaza-s-indacene, BPh_2_-A1^45^

To a suspension of Mg (0.16 g, 6 mmol) in Et_2_O (5 mL) with a catalytic amount of I_2_ (*ca.* 6 mg), a solution of bromobenzene (0.67 mL, 6 mmol) in Et_2_O (5 mL) was added dropwise. After adding *ca.* 1/3 of the solution at room temperature, the reaction mixture was warmed up to reflux using a water bath to initiate the reaction, and dropwise addition of the remaining bromobenzene solution was continued, maintaining a gentle reflux. The mixture was refluxed until all Mg dissolved. The obtained mixture cooled to 0 °C, and a cooled (0 °C) solution of BF_2_-A1 (0.24 g, 0.6 mmol) in DCM (50 mL) was added dropwise. The solution was stirred for 1 h at r.t. The reaction was quenched with water (20 mL), filtered, and dried over anhydrous MgSO_4_. After filtration, volatiles were removed under a vacuum. The obtained solid was washed with hexane to form a burgundy powder, yielding 90 mg (29%). ^1^H NMR (400 MHz, CDCl_3_, ppm): δ 7.47 (dd, *J* = 5.1, 1.9 Hz, 3H), 7.44–7.37 (m, 4H), 7.38–7.31 (m, 2H), 7.27–7.20 (m, 3H), 7.21–7.15 (m, 2H), 2.21 (q, *J* = 7.5 Hz, 4H), 1.77 (s, 6H), 1.30 (s, 6H), 0.89 (t, *J* = 7.5 Hz, 6H).

#### 2,6-Diethyl-4,4-diphenyl-1,3,5,7,8-pentamethyl-4-bora-3a,4a-diaza-s-indacene, BPh_2_-A2^46^

Compound BPh_2_-A2 was prepared and purified as described for BPh_2_-A1 using Mg (0.24 g, 10 mmol), I_2_ (*c.a.* 10 mg), Et_2_O (5 mL), bromobenzene (1 mL, 10 mmol), and BF_2_-A2 (0.32 g, 1 mmol) in DCM (50 mL). The reaction yielded a rust-like crystalline product, 250 mg (58%). ^1^H NMR (400 MHz, CDCl_3_, ppm): δ 7.25–7.12 (m, 10H), 2.66 (s, 3H), 2.38–2.30 (m, 10H), 1.70 (s, 6H), 0.96 (t, *J* = 7.5 Hz, 6H).

#### 2′,8′-Diethyl-1′,3′,7′,9′-tetramethyl-10′-phenyl-4λ^4^,5λ^5^-spiro[dibenzo[b,d]borole-5,5′-dipyrrolo[1,2-c:2′,1′-*f*][1,3,2]diazaborinine], Bf-A1

(*i*-Pr)_2_NEt (0.5 mL, 3 mmol) was added to a solution of A1-H (0.94 g, 3 mmol) in DCM (100 mL) at r.t. After cooling to 0 °C, the mixture was stirred for 25 min, and molten (∼40 °C) 9-chloroborafluorene (0.43 mL, 3 mmol) was added from a syringe in one portion. ***Caution!**9-Chloroborafluorene rapidly hydrolyzes followed by decomposition in contact with water and oxygen.* The mixture was stirred overnight at r.t. The reaction was quenched with water (20 mL), filtered, and dried over anhydrous MgSO_4_. After filtration, volatiles were removed under a vacuum to obtain a solid. The crude product was purified using column chromatography (hexane/PhMe = 1:1). The obtained residue was washed with hexane to form an orange powder, yielding 0.31 g (21%). Mp > 350 °C. HRMS (ESI) calcd. for C_35_H_35_BN_2_ [M^+^]: 494.2888. Found: 494.2885. ^1^H NMR (400 MHz, CDCl_3_, ppm): δ 7.68–7.61 (m, 2H), 7.58–7.48 (m, 3H), 7.45 (dd, *J* = 7.7, 1.8 Hz, 2H), 7.28–7.22 (m, 4H), 7.11 (td, *J* = 7.3, 1.1 Hz, 2H), 2.14 (q, *J* = 7.6 Hz, 4H), 1.46 (s, 6H), 1.32 (s, 6H), 0.83 (t, *J* = 7.5 Hz, 6H). ^13^C{1H} NMR (101 MHz, CDCl_3_, ppm): δ 152.6, 150.4, 140.4, 137.0, 135.1, 132.5, 130.6, 130.3, 128.9, 128.8, 128.4, 127.1, 127.0, 118.7, 17.2, 14.7, 12.0, 11.9. ^11^B NMR (96 MHz, CDCl_3_, ppm): δ −0.3.

#### 2′,8′-Diethyl-1′,3′,7′,9′,10′-pentamethyl-4λ^4^,5λ^5^-spiro[dibenzo[b,d]borole-5,5′-dipyrrolo[1,2-c:2′,1′-*f*][1,3,2]diazaborinine], Bf-A2

Acetyl chloride (0.4 mL, 6 mmol) in DCM (3 mL) was added to a solution of 3-ethyl-2,4-dimethylpyrrole (0.4 mL, 3 mmol) in DCM (5 mL). The mixture was refluxed using a water bath in an argon atmosphere for 1 h. After cooling to room temperature, the mixture was concentrated under a vacuum to 1/3 of the initial volume. Petroleum ether was added to precipitate a dark residue, and the mixture was stirred intensively for 30 min. Stirring was turned off, and after a few minutes the solution was decanted, and the obtained oily residue was dissolved in DCM (5 mL). Molten 9-chloroborafluorene (0.40 mL, 3 mmol) and NEt_3_ (0.5 mL, 4 mmol) were added to the mixture. The solution was stirred for 20 min, extracted with saturated NaHCO_3_ solution (3 × 10 mL), and dried with anhydrous Na_2_SO_4_. After filtration, volatiles were removed under a vacuum to obtain a dry solid. The crude product was purified using column chromatography (hexane/PhMe = 2:3). The obtained residue was washed with hexane to form a vermilion powder, yielding 0.25 g (11%). Mp > 350 °C. HRMS (EI+) calcd. for C_30_H_33_BN_2_ [M^+^]: 432.2737. Found: 432.2746. ^1^H NMR (300 MHz, CDCl_3_, ppm): δ 7.62 (dt, *J* = 7.5, 0.9 Hz, 2H), 7.23 (td, *J* = 7.3, 1.5 Hz, 2H), 7.17–7.00 (m, 4H), 2.79 (s, 3H), 2.41 (s, 6H), 2.25 (q, *J* = 7.5 Hz, 4H), 1.43 (s, 6H), 0.90 (t, *J* = 7.6 Hz, 6H). ^13^C{1H} NMR (75 MHz, CDCl_3_, ppm): δ 151.0, 150.6, 139.8, 133.2, 132.3, 132.0, 130.3, 127.1, 118.8, 17.8, 17.4, 15.1, 15.0, 12.2. ^11^B NMR (96 MHz, CDCl_3_, ppm): δ −0.6.

#### (*Z*)-5-[(3,5-Dimethyl-2H-pyrrol-2-ylidene)phenylmethyl]-2,4-dimethyl-1H-pyrrole, A3-H^47^

Compound A3-H was prepared and purified as described for A1-H using 2,4-dimethylpyrrole (2 mL, 20 mmol), benzaldehyde (1 mL, 10 mmol), *p*-TsOH (*ca.* 5 mg), and DDQ (2.4 g, 11 mmol) in DCM (100 mL). The reaction yielded a black powder (0.8 g, 29%). ^1^H NMR (400 MHz, CDCl_3_, ppm): δ 7.42 (dd, *J* = 5.1, 1.9 Hz, 3H), 7.33–7.28 (m, 2H), 5.89 (d, *J* = 1.1 Hz, 2H), 2.35 (s, 6H), 1.29 (d, *J* = 1.0 Hz, 6H).

#### 1′,3′,7′,9′-Tetramethyl-10′-phenyl-4λ^4^,5λ^5^ spiro[dibenzo[b,d]borole-5,5′-dipyrrolo[1,2-c:2′,1′-*f*][1,3,2]d azaborinine], Bf-A3^48^

Compound Bf-A3 was prepared as described for Bf-A1 using A3-H (0.8 g, 3 mmol), (*i*-Pr)_2_NEt (0.5 mL, 3 mmol), and 9-chloroborafluorene (0.45 mL, 3 mmol) in DCM (40 mL). The crude product was purified using column chromatography (hexane/PhMe = 2:3). The reaction yielded a red crystalline product (0.08 g, 6%). ^1^H NMR (400 MHz, CDCl_3_, ppm): δ 7.65–7.60 (m, 2H), 7.58–7.47 (m, 3H), 7.47–7.42 (m, 2H), 7.30–7.21 (m, 4H), 7.11 (ddd, *J* = 7.4, 6.9, 1.1 Hz, 2H), 5.82 (s, 2H), 1.49 (s, 6H), 1.42 (s, 6H).

### Singlet-Oxygen Generation Studies

Singlet-oxygen generation studies were performed using 1,3-diphenylisobenzofuran (DPBF) or tetraphenylcyclopentadienone (TPCPD) as a chemical trap in DCM, at a 0.1 mM concentration of the analyzed photosensitizer. In general, TPCPD is considered a more reliable singlet oxygen probe; however, in the case of BF_2_-A1, BPh_2_-A1, and Bf-A1 complexes, DPBF was used, and the absorption bands of PS and TPCPD significantly overlap. The sample was constantly irradiated by a xenon lamp equipped with a 500 nm filter. The irradiation direction was perpendicular with respect to a UV–vis light source. The whole process was monitored *in situ*. The full UV–vis spectrum of the mixture was collected in even time periods. A drop in absorbance of the trap was observed as the experiment proceeded. When DPBF was used, a drop was observed at 412 nm, for TPCPD at 510 nm. The determination of quantum yield of singlet oxygen generation was performed in a similar manner, with Oxxius diode lasers (365 nm, 445 nm, 532 nm) as the irradiation source. Methylene Blue (MB)^53,54^ or phenalenone (PN)^55^ was used as a QY^O^ reference. The singlet oxygen quantum yield was calculated via the relative method, the so-called DPBF-method, with the following equation:
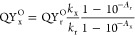
where x and r stand for substances under study and reference, respectively, QY^O^ is the singlet oxygen quantum yield, *k* denotes the DPBF or TCPCD consumption rates, and *A* represents the absorbance of the investigated or reference photosensitizer at the excitation wavelength.

### Catalytic Reactions

All catalytic reactions were performed using a homemade photoreactor (a detailed description is provided in the SI). Reactions were performed in open-air 4-mL flasks. The irradiation source was selected to best fit the absorption range of the photocatalyst. The concentrations of FA, PhSMe, PPh_3_, and DMA was 12 mg/mL (CHCl_3_, MeCN, MeOH, or MeOH/MeCN 2:1 solutions). In most cases, the amount of photocatalyst was set to 0.05%_mol_ with respect to the substrate. Reaction progress was monitored by ^1^H NMR spectra analysis of the reaction mixture sampled after a given time.

## Supporting Information Available

UV–Vis and fluorescent spectra; photocatalytic reaction details and results; photostability studies; X-ray structural data; DFT calculation results (PDF)
Theoretical structures obtained from DFT calculations (XYZ)

## Supplementary Material

jo1c01254_si_001.pdf

jo1c01254_si_002.xyz

## Accession Codes

CCDC 2068807–2068810 contain the supplementary crystallographic data for this paper. These data can be obtained free of charge via www.ccdc.cam.ac.uk/data_request/cif, or by emailing data_request@ccdc.cam.ac.uk, or by contacting The Cambridge Crystallographic Data Centre, 12 Union Road, Cambridge CB2 1EZ, UK; fax: +44 1223 336033.
