# Supplementary material for: Heavy-Atom Free spiro Organoboron Complexes As Triplet Excited States Photosensitizers for Singlet Oxygen Activation
Source: J Org Chem. 2021 Sep 1;86(18):12714–22. doi: 10.1021/acs.joc.1c01254 (PMC8453631; doi:10.1021/acs.joc.1c01254)
Supplement: Supplementary file 1 — jo1c01254_si_001.pdf [file jo1c01254_si_001.pdf]

Heavy-atom free *spiro* organoboron complexes as triplet excited states photosensitizers for singlet oxygen activation

Paulina H. Marek-Urban[a,b], Mateusz Urban[a], Magdalena Wiklińska[a], Klaudia Paplińska[c], Krzysztof Woźniak[b], Agata Blacha-Grzechnik*[c], Krzysztof Durka*[a]

[a] Warsaw University of Technology, Faculty of Chemistry, Noakowskiego 3, 00-664 Warsaw, Poland.
[b] University of Warsaw, Faculty of Chemistry, Pasteura 1, 02-093 Warsaw, Poland.
[c] Faculty of Chemistry, Silesian University of Technology, Strzody 9, 44-100 Gliwice, Poland.

SUPPORTING INFORMATION

1. Absorption and emission spectra S2
2. Singlet-oxygen generation studies S5
3. Photocatalytic setup S6
4. Catalytic reactions S8
5. Photocatalytic stability S15
6. Crystal structures S22
7. Theoretical calculations S24
8. NMR spectra S64
9. References for Supporting Information. S71

1. Absorption and emission spectra

Absorption spectra were recorded using Hitachi U-2800 spectrophotometer. Emission and fluorescence quantum yields were recorded using spectrofluorometer Edinburgh FS5. The measurements were performed at room temperature, according to published procedures.[1,2] Suprasil quartz cuvettes (10.00 mm) were used. 1.5 nm slits were used for absorption and 2.5 nm slits were used for emission spectra. To eliminate any background emission, spectrum of pure solvent was subtracted from the samples' spectra. QY^F were determined in diluted solutions (A < 0.1 for longest wavelength band) by comparison with known standards – rhodamine 6G (EtOH, $QY^F{}_r = 0.94$)[3], fluorescein (0.1 M NaOH, $QY^F{}_r = 0.95$)[4], coumarin 153 (EtOH, $QY^F{}_r = 0.54$)[5], quinine sulfate (0.1 M $HClO_4$, $QY^F{}_r = 0.60$)[6] Concentrations were in the range of $0.5\text{-}2 \cdot 10^{-5}$ moldm^{-3}. Concentrations were adjusted to reach similar absorbance to absorbance of reference solution at the excitation wavelength. Fluorescence quantum yield was determined using reference substance using following formula:

$$\mathrm{QY}_\mathrm{x}^\mathrm{F} = \mathrm{QY}_\mathrm{r}^\mathrm{F} \cdot \frac{F_\mathrm{x}}{F_\mathrm{r}} \cdot \frac{1 - 10^{-A_\mathrm{r}}}{1 - 10^{-A_\mathrm{x}}} \cdot \frac{n_\mathrm{x}^2}{n_\mathrm{r}^2}$$

where:

where ***F*** is the relative integrated photon flux of sample (**x**) and reference (**r**), ***A*** is the absorbance at the excitation wavelength, ***n*** is the refractive index of used solvents. Photon fluxes (***F***) were calculated by integration of corrected spectra ($\boldsymbol{I_c}$), obtained by (***I***) division of intensity of emission spectra by the spectral responsivity (**s**) in corresponding wavelengths (λ_{em}). All measurements were carried out at room temperature.

$$F = \int I_\mathrm{c}\, d\lambda_\mathrm{em} = \int \frac{I(\lambda_\mathrm{em})}{s(\lambda_\mathrm{em})} d\lambda_\mathrm{em}$$

Spectroscopic data (measured and taken from literature) are stored in Table S1.

Table S1. Basic photophysical data for the studied boron complexes (ε – dielectric constant, λ_{abs} – absorption wavelength, λ_{em} – emission wavelength , QY^F – fluorescent quantum yield).

Complex	solvent	ε	λ_{abs} / nm	λ_{em} / nm	QY^F
**BF_2-A1**	hexane	1.9	524[7]	535[7]	0.69[7]
	**DCM**	8.9	525	539	0.83[a]
	MeCN	35.9	520[7]	533 [7]	0.92[7]
**BPh_2-A1**	THF	7.6	516[8]	530[8]	0.042[8]
	**DCM**	8.9	517	530	0.06[a]
**Bf-A1**	**DCM**	8.9	520	533	0.33[a]
**BF_2-A2**	cHex	2.0	521[9]	533.5[9]	0.87[9]
	**DCM**	8.9	520	538	0.95[a]
	MeCN	35.9	515[9]	534[9]	0.87[9]
**BPh_2-A2**	AcOEt	6.0	512[10]	550[10]	0.51[10]
	**DCM**	8.9	513	549	0.70[a]
	MeCN	35.9	510[11]	541[11]	0.53[11]
**Bf-A2**	PhMe	2.4	519	540	0.73[a]
	**DCM**	8.9	516	539	0.75[a]
	Acetone	20.7	514	536	0.68[a]
**Bf-A3**	cHex	2.0	503	512	0.23[b]
	**DCM**	8.9	501	511	0.20[b]
	EtOH	24.6	500	510	0.25[b]
	MeCN	35.9	498	507	0.21[b]
**Bf-B1**	cHex	2.0	405	499	0.15[c]
	**DCM**	8.9	400[12]	496[12]	0.22[12]
	MeCN	35.9	397	497	0.12[c]
**Bf-B2**	**DCM**	8.9	383[12]	461[12]	0.16[12]
**Bf-B3**	**DCM**	8.9	365[12]	443[12]	0.36[12]
**Bf-C**	cHex	2.0	376	480	0.17[d]
	**DCM**	8.9	362[12]	482[12]	0.28[12]
	MeCN	35.9	356	488	0.17[d]
**Bf-D**	**DCM**	8.9	409[12]	559[12]	0.003[12]
**Bf-E**	**DCM**	8.9	397[12]	513[12]	0.49[12]

QY^F in the reference to: [a]rhodamine 6G (EtOH), [b]fluorescein (0.1 M NaOH), [c]coumarin 153 (EtOH), [d]quinine sulfate (0.1 M $HClO_4$)

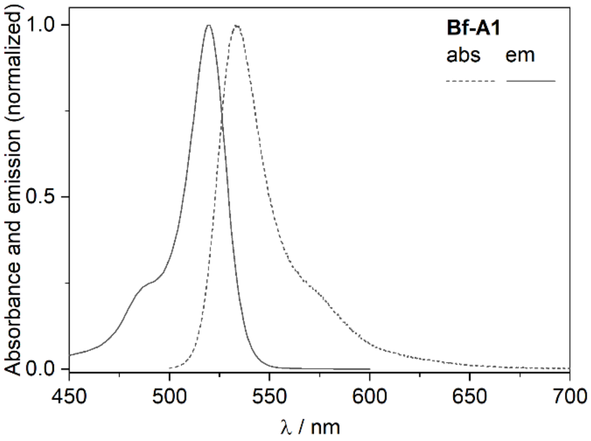

Figure S1. Normalized absorption and emission spectra of **Bf-A1** in DCM.

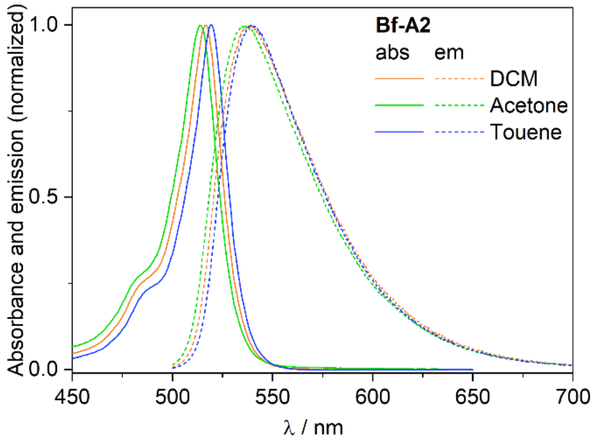

Figure S2. Normalized absorption and emission spectra of **Bf-A2** in different solvents.

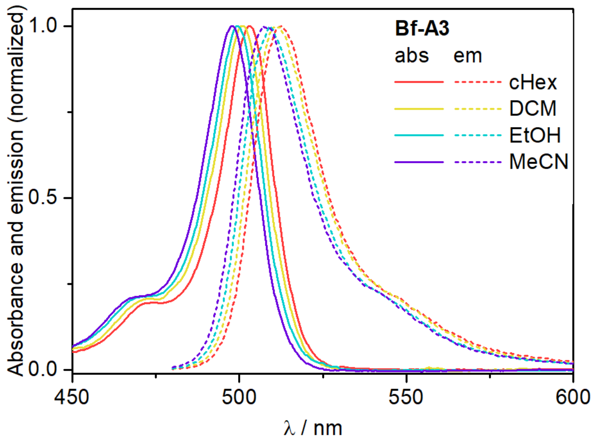

Figure S3. Normalized absorption and emission spectra of **Bf-A3** in different solvents.

2. Singlet-oxygen generation studies

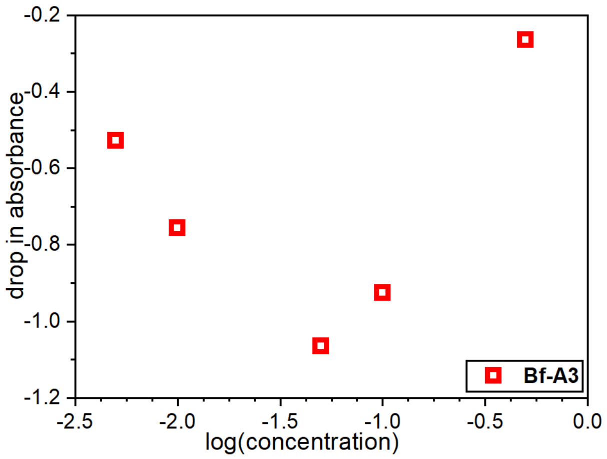

Figure S4. Drop in absorbance of **Bf-A3** at 412 nm after 10 minutes of irradiation with DPBF as a chemical trap in DCM.

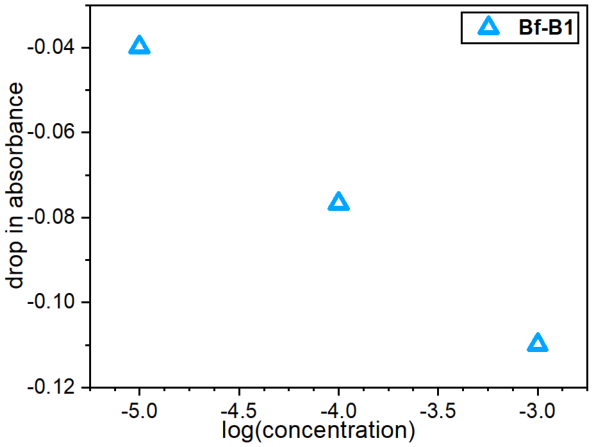

Figure S5. Drop in absorbance of **Bf-B1** at 510 nm after 10 minutes of irradiation with TPCPD as a chemical trap in DCM.

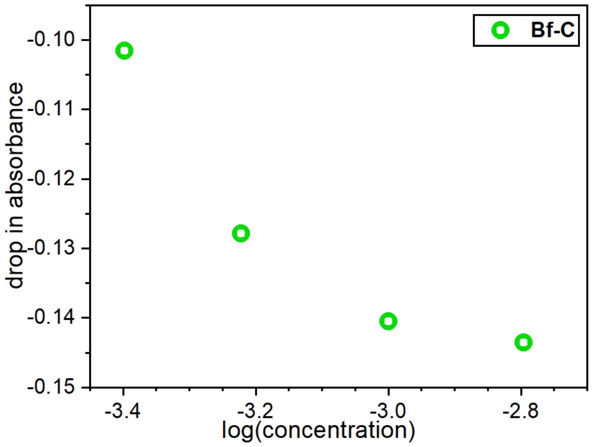

Figure S6. Drop in absorbance of **Bf-C** at 510 nm after 10 minutes of irradiation with TPCPD as a chemical trap in DCM.

3. Photocatalytic setup

To ensure stable and repeatable conditions in all performed photocatalytic reactions we have designed our home made photoreactor. It is suitable for small scale reactions in 4 ml vials, however due to its modular design it can be easily used for reactions in larger vessels (up to 50 ml). The reactor comprises of main body housing the light source, cover – sample holder and a reflective base. The photoreactor can be placed on a magnetic stirrer. Main body of the reactor is made of aluminium tube (Φ150 mm, 4 mm wall) to ensure high reflection of light and proper heat transmission. LED strips connected in parallel were glued inside the tube to achieve even distribution of light. To maintain low temperature aluminium tubes is water cooled by a copper coiled tube heat exchanger sticked to the outside wall of the reactor. To the bottom of the aluminium tube three plastic legs were attached to ensure flow of air. The main body was placed on a base – a square sheet of aluminium to ensure stable footing and reflection of stray light. As light source neutral white and UV (395 nm and 365 nm) LED strips were used. White LED strip SMD5630 (28.8 W/m, 3000 lumen/m) was purchased from www.akb-poland.com. 395 nm and 365 nm realUV^{TM} LED strips (15 W/m) were purchased from www.waveformlighting.com. White light reactors were equipped with 0.9 m LED stripes (26 W, 54 diodes) and for UV reactors were equipped with 1.7 m LED stripes (26W, 204 diodes). The cover was made of round aluminium sheet (Φ141.5 mm) comprising 8 holes for reaction vials, a fan (Φ60 mm) with air diffusor and four adjustable handles. The fan with a diffusor directing air flow to the LED strips was mandatory to disperse heat radiating from the LEDs. The samples are located possibly close to the centre of the reactor to minimise convection of heat from the LEDs. The air cooling proved necessary, as without it temperature inside the photoreactor exceeded 40 °C. As reaction vessels we have used commercially available 4 ml vials. Vial were held by the plastic screw cap. Holes in the plastic screw caps were made to ensure access of air to the reaction mixture. To ensure vigorous mixing cross shaped stirrer bars were used. Hook type handles allow adjusting of the lid position inside main body. Temperature inside reactor was controlled by placing Pt-100 thermometer into one of the reaction vials filled with water. In such conditions temperature inside the reactor oscillates close to 25 °C.

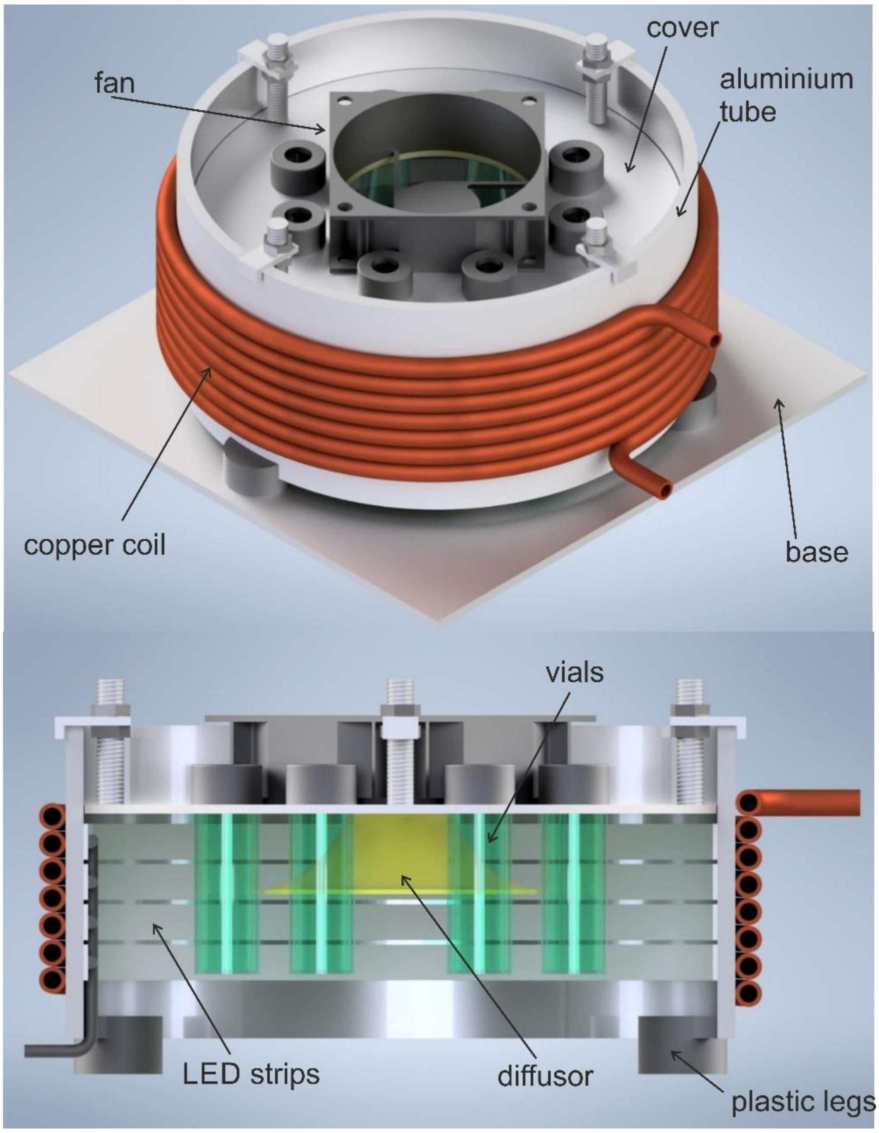

Figure S7. Model of assembled photoreactor.

4. Catalytic reactions

We have found that oxidation might be inhibited by solvent stabilizer. The oxidation of FA with **Bf-A3** in HPLC grade $CHCl_3$ containing amylene as stabilizer delivers unsatisfactory conversion of 16% after 1.5 h. However, with ACS or technical grade $CHCl_3$ containing EtOH, the conversion rose to 33%. Further addition of EtOH accelerated oxidation, with conversion reaching 49% after 1 h in 1:2 $CHCl_3$:EtOH mixture. Nonetheless, markedly increased conversion rates of FA were gained with more polar MeCN solvent. This process was, however, unaffected by addition of EtOH.

The control experiments show that reaction does not proceed neither in the absence of light nor photocatalyst. Since, oxidation of thioanisole and triphenylphosphine is considered to proceed either through singlet oxygen or electron transfer followed by formation of radicals, [13–15] we have performed two additional sets of photooxidation experiments with **Bf-A3** in the presence of TEMPO (1.5 eqv. with respect to substrate) as the radical inhibitor and DABCO (1.5 eqv.) as the singlet oxygen scavenger. We have found that oxidations were supressed in the presence of both DABCO and TEMPO indicating that the process might be mediated by $^1O_2$ and $O_2^{\cdot -}$ radical generated in the reaction.

The formation of small amounts of byproduct (2-5%), namely (*E*)-3-formylacrylic acid, was sporadically observed as a result of oxidation of FA. It can be rationalized by partial conformational isomerization of intermediately formed carbanion, which further decompose to (*E*)-3-formylacrylate, whereas more abundant (Z) conformer condensate to 5-oxyfuran-2(5H)-one (Scheme S1).

Table S2. Supplementary data for the results of FA photooxidation reactions (Table S2). Catalyst molar percentage was fixed at 0.05% in all presented experiments. PA – pure for analysis grade.

Entry	PS	solvent	time / h	conversion / %	TOF / h^{-1}
1	**Bf-A3**	$CHCl_3$(PA)	1	20	410
2		$CHCl_3$(PA)+2EtOH	1	49	940
3		$CHCl_3$(HPLC)	1	12	240
4		$CHCl_3$(HPLC)+2EtOH	1	48	920
5		MeCN	0.5	26	1040
6		MeCN	1	52	1040
7		MeCN + 10ul MeOH	0.5	24	960
8		MeCN + 10ul MeOH	1	49	970
9	**Bf-C**	$CHCl_3$	1.5	8	100
10	**TPP**	$CHCl_3$	1.5	18	240
11		DCM	1.5	33	450

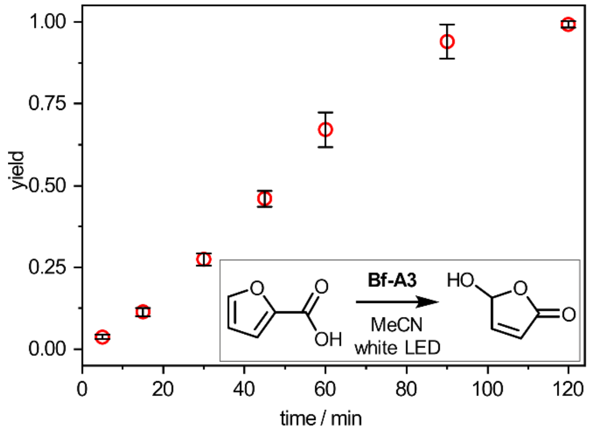

Figure S8. Averaged results of five independent FA photocatalytic oxidations with 0.05%mol **Bf-A3** in MeCN (26 W white LED, 25°C).

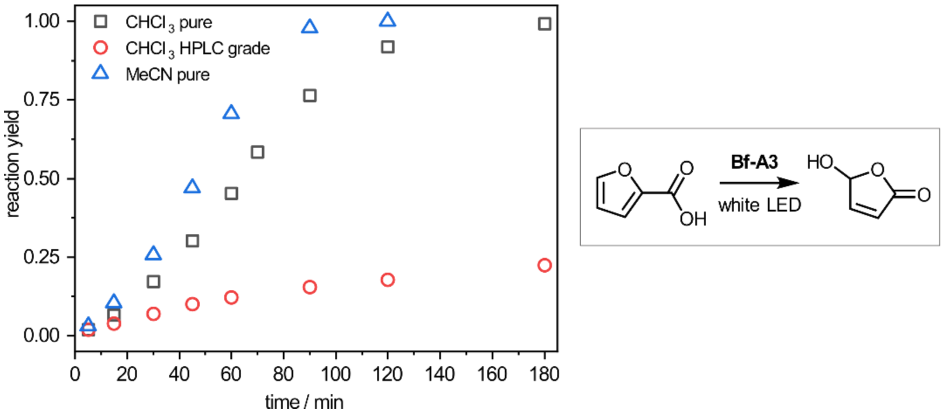

Figure S9. Results of FA photocatalytic oxidation with 0.05%mol **Bf-A3** in different solvents (26 W 395 nm LED, 25°C).

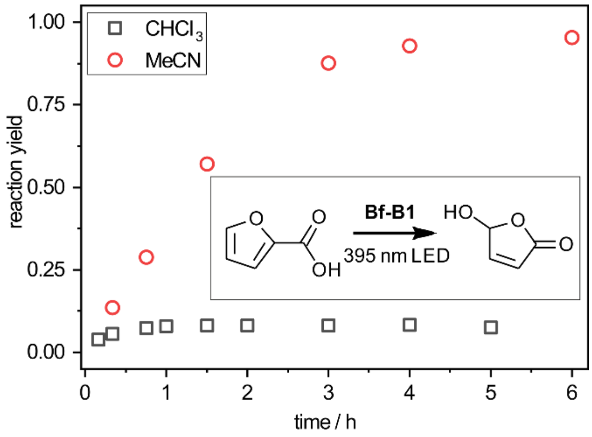

Figure S10. Photocatalytic oxidation of FA with 0.05%mol **Bf-B1** in MeCN and $CHCl_3$ (26 W 395 nm LED, 25°C).

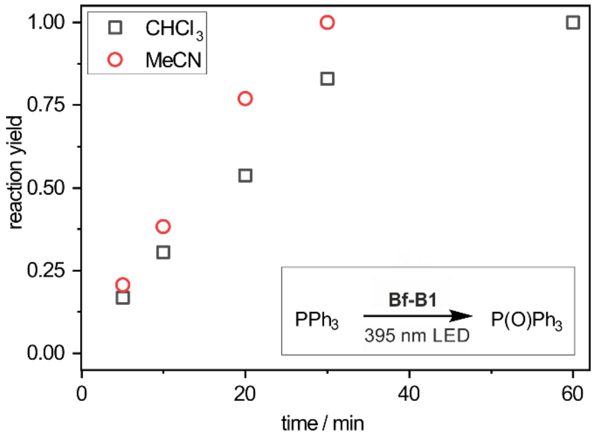

Figure S11. Photocatalytic oxidation of triphenylphosphine with 0.05%mol **Bf-B1** in $CHCl_3$ and MeCN (26 W 395 nm LED, 25°C).

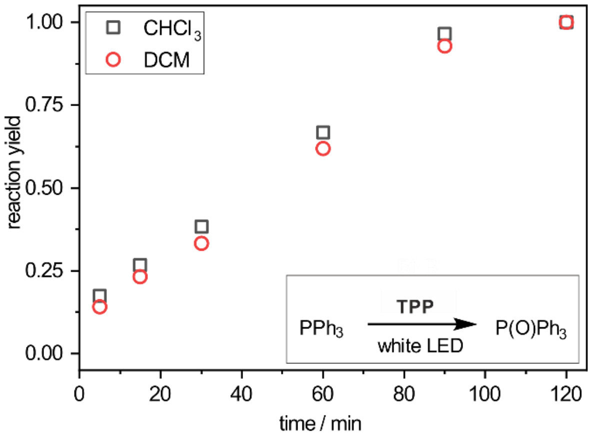

Figure S12. Photocatalytic oxidation of triphenylphosphine with 0.05% mol **TPP** in $CHCl_3$ and MeCN (26 W white LED, 25°C).

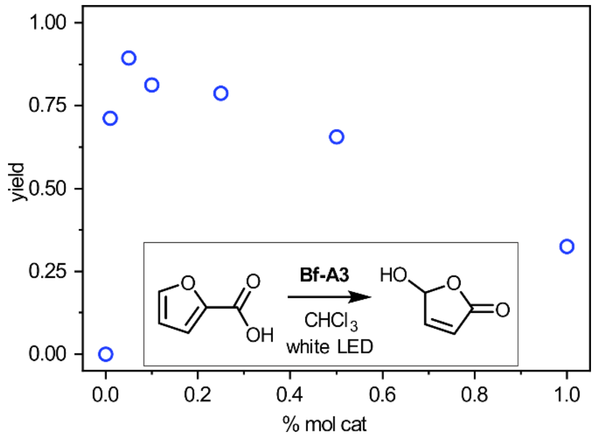

Figure S13. Photocatalytic oxidation of FA with **Bf-A3** as a function of concentration (0.00; 0.01; 0.05; 0.10; 0.25; 0.50; 1.00) in $CHCl_3$ (26 W white LED, 25°C). Reaction time: 1.5 h.

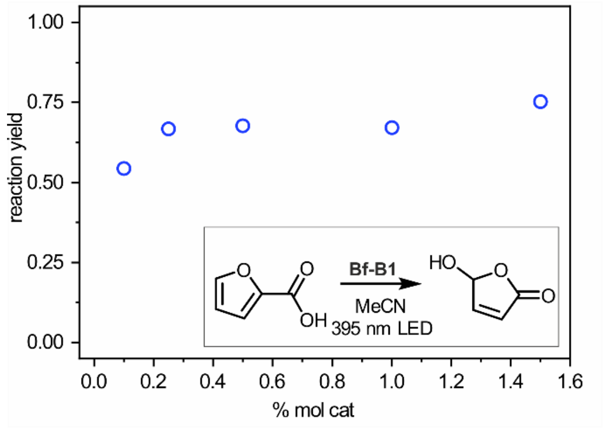

Figure S14. Photocatalytic oxidation of FA with **Bf-B1** as a function of concentration (0.05; 0.10; 0.25; 0.50; 1.00; 1.50) in MeCN (26 W 395 nm LED, 25°C). Reaction time: 1.5 h.

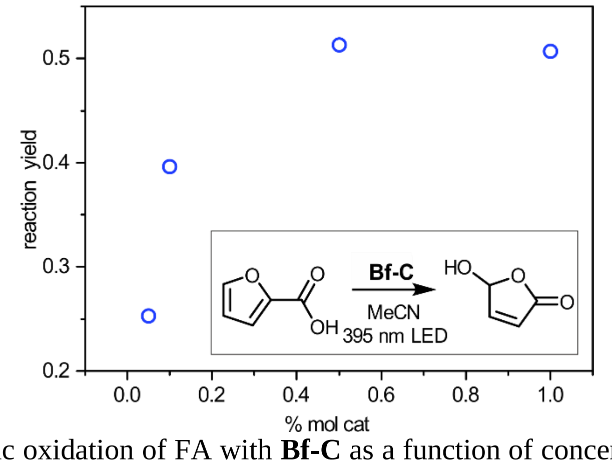

Figure S15. Photocatalytic oxidation of FA with **Bf-C** as a function of concentration (0.05; 0.10; 0.50, 1.00) in MeCN (26 W 395 nm LED, 25°C). Reaction time: 1.5 h.

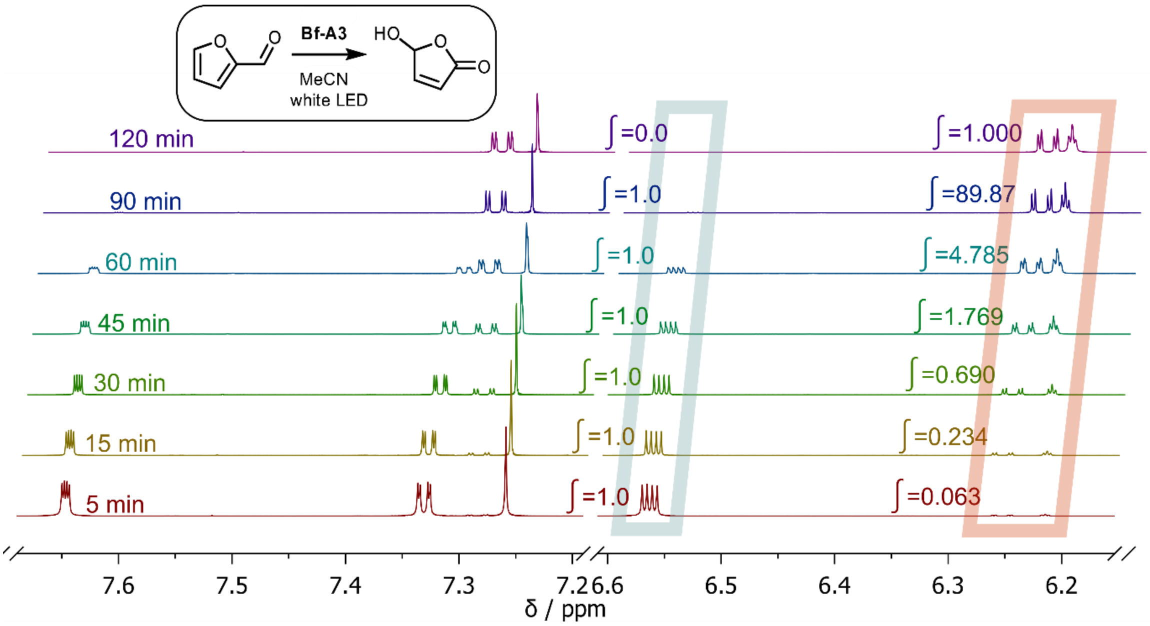

Figure S16. Analysis of ^{1}H NMR spectra (400 MHz, $CDCl_3$, 298 K) for photocatalytic oxidation of FA with 0.05%mol **Bf-A3** (MeCN, 26 W white LED, 25°C). Integrals used for determination of reaction conversion are marked.

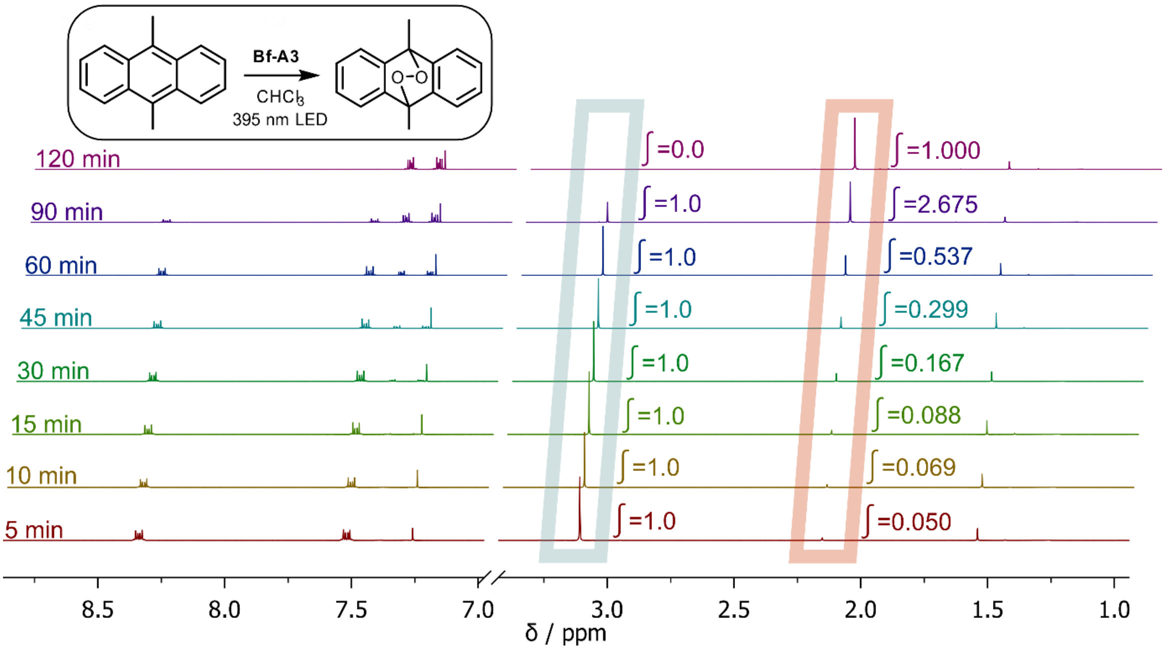

Figure S17. Analysis of ^{1}H NMR spectra (400 MHz, $CDCl_3$, 298 K) for photocatalytic oxidation of DMA with 0.05%mol **Bf-A3** (MeCN, 26 W white LED, 25°C). Integrals used for determination of reaction conversion are marked.

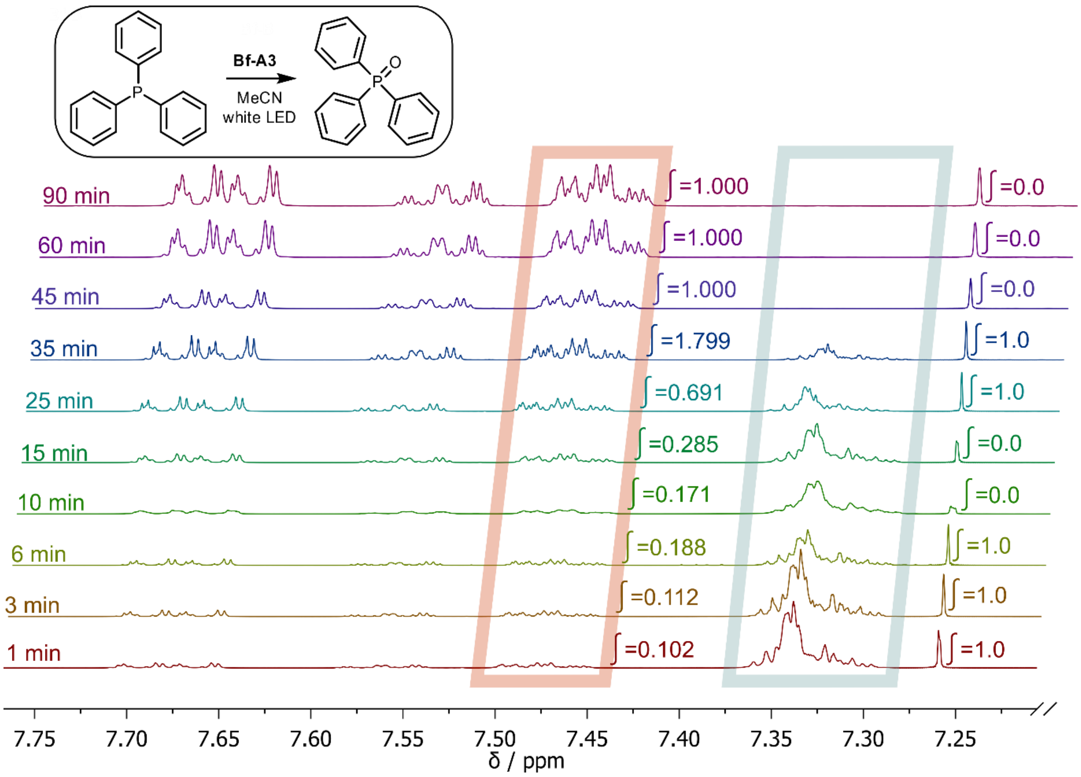

Figure S18. Analysis of ^{1}H NMR spectra (400 MHz, $CDCl_3$, 298 K) for photocatalytic oxidation of PPh_3 with 0.05%mol **Bf-A3** (MeCN, 26 W white LED, 25°C). Integrals used for determination of reaction conversion are marked.

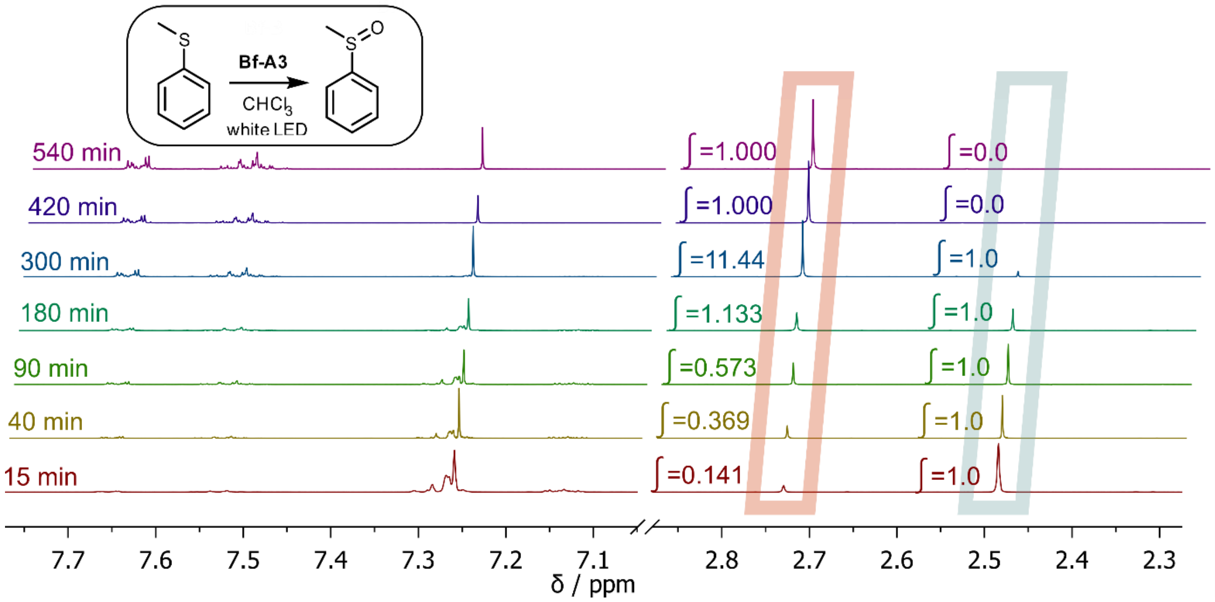

Figure S19. Analysis of ^{1}H NMR spectra (400 MHz, $CDCl_3$, 298 K) for photocatalytic oxidation of PhSMe with 0.05%mol **Bf-A3** (MeOH/MeCN 2:1, 26 W white LED, 25°C). Integrals used for determination of reaction conversion are marked.

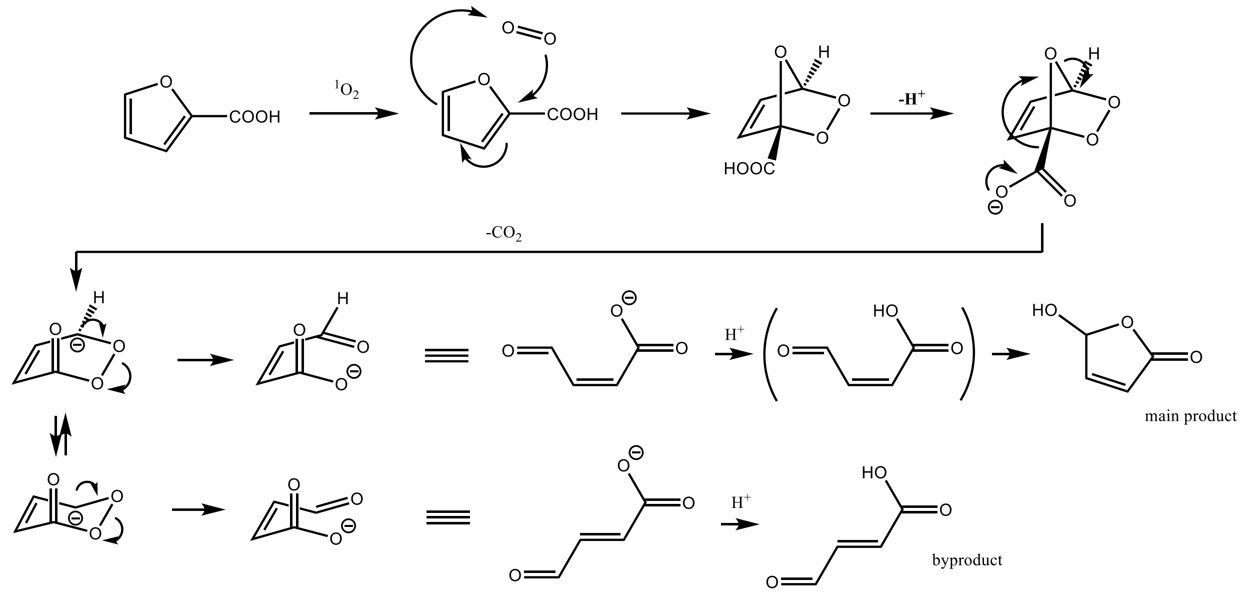

Scheme S1. Two possible reaction pathways for the oxidation of FA with singlet oxygen.

5. Photocatalytic stability

Photocatalytic stability under reaction conditions for all analysed photosensitizers was determined by UV-Vis spectroscopy using Hitachi U-2800 spectrophotometer. Additionally, corresponding measurements were carried out without irradiation, on samples held in darkroom. Referential dyes, Rose Bengal (**RB**) and tetraphenylporphyrin (**TPP**), were measured in the same manner for comparison. Experimental conditions were retained from photocatalytic test reactions. The parallel experiments were performed without light to determine hydrolytic stability.

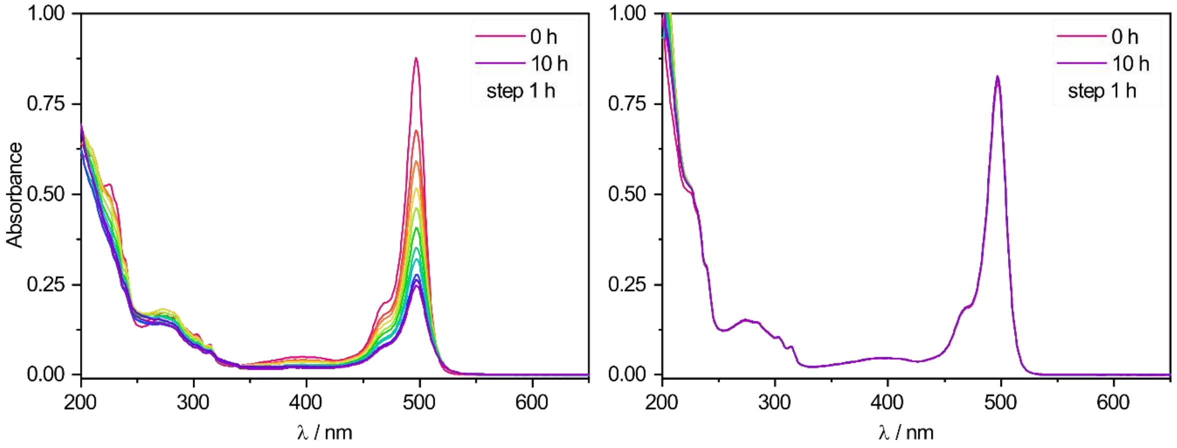

Figure S20. Overlay of absorption spectra of **Bf-A3** in MeCN upon irritation (left) and in the darkroom (right).

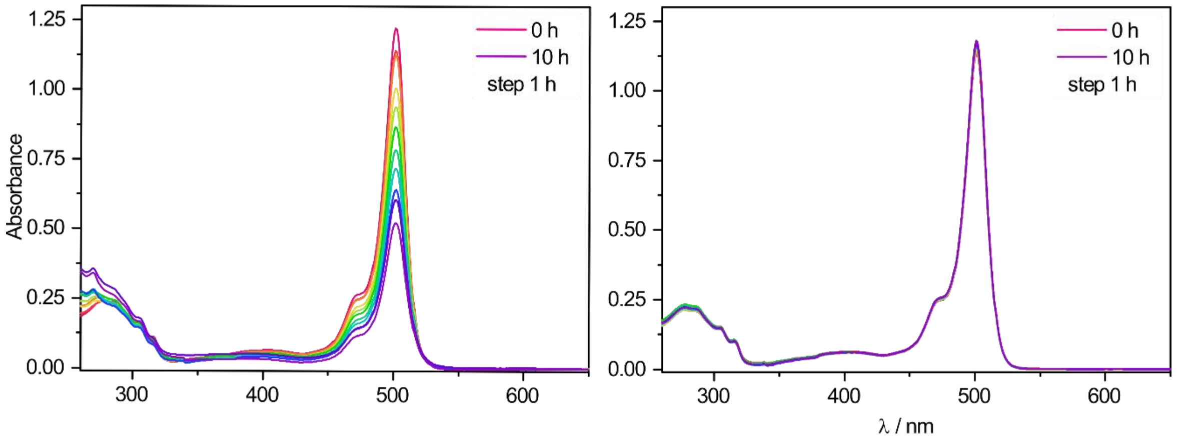

Figure S21. Overlay of absorption spectra of **Bf-A3** in $CHCl_3$ upon irradiation (left) and in the darkroom (right).

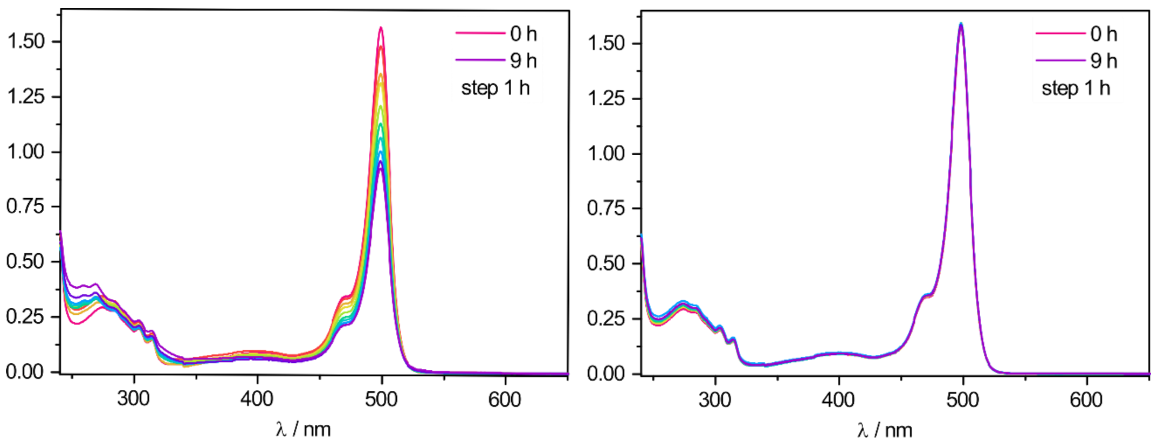

Figure S22. Overlay of absorption spectra of **Bf-A3** in MeOH:MeCN (2:1) upon irradiation (left) and in the darkroom (right).

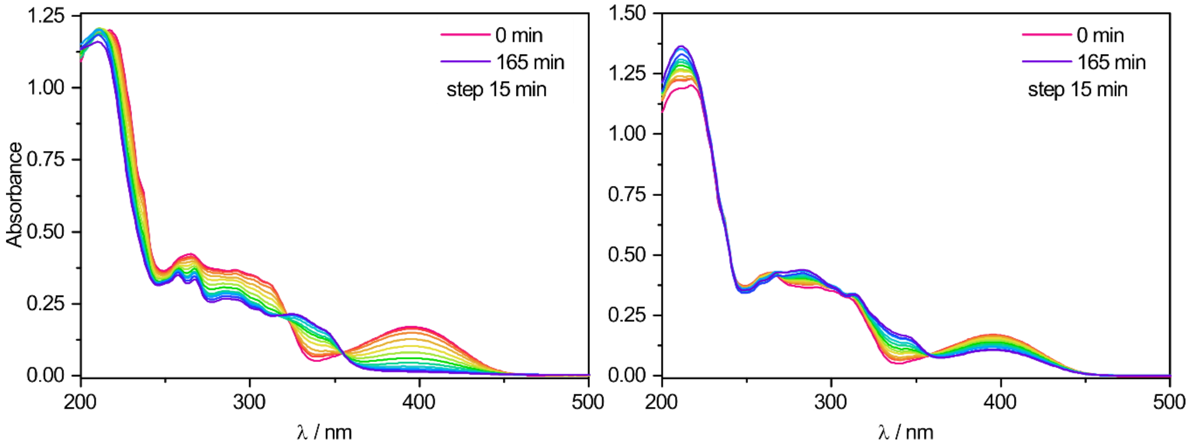

Figure S23. Overlay of absorption spectra of **Bf-B1** in MeCN upon irradiation (left) and in the darkroom (right).

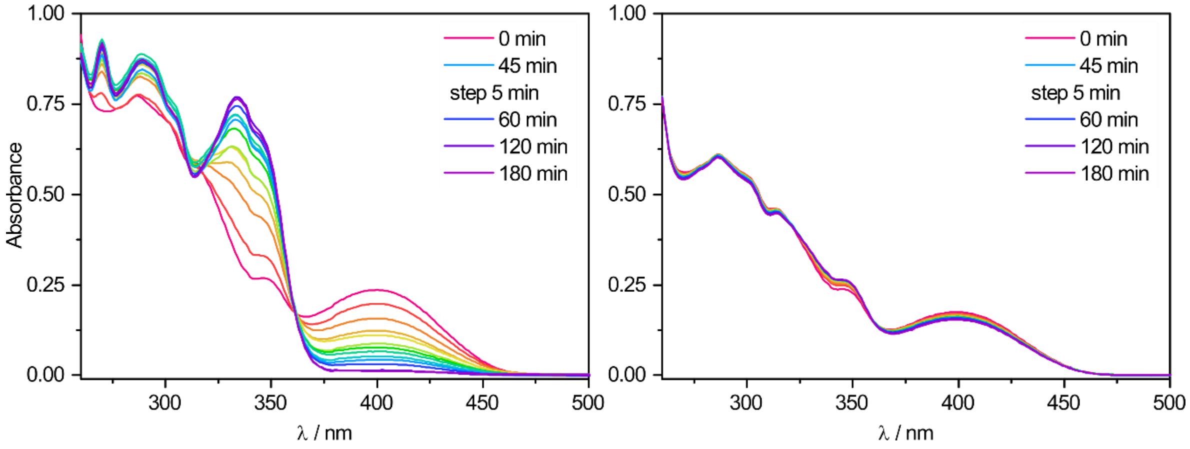

Figure S24. Overlay of absorption spectra of **Bf-B1** in $CHCl_3$ upon irradiation (left) and in the darkroom (right).

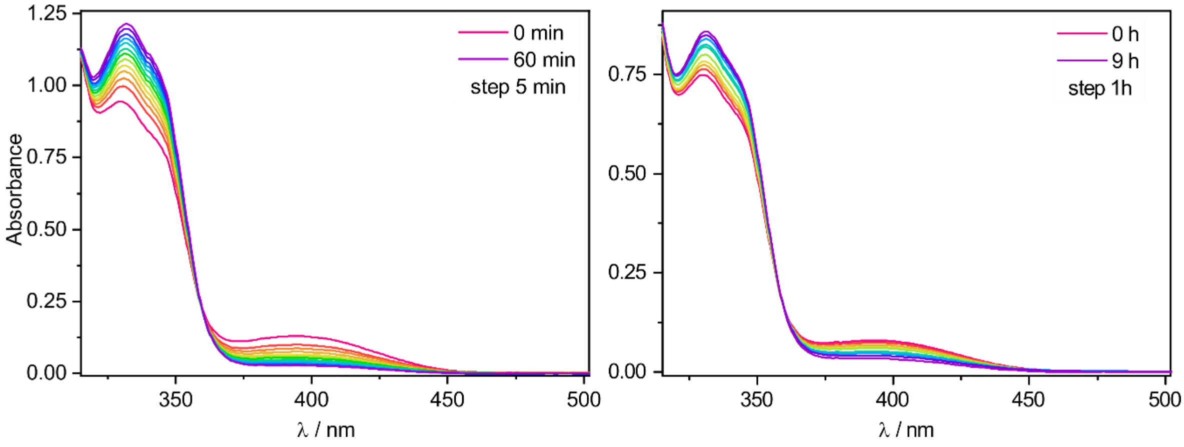

Figure S25. Overlay of absorption spectra of **Bf-B1** in MeOH:MeCN (2:1) upon irradiation (left) and in the darkroom (right).

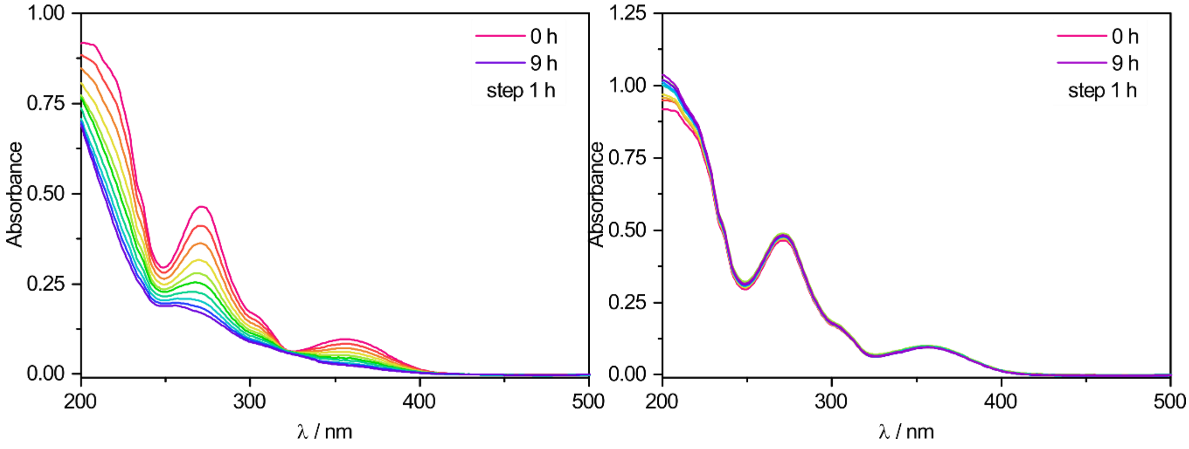

Figure S26. Overlay of absorption spectra of **Bf-C** in MeCN upon irradiation (left) and in the darkroom (right).

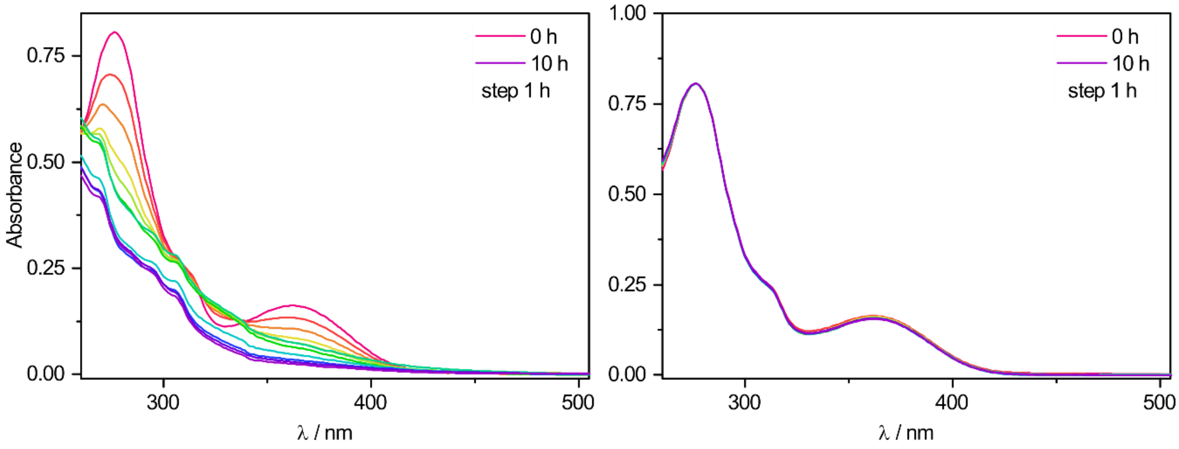

Figure S27. Overlay of absorption spectra of **Bf-C** in $CHCl_3$ upon irradiation (left) and in the darkroom (right).

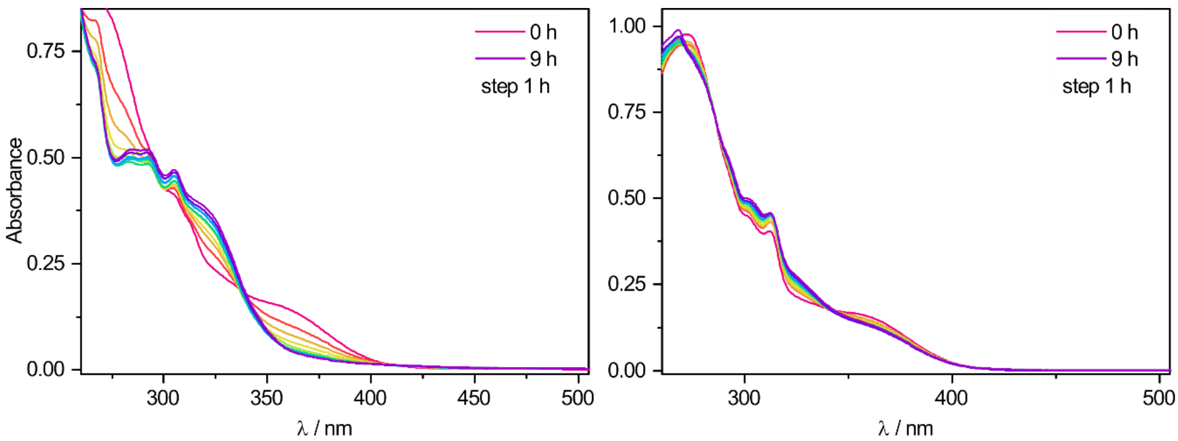

Figure S28. Overlay of absorption spectra of **Bf-C** in MeOH:MeCN (2:1) upon irradiation (left) and in the darkroom (right).

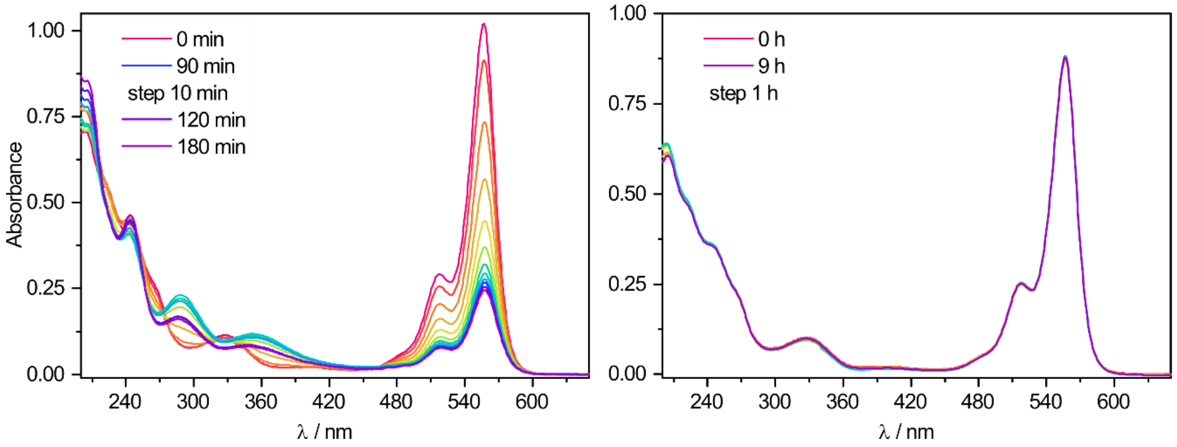

Figure S29. Overlay of absorption spectra of **RB** in MeCN upon irritation (left) and in the darkroom (right).

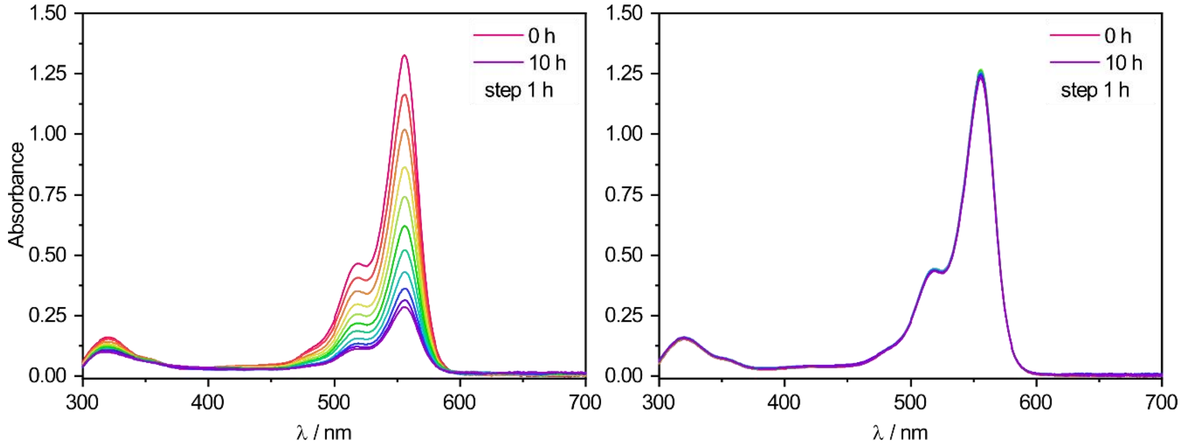

Figure S30. Overlay of absorption spectra of **RB** in MeOH upon irritation (left) and in the darkroom (right).

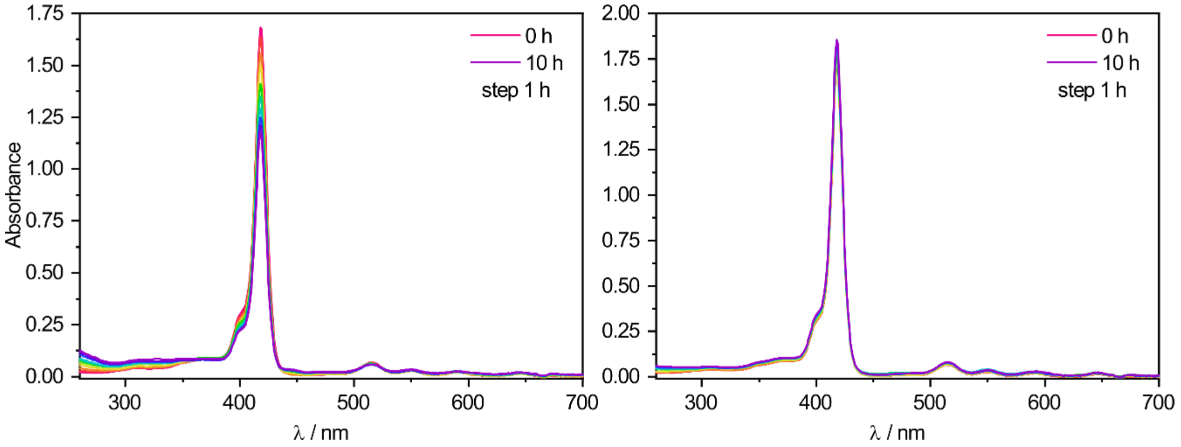

Figure S31. Overlay of absorption spectra of **TPP** in $CHCl_3$ upon irritation (left) and in the darkroom (right).

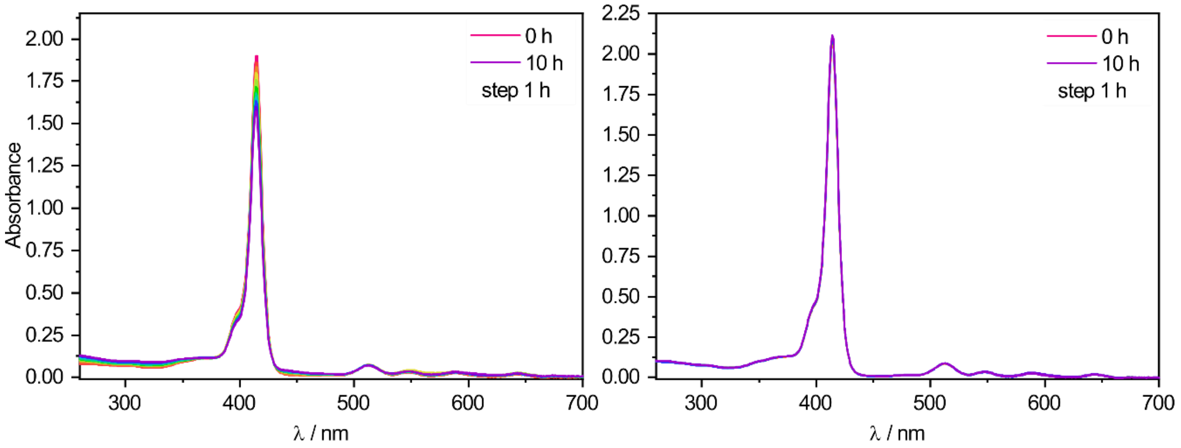

Figure S32. Overlay of absorption spectra of **TPP** in MeOH:MeCN (2:1) mixture upon irritation (left) and in the darkroom (right).

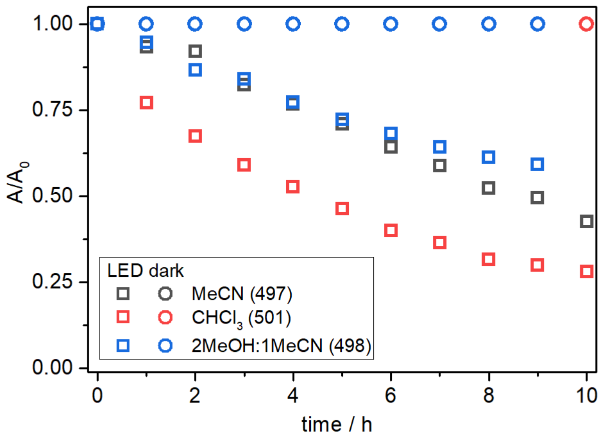

Figure S33. Drop in absorption intensity at the absorption maximum wavelength (given in brackets) recorded for **Bf-A3** solutions in MeCN, $CHCl_3$ and MeOH:MeCN (2:1) upon irradiation and stored in dark.

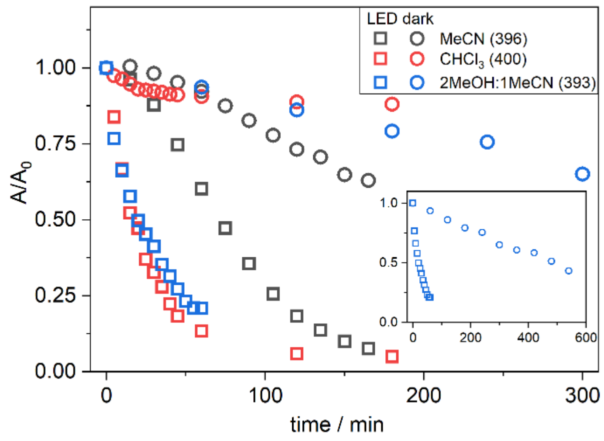

Figure S34. Drop in absorption intensity at the absorption maximum wavelength (given in brackets) recorded for **Bf-B1** solutions in MeCN, CHCl3 and MeOH:MeCN (2:1) upon irradiation and stored in dark.

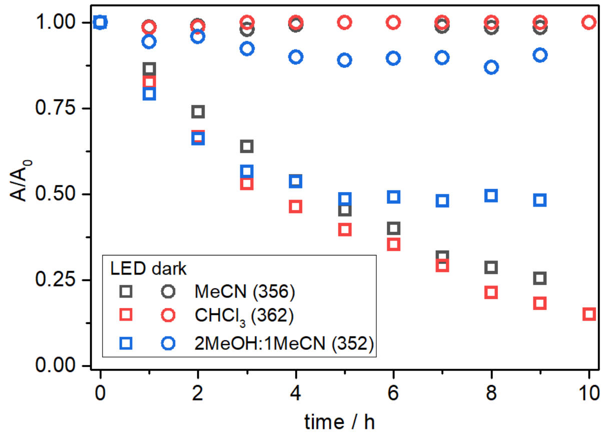

Figure S35. Drop in absorption intensity at the absorption maximum wavelength (given in brackets) recorded for **Bf-C** solutions in MeCN, $CHCl_3$ and MeOH:MeCN (2:1) upon irradiation and stored in dark.

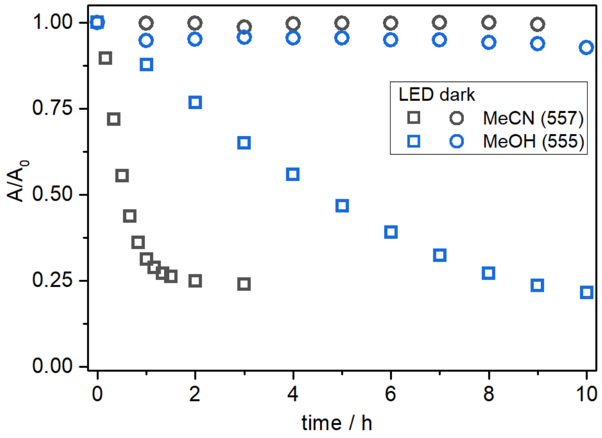

Figure S36. Drop in absorption intensity at the absorption maximum wavelength (given in brackets) recorded for **RB** solutions in MeCN and MeOH upon irradiation and stored in dark.

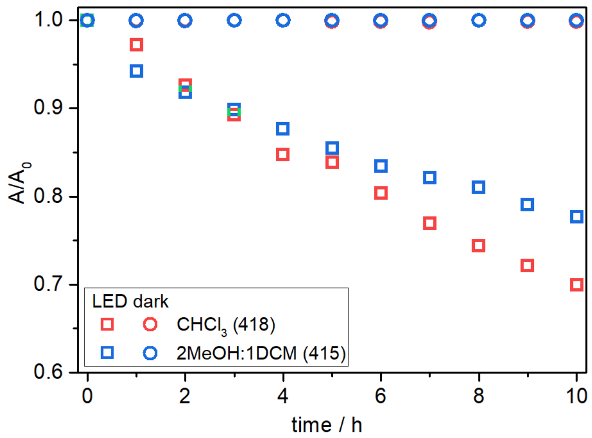

Figure S37. Drop in absorption intensity at the absorption maximum wavelength (given in brackets) recorded for **TPP** solutions in $CHCl_3$ and MeOH:DCM (2:1) upon irradiation and stored in dark.

6. Crystal structures

Single crystals suitable for single crystal diffraction measurements of **BPh$_2$-A1**, **BF$_2$-A1**, **Bf-A2** and **Bf-A1** were obtained by slow solvent evaporation from corresponding $CHCl_3$ solutions. X-ray diffraction data were collected on a SuperNova diffractometer equipped with Atlas detector using Cu-Kα radiation (λ = 1.5418 Å). Data reduction and analysis were carried out with the CrysAlisPro program.[16] All structures were solved by intrinsic phasing using SHELXT[17] and refined using SHELXL-2014[18] with Olex2 suite.[19] All non-hydrogen atoms were refined anisotropically. Selected crystal data are summarized in Table S3. The crystal structure of **BPh$_2$-A1** is similar to already published structure of related BODIPY system, namely 2,6-Diethyl-4,4-difluoro-1,3,5,7-tetramethyl-8-(4-methylphenyl)-4-bora-3a,4a-diaza-s-indacene. The latter system comprises (4-methylphenyl) substituent at *meso* position of BODIPY frame.[20] The geometrical parameters of both molecules are in general very similar, however the crystal packing is different (**BPh$_2$-A1** crystalizes in monoclinic $P2_1/c$ space group, while its methyl-substituted derivative in triclinic P-1 space group with two molecules in the asymmetric part of the unit cell). Crystallographic Information Files (CIFs) have been deposited with the Cambridge Crystallographic Data Centre as supplementary publications no. 2068807, 2068808, 2068809 and 2068810. The structures of remaining complexes were reported elsewhere.[12,21]

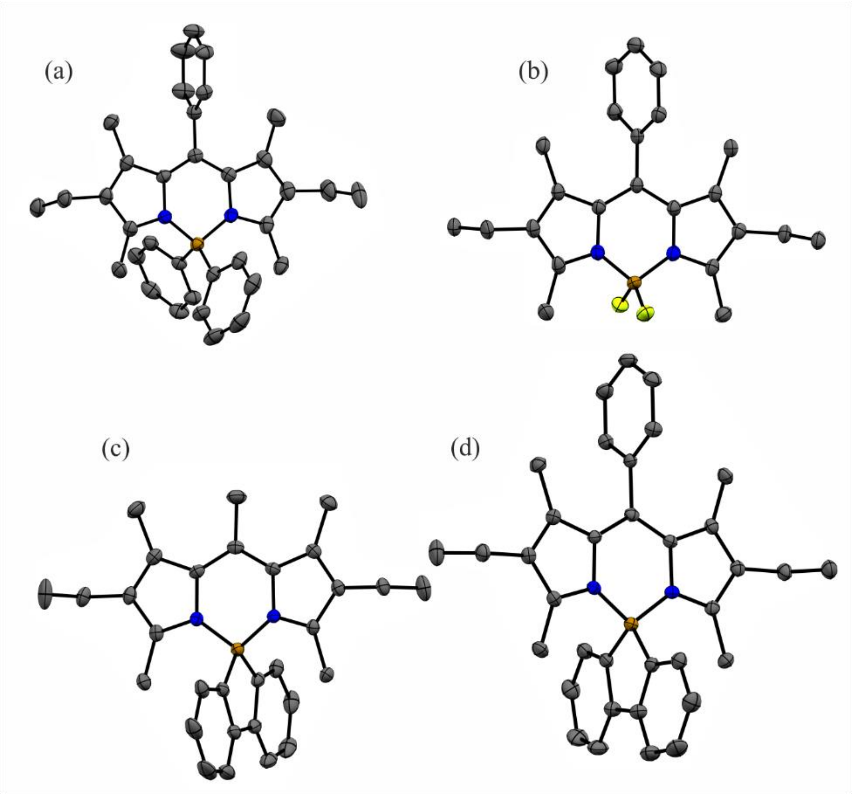

Figure S38. Molecular structures of (a) **BPh$_2$-A1**, (b) **BF$_2$-A1** (c) **Bf-A2** and (d) **Bf-A1**. Thermal ellipsoids are depicted with 50% of probability. Hydrogen atoms were omitted for clarity.

Table S3. Crystal and refinement data.

Identification code	**Bf-A1**	**Bf-A2**	**BF_2-A1**	**BPh_2-A1**
Empirical formula	$C_{35}H_{35}BN_2$	$C_{30}H_{33}BN_2$	$C_{23}H_{27}BF_2N_2$	$C_{35}H_{37}BN_2$
Formula weight	494.46	432.39	380.27	496.47
Temperature/K	100(2)	100(2)	100.01(10)	100.00(10)
Crystal system	monoclinic	monoclinic	monoclinic	monoclinic
Space group	$P2_1/n$	$P2_1/c$	$P2_1/c$	$P2_1/c$
a / Å	8.2782(2)	8.13890(10)	7.84741(16)	7.8980(2)
b / Å	18.7847(2)	19.5267(2)	11.5223(2)	20.7713(5)
c / Å	17.8700(4)	14.8258(2)	21.9627(6)	17.2217(4)
α / °	90	90	90	90
β / °	100.307(2)	90.2670(10)	93.660(2)	99.833(2)
γ / °	90	90	90	90
Volume / Å^3	2734.01(10)	2356.18(5)	1981.83(7)	2783.75(12)
Z	4	4	4	4
ρ_{calc} / gcm^{-3}	1.201	1.219	1.275	1.185
μ / mm^{-1}	0.520	0.527	0.700	0.511
F(000)	1056.0	928.0	808.0	1064.0
Crystal size/mm^3	0.143 × 0.1112 × 0.0743	0.02 × 0.01 × 0.01	0.1863 × 0.0904 × 0.0812	0.121 × 0.066 × 0.051
2Θ range for data collection / °	6.886 to 147.692	7.486 to 148.964	8.068 to 147.966	6.726 to 147.704
Index ranges	$-10 \le h \le 8$, $-23 \le k \le 23$, $-22 \le l \le 22$	$-10 \le h \le 10$, $-24 \le k \le 24$, $-17 \le l \le 18$	$-9 \le h \le 9$, $-14 \le k \le 14$, $-23 \le l \le 26$	$-9 \le h \le 5$, $-23 \le k \le 25$, $-21 \le l \le 20$
Reflections collected	42947	19470	24484	15130
Independent reflections / R_{int} / R_{sigma}	5498 / 0.0279 / 0.0134	4546 / 0.0239 / 0.0165	3987 / 0.0275 / 0.0156	5519 / 0.0402 / 0.0468
Data / restraints / parameters	5498 / 0 / 349	4546 / 0 / 305	3987 / 0 / 259	5519 / 0 / 350
Goodness-of-fit on F^2	1.028	1.052	1.135	1.020
Final R indexes [I>=2σ (I)]	R_1 = 0.0359, wR_2 = 0.0917	R_1 = 0.0486, wR_2 = 0.1256	R_1 = 0.0653, wR_2 = 0.1931	R_1 = 0.0441, wR_2 = 0.1048
Final R indexes [all data]	R_1 = 0.0401, wR_2 = 0.0956	R_1 = 0.0524, wR_2 = 0.1295	R_1 = 0.0697, wR_2 = 0.1972	R_1 = 0.0659, wR_2 = 0.1163
Largest diff. peak/hole / e Å^{-3}	0.30/-0.20	0.49/-0.22	0.63/-0.35	0.29/-0.20

7. Theoretical calculations

Ab initio calculations were performed using *Gaussian16* programme package.[22] In the first step the molecules were optimised in their ground states using B3LYP (DFT)[23,24] method with 6-31+G(d)[25] basis set. Starting geometries were adopted from crystal structures. Structures **BPh2-A1**, **BF2-A1**, **Bf-A1** and **Bf-A2** were measured by us in the framework of this work, **Bf-A3**,[21] **Bf-B1**, **Bf-B2**, **Bf-B3**, **Bf-C**, **Bf-D**, **Bf-E**[12] are already published. Crystal structures of **BPh2-A2** and **BF2-A2** are unknown, thus they were constructed in *GausView* 6.0 program based on closely related **BPh2-A1** and **BF2-A1** analogues. After geometry optimization, the vibrational frequencies were calculated and the results showed that optimized structures are stable geometric structures (no imaginary frequencies). Calculated molecular orbitals for representative complexes (**BF2-A1**, **BPh2-A1**, **Bf-A1**, **Bf-A3, Bf-B1** and **Bf-C**) in their ground and excited states are presented on Figures S39-S44. They were visualized with *Avogadro* programme.[26] In the next step, excited singlet and triplet state geometries were obtained with TD-DFT methods. DFT and TD-DFT calculations were performed in the presence of the solvent (MeCN) with the polarizable continuum model (PCM).[27] The energy values of two lowest energy excited singlet and five lowest energy triplet states with respect to ground state energy are provided in Table S4.

The calculations show that molecular geometries of singlet and triplet excited states are generally preserved from corresponding ground states (Figure S45). The estimated values for emission maxima were compared to experimental values (Table S5). Since the calculated values are typically underestimated with respect to experimental values by 10-60 nm, it is expected that in case of borafluorene complexes the second lowest energy singlet excited state would correspond to experimentally observed transition, while excitation to lowest energy singlet excited state would be inefficient. This statement is supported by low oscillator strength value for CT transition, and much higher oscillator strength value for LE transition.

In order to determine the nature of singlet and triplet excited states Natural Transition Orbitals[28] were calculated. The NTO calculations were performed for **BF2-A1**, **BPh2-A1** and **Bf-A1** series in order to elucidate the effect of boron atom substitution, and for **Bf-A3**, **Bf-B1** and **Bf-C** complexes to show differences between various ligands (Figure S48-S53). The energy diagrams are presented on Figure S46 and S47. In case of Bf complexes ^{1}CT character of the lowest energy singlet excited state is confirmed by the location of highest occupied (HONTO) and lowest unoccupied natural transient orbitals (LUNTO). The location of HONTO and LUNTO for second lowest energy singlet excited state indicates that it possesses local character (^{1}LE-Lig). Spin density isosurfaces generated for all considered molecules in their optimised lowest energy triplet states (Figure S54, S55) indicate that the lowest energy triplet state is located on ligand (^{3}LE-Lig). The energy of this state is similar comparing the corresponding BF_2, BPh_2 and Bf complexes from A1 and A2 series. This suggests that boron substitution has marginal influence on the energy of lowest energy triplet state. In turn, the observed differences between Bf complexes (**Bf-BODIPYs**, **Bf-B1**, **Bf-B2**, **Bf-B3**, **Bf-C**, **Bf-D**, **Bf-E**) originate from different ligand electronic features.

Table S4. Calculated energy values of two lowest energy singlet (S_n) and five lowest energy triplet (T_n) states for all studied complexes. The values are given with respect to the energy of ground state in its optimised geometry. The nature of excited states in **BF_2-A1**, **BPh_2-A1**, **Bf-A1**, **Bf-A3**, **Bf-B1** and **Bf-C** were evaluated with NTO analysis.

Complex	State	*E* / eV	Complex	State	*E* / eV
**BF_2-A1**	S_1	2.50	**BF_2-A2**	S_1	2.54
	S_2	3.23		S_2	3.32
	T_1	3.43		T_1	1.44
	T_2	1.36		T_2	2.77
	T_3	2.62		T_3	2.89
	T_4	2.70		T_4	3.28
	T_5	2.85		T_5	4.44
**BPh_2-A1**	S_1	2.58	**BPh_2-A2**	S_1	2.54
	S_2	3.03		S_2	3.22
	T_1	3.17		T_1	1.50
	T_2	1.48		T_2	2.67
	T_3	2.51		T_3	2.83
	T_4	2.65		T_4	3.22
	T_5	2.74		T_5	3.64
**Bf-A1**	S_1	2.45	**Bf-A2**	S_1	2.53
	S_2	2.60		S_2	2.61
	T_1	1.47		T_1	1.53
	T_2	2.42		T_2	2.49
	T_3	2.61		T_3	2.62
	T_4	2.62		T_4	2.70
	T_5	2.81		T_5	2.88
**Bf-A3**	S_1	2.41	**Bf-B1**	S_1	2.54
	S_2	2.68		S_2	2.77
	T_1	1.51		T_1	2.11
	T_2	2.44		T_2	2.78
	T_3	2.66		T_3	3.01
	T_4	3.09		T_4	3.15
	T_5	3.11		T_5	3.37
**Bf-B2**	S_1	2.71	**Bf-B3**	S_1	2.91
	S_2	3.08		S_2	3.26
	T_1	2.28		T_1	2.42
	T_2	2.90		T_2	3.18
	T_3	3.16		T_3	3.24
	T_4	3.18		T_4	3.35
	T_5	3.72		T_5	3.50
**Bf-C**	S_1	2.78	**Bf-D**	S_1	2.38
	S_2	2.99		S_2	2.68
	T_1	2.38		T_1	1.95
	T_2	3.07		T_2	2.60
	T_3	3.24		T_3	2.84
	T_4	3.36		T_4	3.30
	T_5	3.44		T_5	3.47
**Bf-E**	S_1	2.64			
	S_2	2.90			
	T_1	1.82			
	T_2	2.93			
	T_3	3.28			
	T_4	3.67			
	T_5	3.77			

Table S5. Calculated and experimental values of λ_{emi} maxima for all studied complexes. Oscillator strength are given in brackets.

Complex	$\lambda_{emi/calc}$ / nm	$\lambda_{emi/exp}$ / nm
**BF_2-A1**	506.44 (0.7281)<br>391.06 (0.1579)	539
**BPh_2-A1**	492.88 (0.7659)<br>404.42 (0.0857)	530
**Bf-A1**	555.34 (0.0516)<br>480.01 (0.7815)	533
**BF2-A2**	503.08 (0.7393)<br>381.91 (0.1491)	538
**BPh_2-A2**	515.02 (0.6975)<br>401.04 (0.0769)	549
**Bf-A2**	556.87 (0.0055)<br>475.03 (0.2532)	539
**Bf-A3**	571.31 (0.0053)<br>469.28 (0.7996)	507
**Bf-B1**	552.46 (0.0763)<br>475.16 (0.1893)	497
**Bf-B2**	523.39 (0.0373)<br>452.40 (0.2686)	461
**Bf-B3**	487.21 (0.0356)<br>428.92 (0.1770)	443
**Bf-C**	513.47 (0.0384)<br>446.26 (0.0674)	482
**Bf-D**	620.02 (0.0126)<br>495.29 (0.2180)	559
**Bf-E**	526.53 (0.0991)<br>472.41 (0.2842)	513

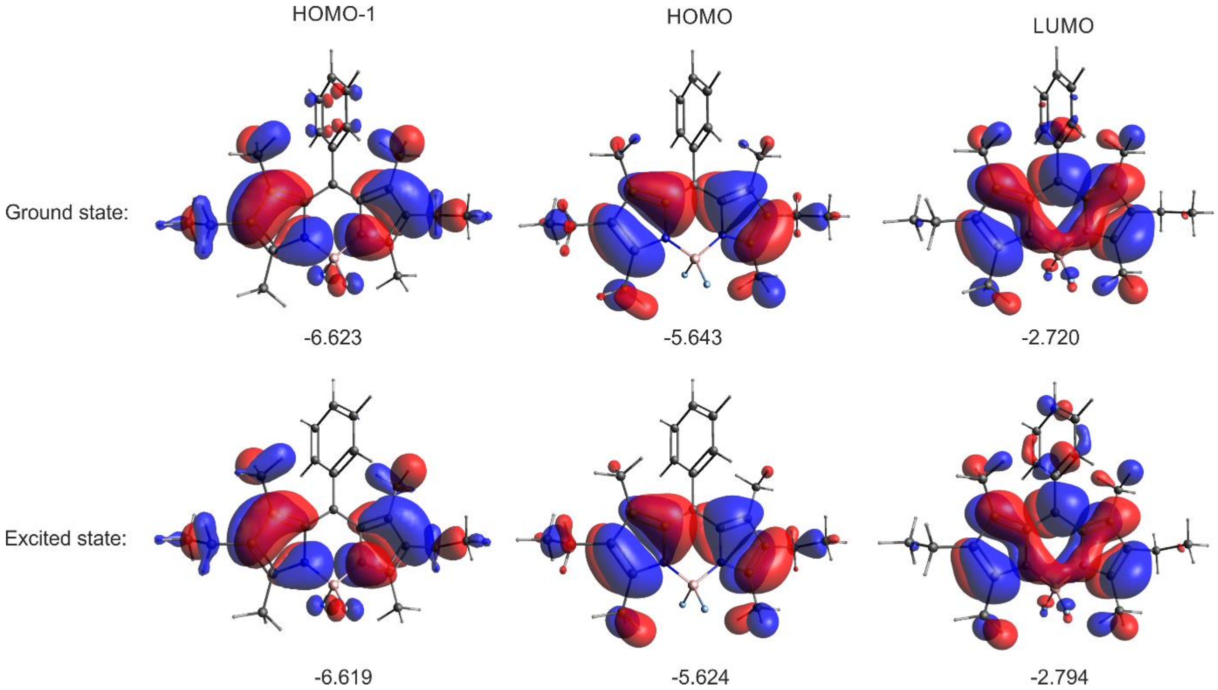

Figure S39. Frontier molecular orbitals in **BF_2-A3** in ground and excited states; *iso* = 0.2.

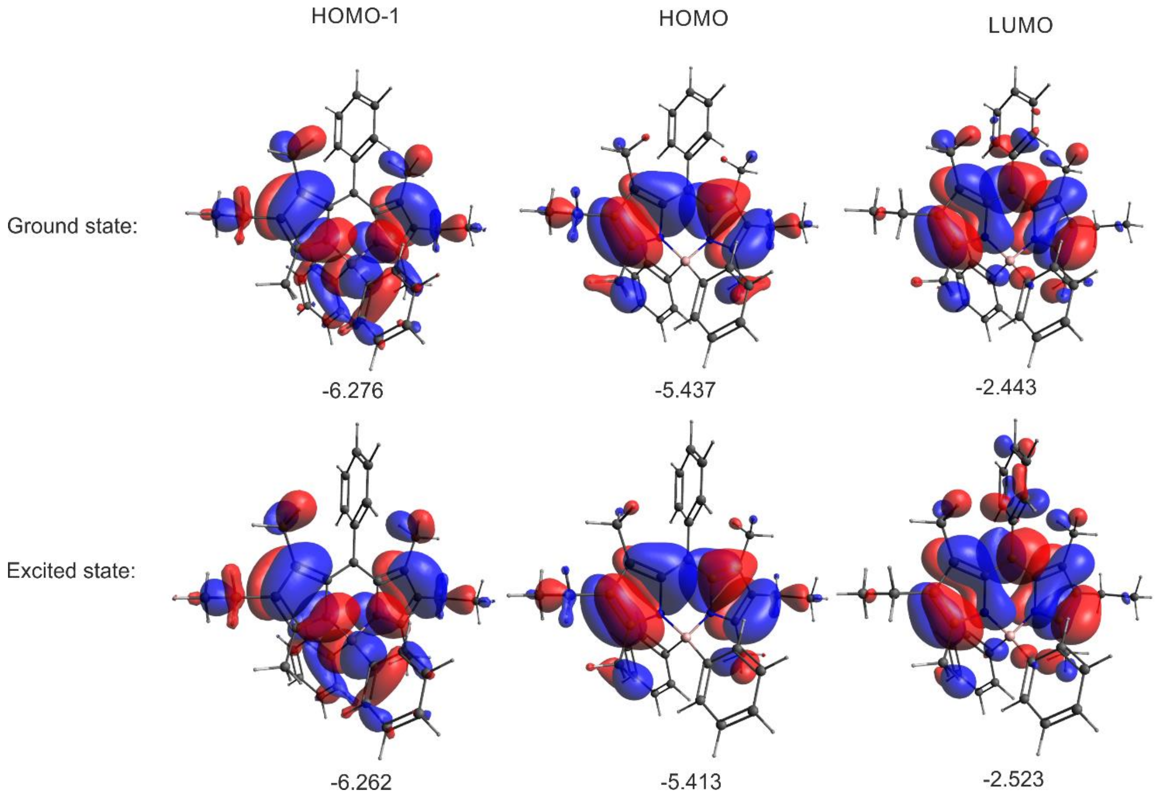

Figure S40. Frontier molecular orbitals in **BPh$_2$-A3** in ground and excited states; *iso* = 0.2.

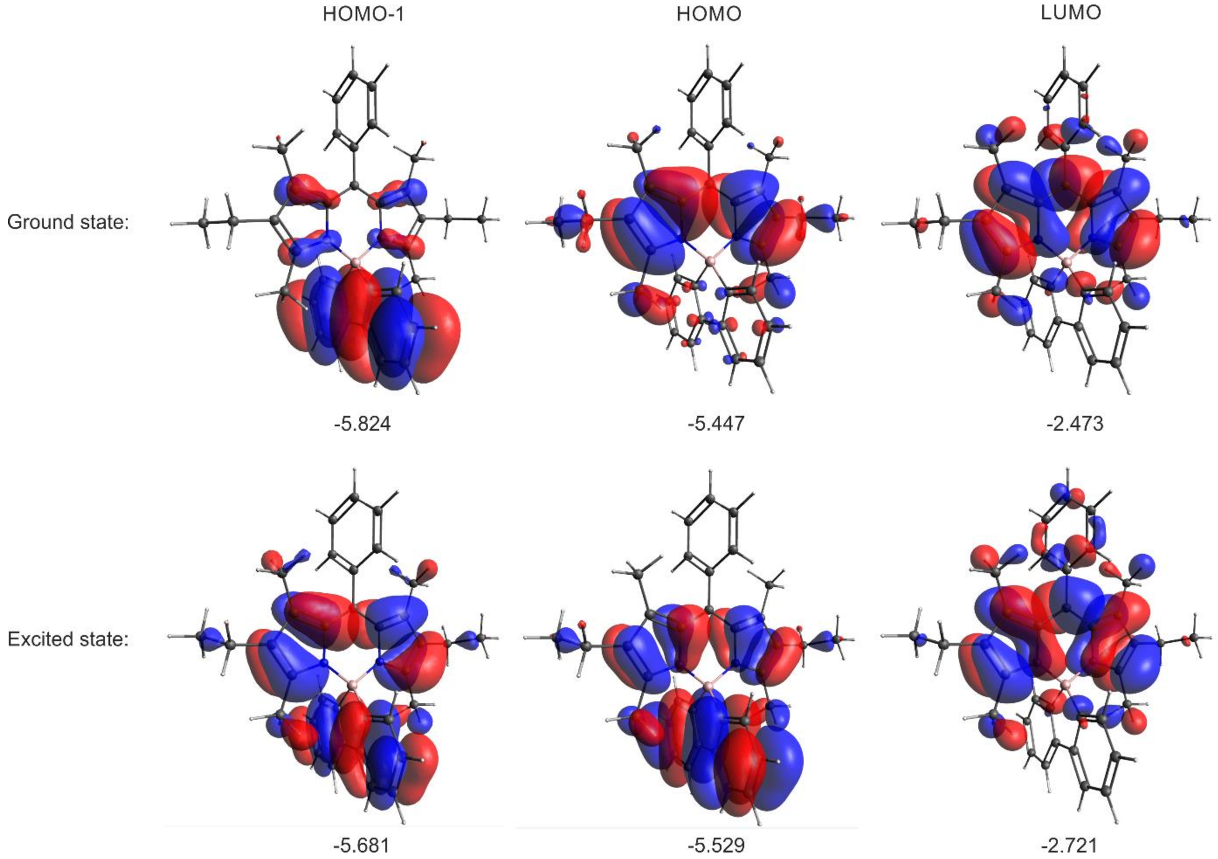

Figure S41. Frontier molecular orbitals in **Bf-A1** in ground and excited states; *iso* = 0.2.

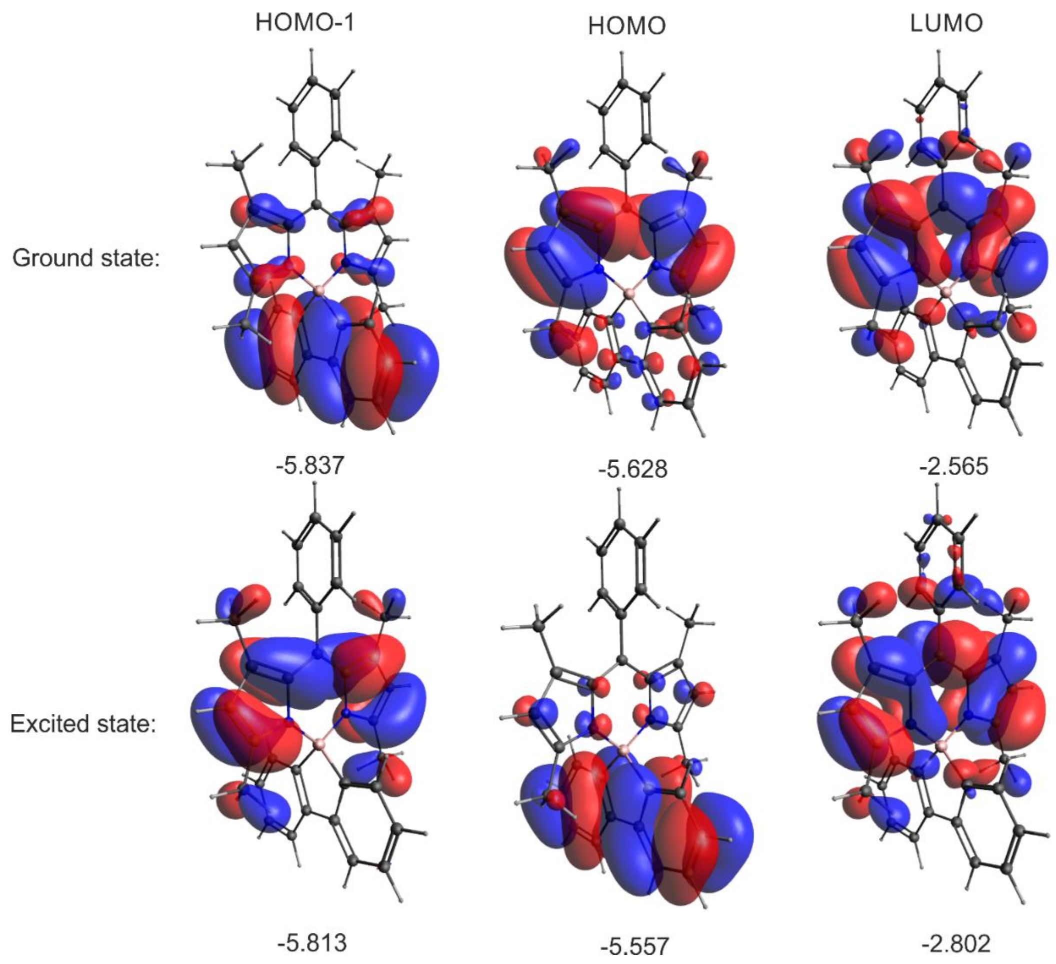

Figure S42. Frontier molecular orbitals in **Bf-A3** in ground and excited states; *iso* = 0.2.

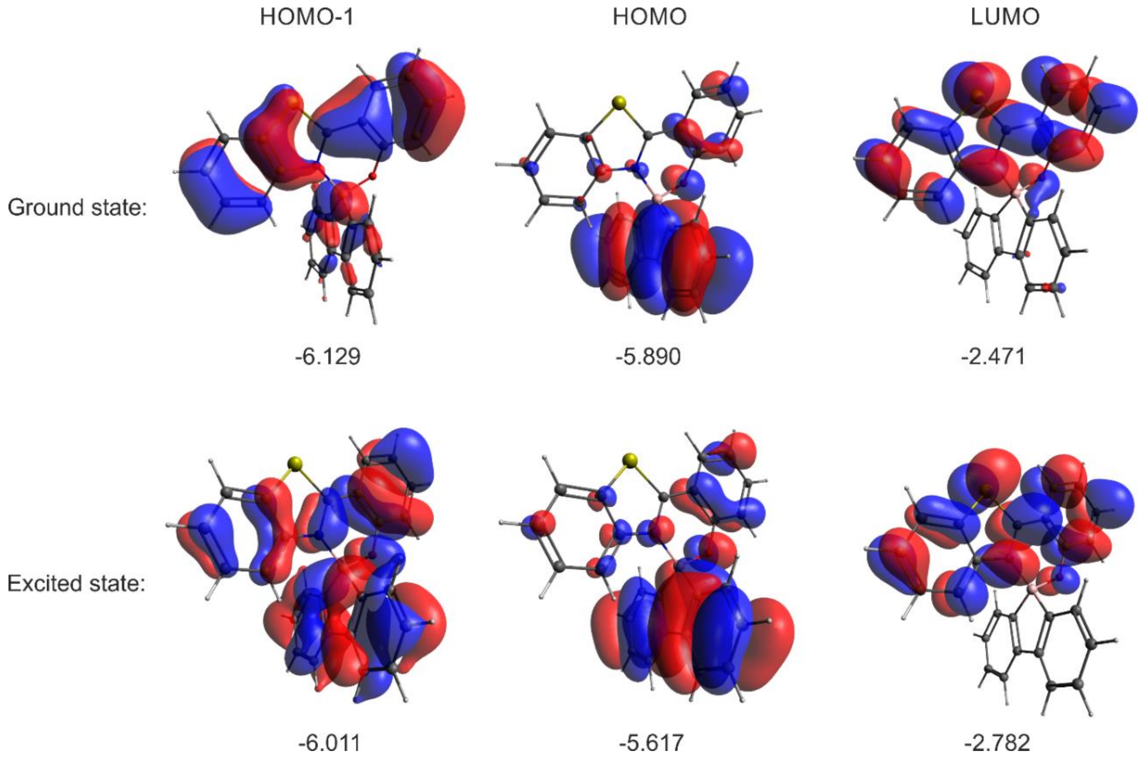

Figure S43. Frontier orbitals in **Bf-B1** in ground and excited states; *iso* = 0.2.

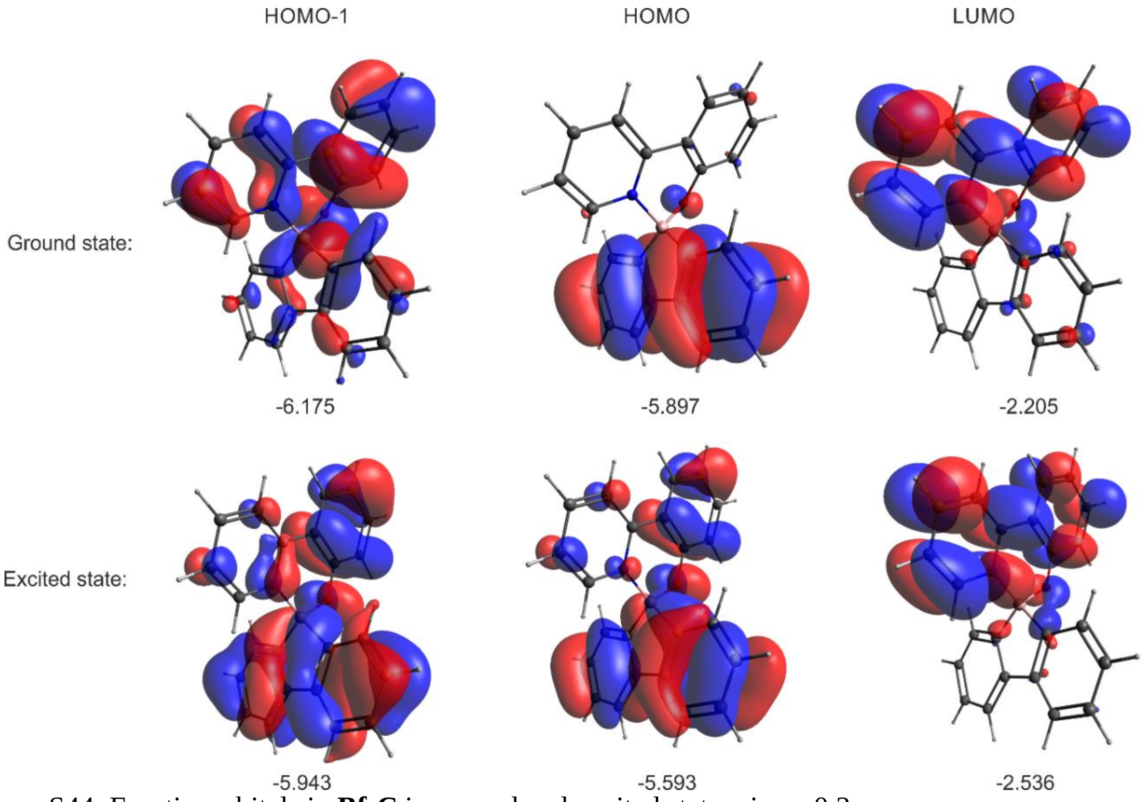

Figure S44. Frontier orbitals in **Bf-C** in ground and excited states; *iso* = 0.2.

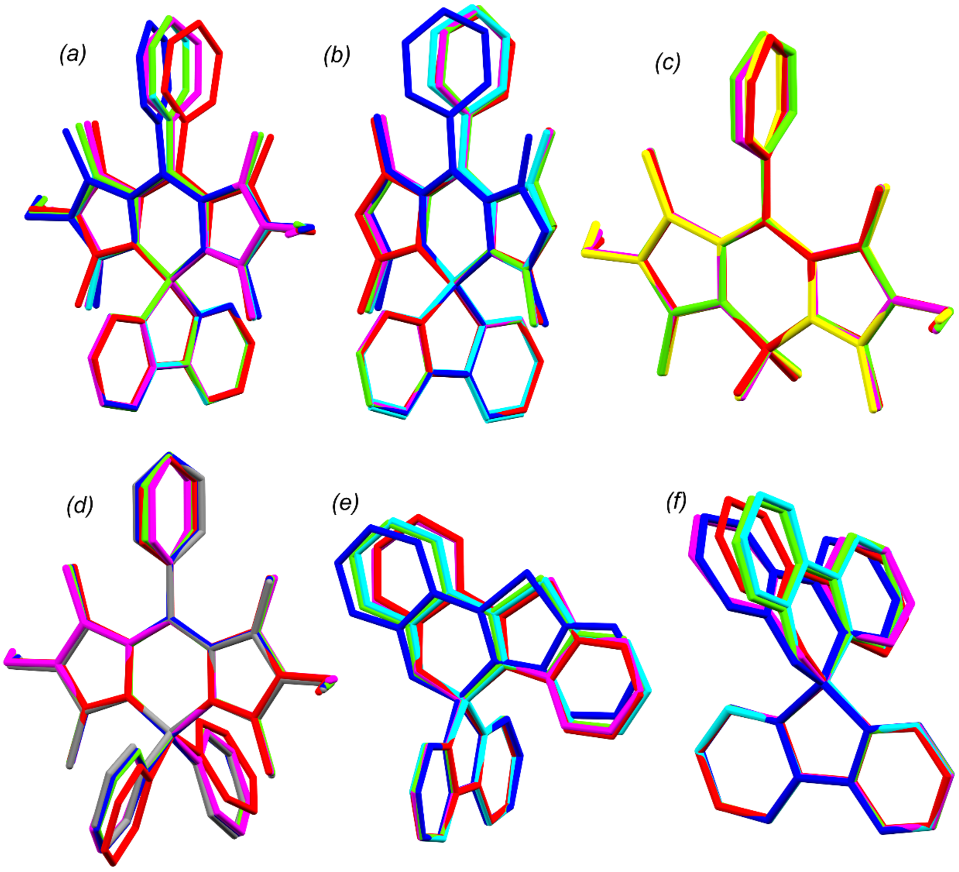

Figure S45. Overlay of structures taken from crystal structure (red), after optimisations *in vacuo*: in ground state (magenta), in [1]CT (cyan), [1]LE (blue), singlet excited (yellow – only **BF$_2$-A1**), triplet excited state (green), HLCT (gray) for *(a)* **Bf-A1**, (b) **Bf-B3** *(c)* **BF$_2$-A1** and *(d)* **BPh$_2$-A1** *(e)* **Bf-B1** and *(f)* **Bf-C**.

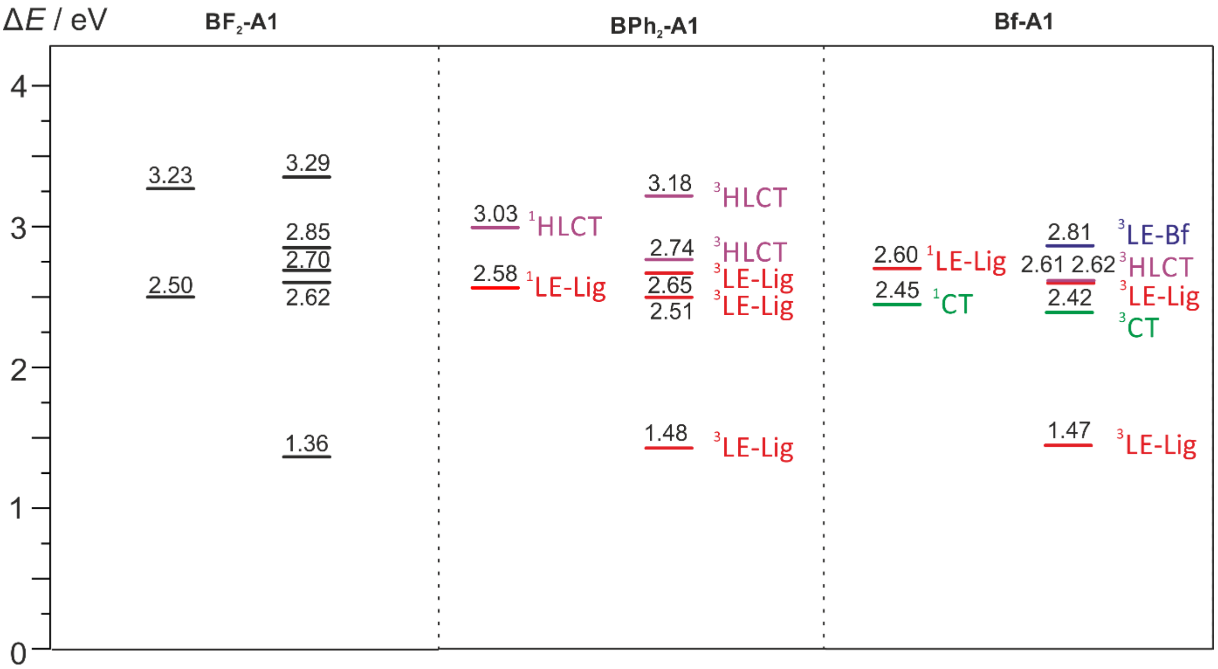

Figure S46. Diagrams illustrating relative energies of two most stable singlet and five triplet excited states in **BF₂-A1**, **BPh₂-A1** and **Bf-A1**.

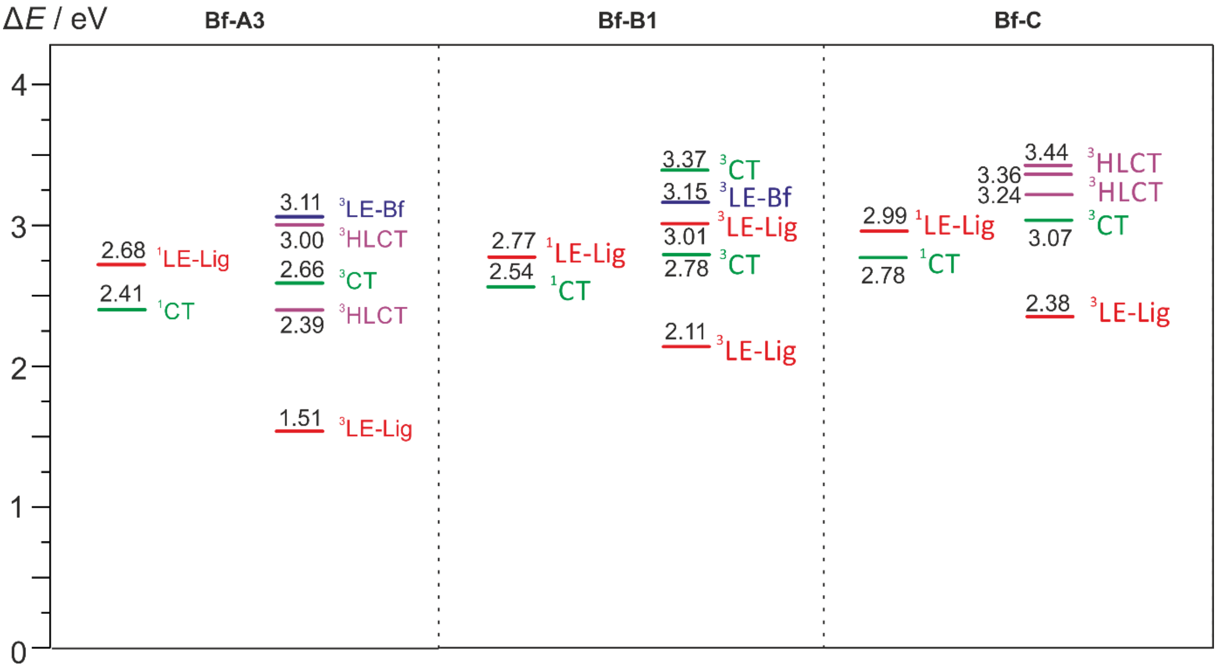

Figure S47. Diagrams illustrating relative energies of two most stable singlet and five triplet excited states in **Bf-A3**, **Bf-B1** and **Bf-C.**

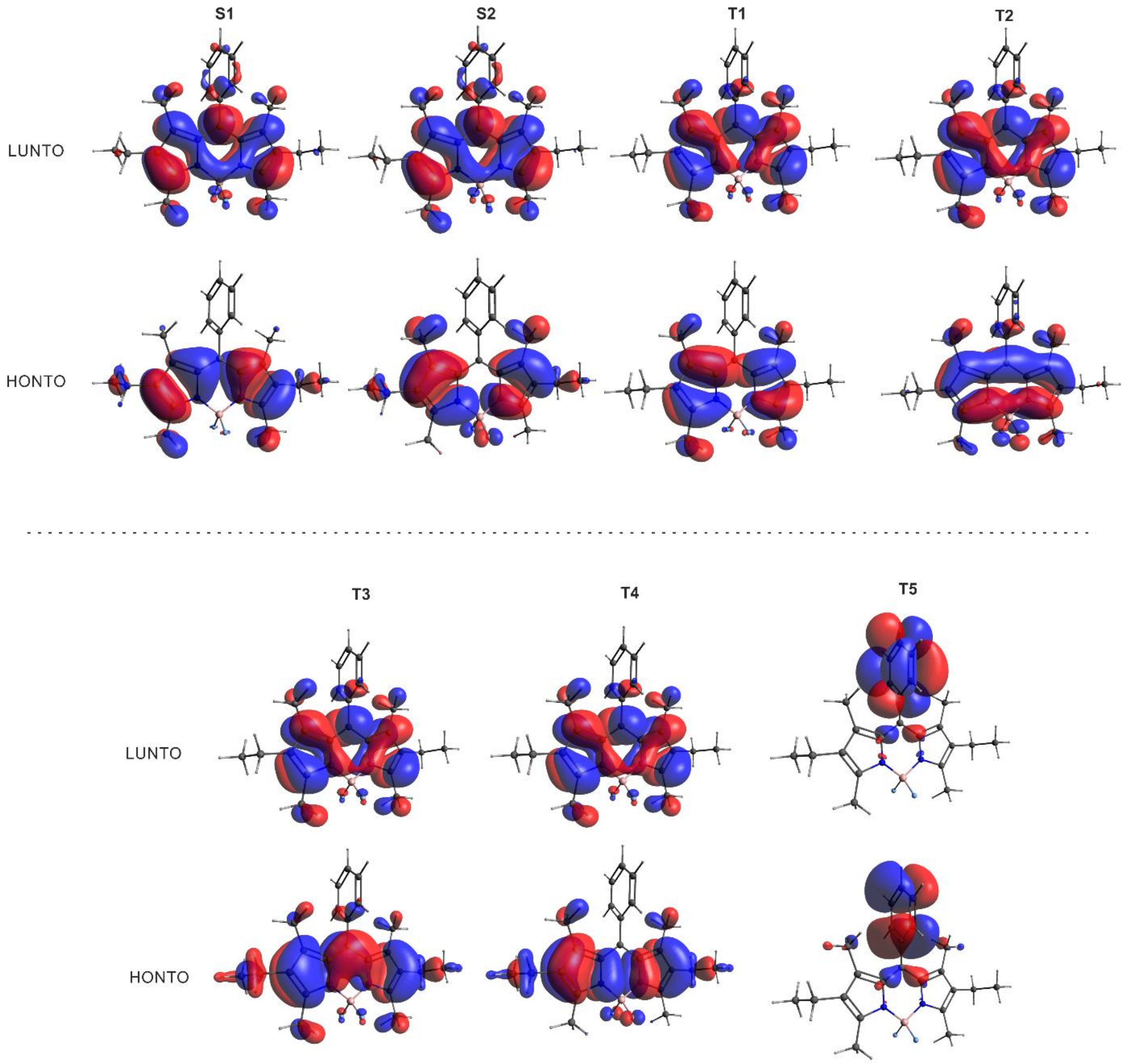

Figure S48. NTO orbitals for singlet and triplet excited states of **BF$_2$-A1**; *iso* = 0.2.

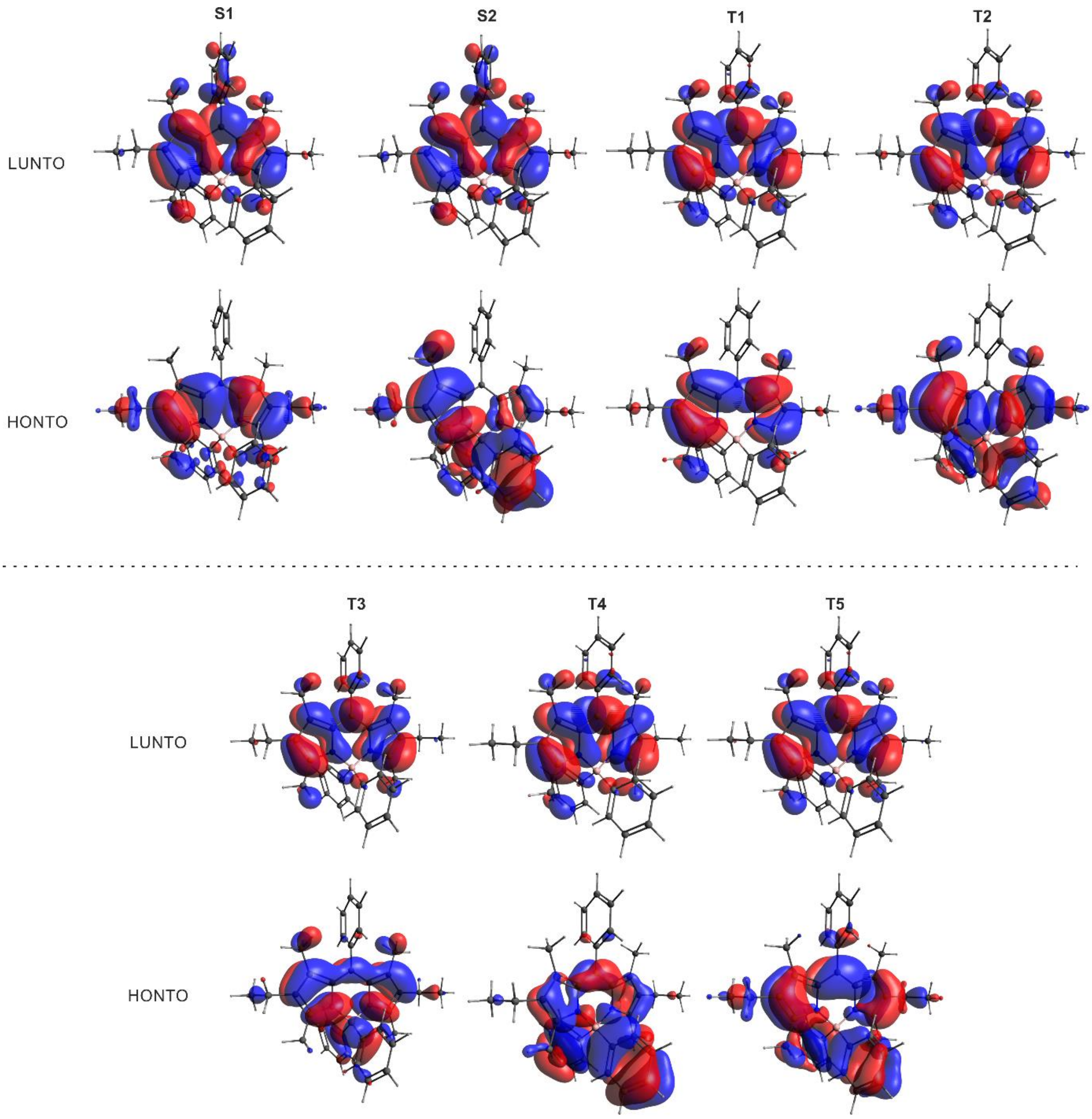

Figure S49. NTO orbitals for singlet and triplet excited states of **BPh$_2$-A1**; *iso* = 0.2.

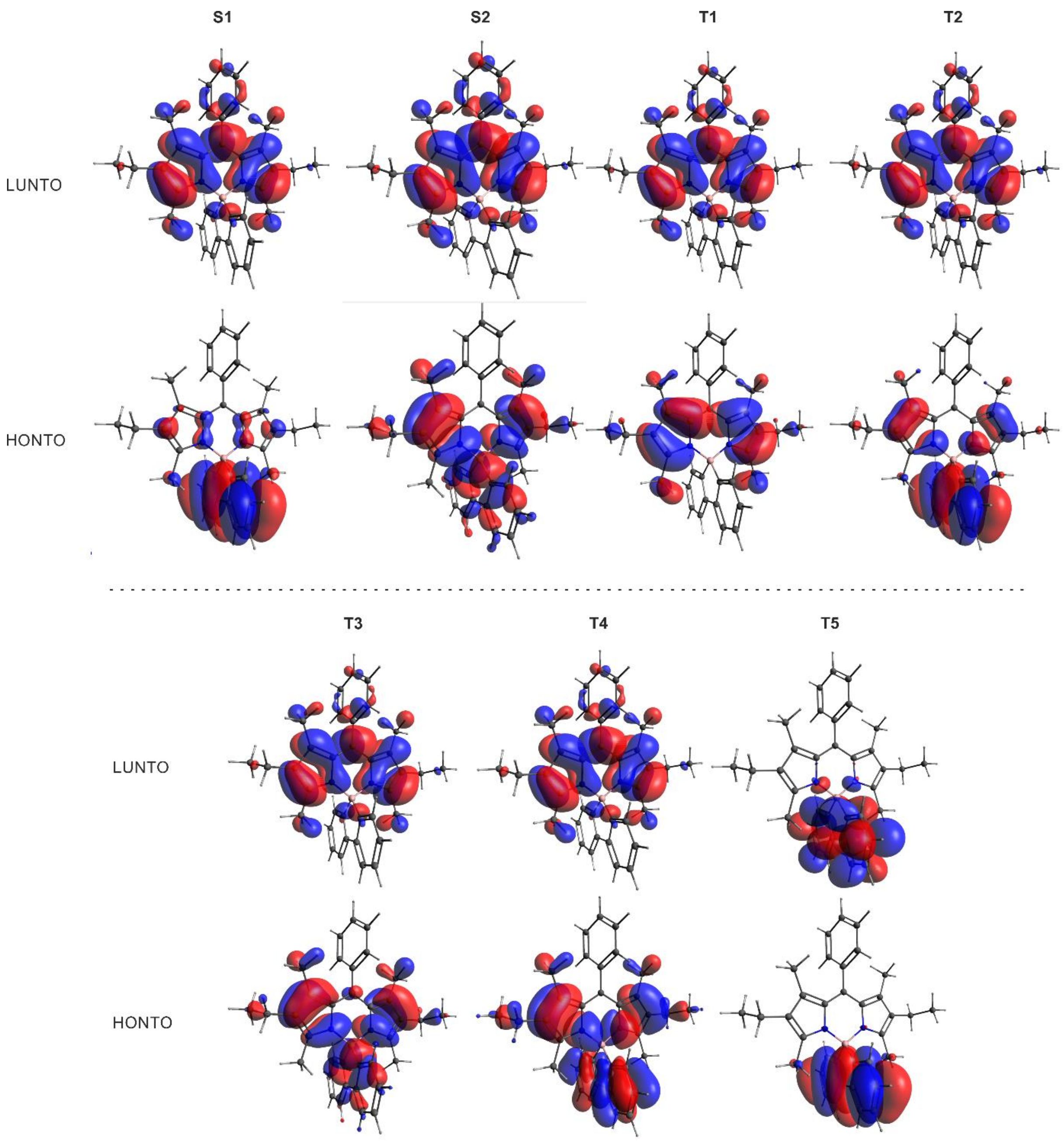

Figure S50. NTO orbitals for singlet and triplet excited states of **Bf-A1**; *iso* = 0.2.

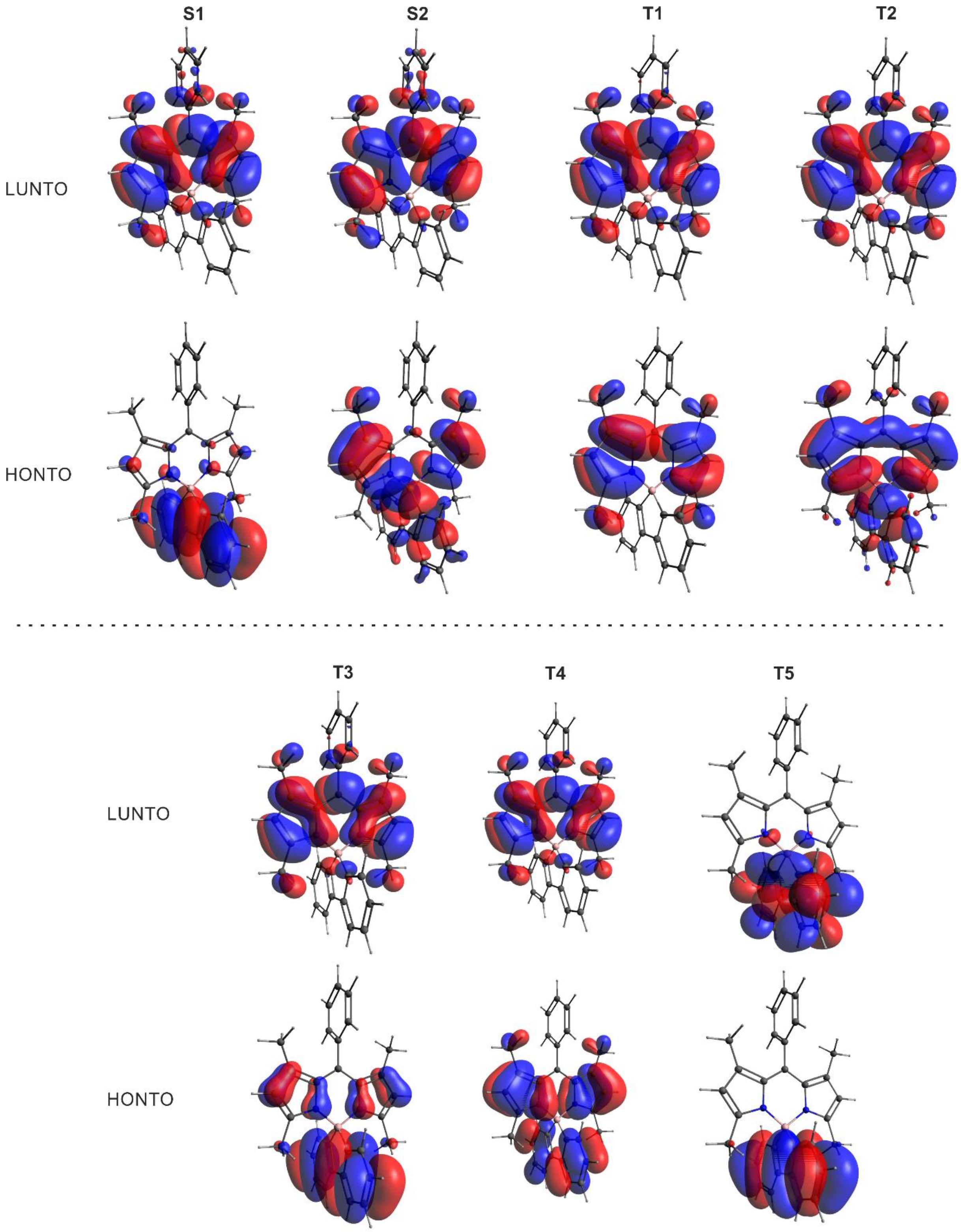

Figure S51. NTO orbitals for singlet and triplet excited states of **Bf-A3**; *iso* = 0.2.

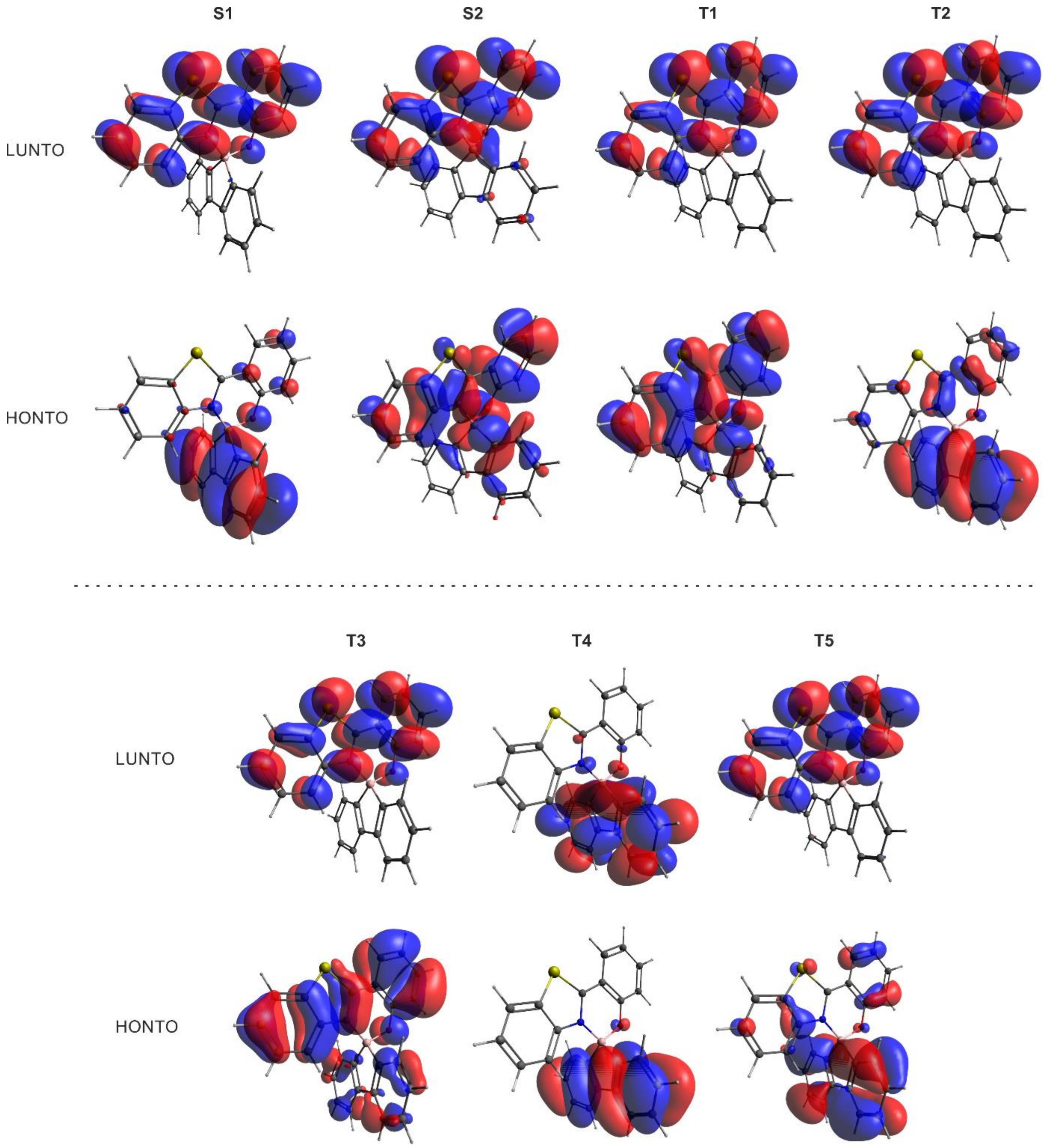

Figure S52. NTO orbitals for singlet and triplet excited states of **Bf-B1**; *iso* = 0.2.

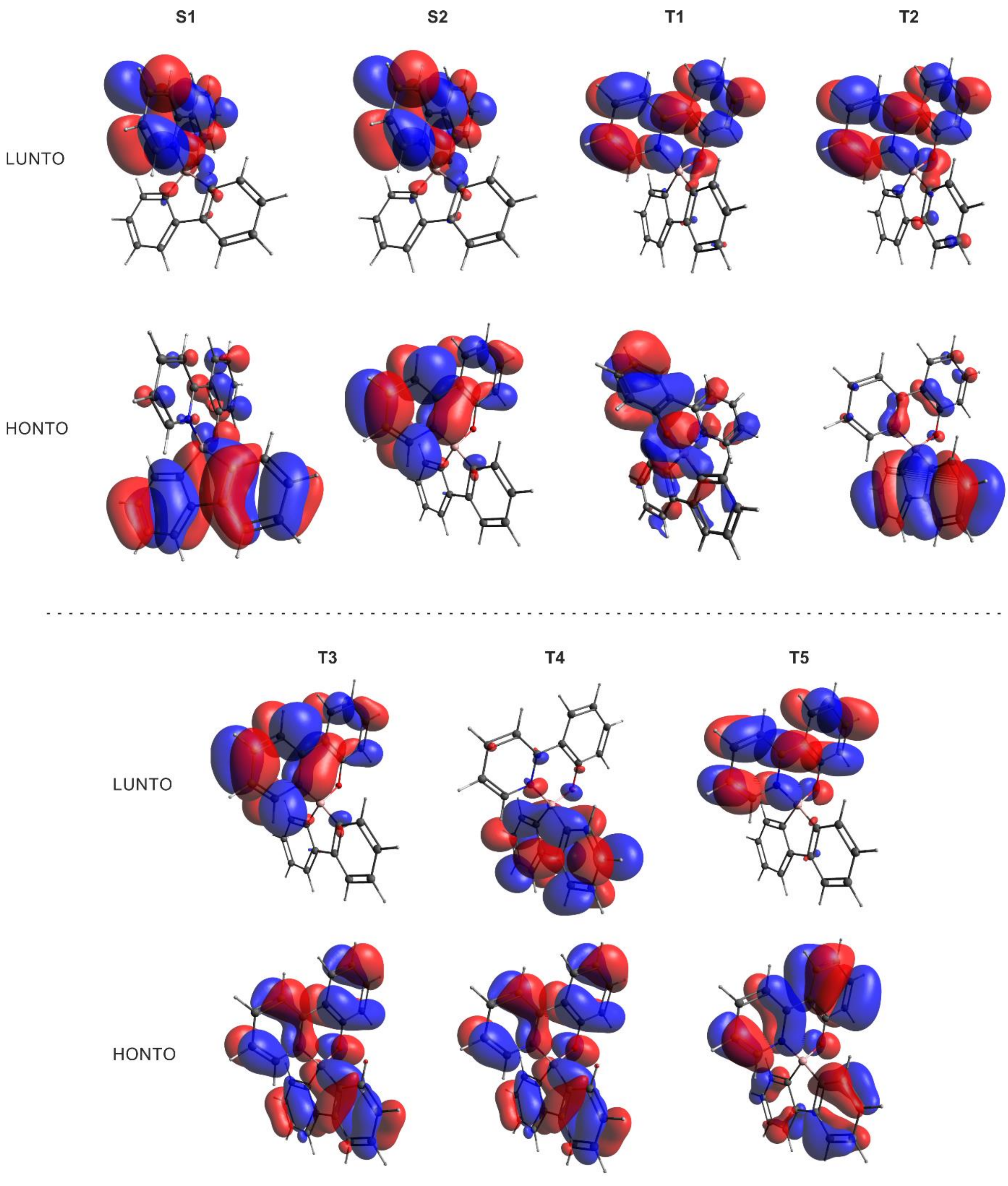

Figure S53. NTO orbitals for singlet and triplet excited states of **Bf-C**; *iso* = 0.2.

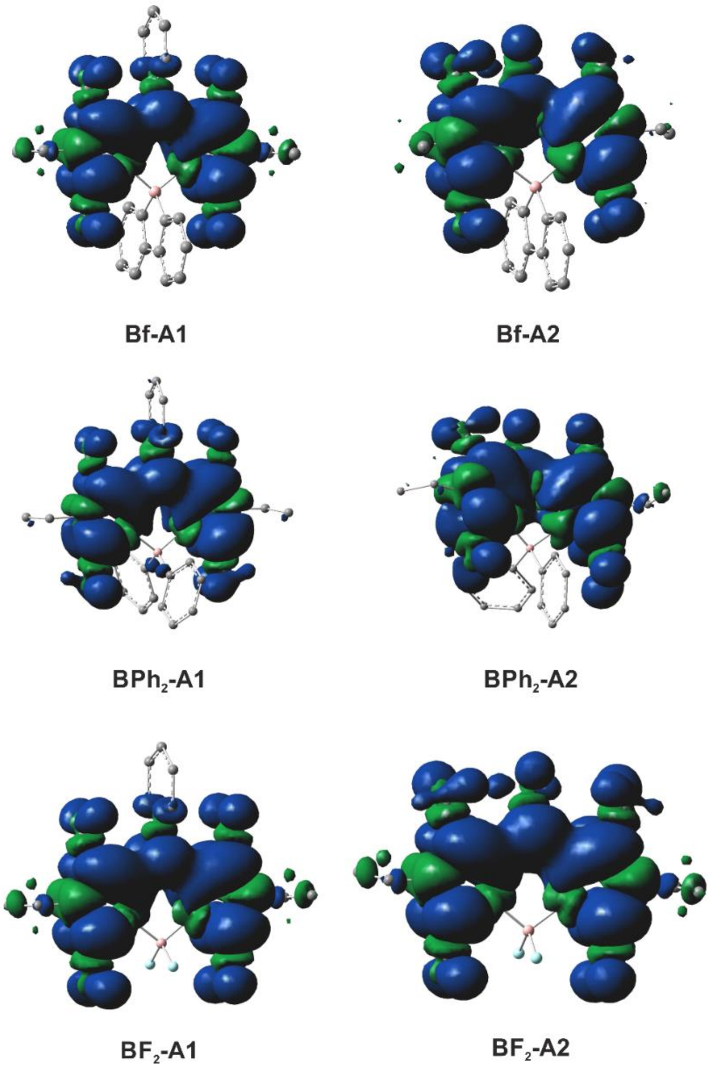

Figure S54. Spin density (*iso* = 0.0004) for all studied BODIPYs in theirs optimised triplet states. Hydrogen atoms were omitted for clarity.

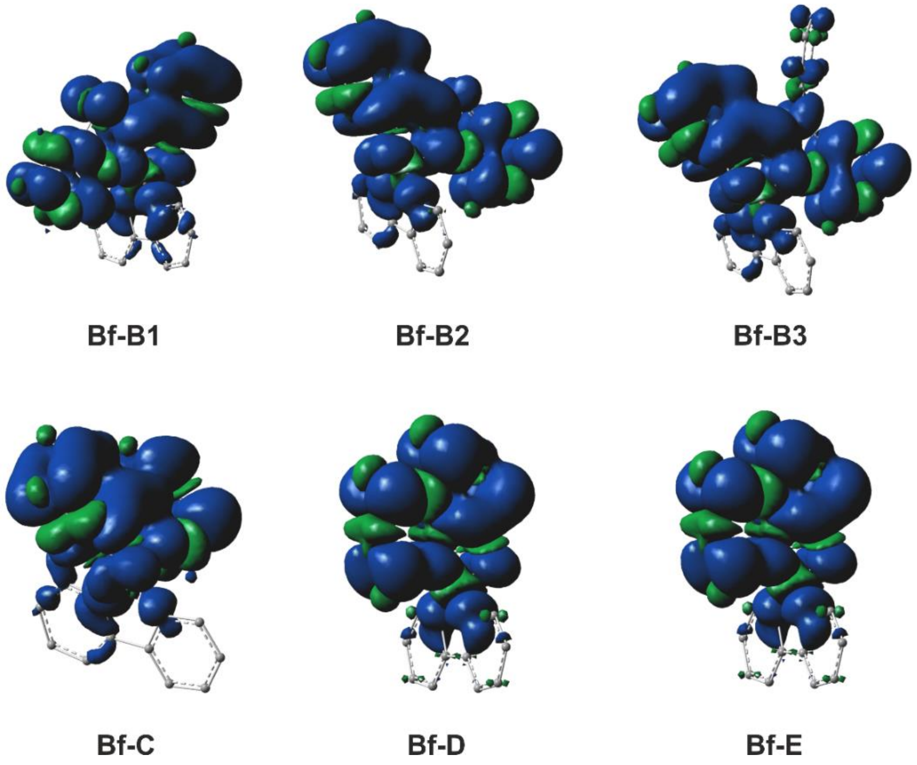

Figure S55. Spin density (*iso* = 0.0004) for borafluorene complexes (except BODIPYs) in theirs optimised triplet states. Hydrogen atoms were omitted for clarity.

Table S6. Computed total energy values for the optimised ground states structures of studied boron complexes together with number of imaginary frequencies.

**Complex**	*E* / a.u	Number of imaginary frequencies
**BF_2-A1**	-33389.07569	0
**BF_2-A2**	-28171.45958	0
**BPh_2-A1**	-40558.92732	0
**BPh_2-A2**	-35341.29469	0
**Bf-A1**	-1489.32333	0
**Bf-A2**	-35308.94270	0
**Bf-A3**	-36247.29698	0
**Bf-B1**	-41238.04654	0
**Bf-B2**	-32449.57805	0
**Bf-B3**	-38196.72628	0
**Bf-C**	-28328.59366	0
**Bf-D**	-30434.94584	0
**Bf-E**	-26221.83405	0

Table S7. Computed total energy values for the optimised singlet and triplet excited states structures.

Complex	State	*E* / a.u	Complex	State	*E* / a.u
**BF_2-A1**	S_1	-33386.57101	**BF_2-A2**	S_1	-28168.92294
	S_2	-33388.95711		S_2	-28171.33762
	T_1	-33388.94965		T_1	-28171.32986
	T_2	-33389.02582		T_2	-28171.40659
	T_3	-33388.97944		T_3	-28171.35793
	T_4	-33388.97630		T_4	-28171.35324
	T_5	-33388.97083		T_5	-28171.33905
**BPh_2-A1**	S_1	-40556.35056	**BPh_2-A2**	S_1	-35338.75592
	S_2	-40558.81603		S_2	-35341.17625
	T_1	-40558.81445		T_1	-35341.17215
	T_2	-40558.87305		T_2	-35341.23969
	T_3	-40558.83491		T_3	-35341.19673
	T_4	-40558.82977		T_4	-35341.19058
	T_5	-40558.82668		T_5	-35341.17622
**Bf-A1**	S_1	-1489.23336	**Bf-A2**	S_1	-35306.41189
	S_2	-1489.22783		S_2	-35308.84680
	T_1	-1489.26946		T_1	-35308.84000
	T_2	-1489.23446		T_2	-35308.88654
	T_3	-1489.22733		T_3	-35308.85108
	T_4	-1489.22696		T_4	-35308.84625
	T_5	-1489.21990		T_5	-35308.84334
**Bf-A3**	S_1	-36244.88259	**Bf-B1**	S_1	-41235.50494
	S_2	-36247.19864		S_2	-41237.94490
	T_1	-36247.24154		T_1	-41237.96908
	T_2	-36247.20720		T_2	-41237.94455
	T_3	-36247.19915		T_3	-41237.93581
	T_4	-36247.18325		T_4	-41237.93092
	T_5	-36247.18287		T_5	-41237.92257
**Bf-B2**	S_1	-32446.87026	**Bf-B3**	S_1	-38193.81276
	S_2	-32449.46488		S_2	-38196.60650
	T_1	-32449.43437		T_1	-38196.57611
	T_2	-32449.49440		T_2	-38196.63749
	T_3	-32449.47136		T_3	-38196.60955
	T_4	-32449.46194		T_4	-38196.60712
	T_5	-32449.46109		T_5	-38196.60331
**Bf-C**	S_1	-28325.80946	**Bf-D**	S_1	-30432.57058
	S_2	-28328.48361		S_2	-30434.84739
	T_1	-28328.50629		T_1	-30434.87413
	T_2	-28328.48083		T_2	-30434.85042
	T_3	-28328.47448		T_3	-30434.84136
	T_4	-28328.47011		T_4	-30434.82474
	T_5	-28328.46718		T_5	-30434.81842
**Bf-E**	S_1	-26219.19893			
	S_2	-26221.72730			
	T_1	-26221.76731			
	T_2	-26221.72628			
	T_3	-26221.71363			
	T_4	-26221.69920			
	T_5	-26221.69559			

Table S8. Atomic coordinates for optimised structure of **Bf-A1** in its ground state.

	x	*y*	*z*		*x*	*y*	*z*		*x*	*y*	*z*
C	0.1146	9.1511	12.0280	C	-1.4383	12.4569	15.2045	H	1.2602	13.0468	20.7066
C	-0.4583	8.0187	12.6092	C	-2.2603	12.4717	14.0756	C	1.6203	13.5589	18.6393
H	-0.5728	7.9554	13.6913	C	-1.5724	11.7807	13.0418	H	2.0298	14.5398	18.8669
C	-0.8938	6.9472	11.8112	C	0.9032	11.4278	15.5737	C	1.5266	13.1395	17.3094
H	-1.3390	6.0684	12.2728	C	5.2380	8.9186	14.6476	H	1.8623	13.7938	16.5087
C	-0.7556	7.0082	10.4192	H	5.8703	9.4618	15.3598	B	0.7073	10.5274	12.6836
H	-1.0932	6.1778	9.8033	H	5.7543	8.9921	13.6826	N	1.9054	10.2630	13.6749
C	-0.1833	8.1361	9.8148	C	5.1708	7.4374	15.0686	N	-0.3745	11.3408	13.4936
H	-0.0802	8.1740	8.7320	H	4.7042	7.3312	16.0552	C	3.7485	10.4907	16.9580
C	0.2474	9.1978	10.6181	H	6.1763	7.0016	15.1185	H	3.7782	11.5499	17.2322
C	0.8734	10.4718	10.1905	H	4.5813	6.8496	14.3549	H	4.7651	10.0964	17.0393
C	1.1679	10.8943	8.8892	C	-3.6158	13.1172	13.9402	H	3.1384	9.9850	17.7144
H	0.9395	10.2663	8.0302	H	-4.1322	13.0954	14.9071	C	-1.7848	13.0892	16.5237
C	1.7662	12.1470	8.6948	H	-4.2426	12.5271	13.2604	H	-1.7416	12.3741	17.3512
H	1.9988	12.4843	7.6873	C	-3.5618	14.5727	13.4358	H	-2.7969	13.5017	16.4917
C	2.0650	12.9643	9.7917	H	-2.9838	15.2040	14.1211	H	-1.1037	13.9087	16.7779
H	2.5293	13.9353	9.6325	H	-4.5716	14.9936	13.3546	C	-2.0742	11.5633	11.6502
C	1.7654	12.5310	11.0945	H	-3.0891	14.6318	12.4481	H	-2.1093	10.4995	11.3951
H	2.0039	13.1795	11.9375	C	1.0022	11.8769	16.9987	H	-1.4272	12.0487	10.9115
C	1.1696	11.2875	11.3107	C	0.5718	11.0404	18.0381	H	-3.0798	11.9748	11.5412
C	1.9650	10.7023	15.0092	H	0.1641	10.0599	17.8049	C	3.3663	8.9595	12.0927
C	3.2163	10.2753	15.5685	C	0.6633	11.4601	19.3681	H	3.3107	9.6821	11.2726
C	3.8890	9.5853	14.5579	H	0.3252	10.8024	20.1647	H	2.6616	8.1540	11.8586
C	3.0518	9.5939	13.4095	C	1.1883	12.7205	19.6724	H	4.3722	8.5348	12.1072
C	-0.2492	11.7426	14.8352								

Table S9. Atomic coordinates for optimised structure of **Bf-A1** in its S_1 singlet excited state.

	x	*y*	*z*		*x*	*y*	*z*		*x*	*y*	*z*
C	0.0693	9.1820	12.0315	C	-0.2551	11.7539	14.8140	C	1.1632	12.6869	19.7063
C	-0.5622	8.0789	12.6066	C	-1.4566	12.4229	15.1661	H	1.2294	13.0031	20.7444
H	-0.6935	8.0230	13.6851	C	-2.2553	12.4824	13.9931	C	1.3545	13.6114	18.6732
C	-1.0317	7.0366	11.8020	C	-1.5498	11.8449	12.9651	H	1.5731	14.6511	18.9061
H	-1.5235	6.1797	12.2550	C	0.9008	11.4299	15.5819	C	1.2666	13.2021	17.3396
C	-0.8784	7.0743	10.3932	C	5.2428	8.9119	14.6017	H	1.4170	13.9258	16.5418
H	-1.2529	6.2500	9.7935	H	5.9308	9.5730	15.1461	B	0.7130	10.5399	12.6965
C	-0.2568	8.1515	9.7857	H	5.6819	8.7879	13.6046	N	1.8854	10.2328	13.6696
H	-0.1393	8.1801	8.7063	C	5.2194	7.5405	15.3069	N	-0.3404	11.3934	13.4565
C	0.2182	9.2074	10.6001	H	4.8234	7.6274	16.3257	C	3.8255	10.5702	16.8733
C	0.8885	10.4126	10.1730	H	6.2294	7.1154	15.3705	H	3.6484	11.5964	17.2082
C	1.2151	10.7929	8.8494	H	4.5853	6.8311	14.7613	H	4.9098	10.4151	16.8481
H	0.9650	10.1565	8.0057	C	-3.6075	13.1364	13.8746	H	3.4262	9.9113	17.6566
C	1.8639	11.9989	8.6475	H	-4.2064	12.9140	14.7683	C	-1.8806	12.9718	16.5024

H	2.1271	12.3164	7.6429	H	-4.1630	12.7042	13.0337	H	-1.6321	12.3000	17.3293
C	2.1900	12.8303	9.7485	C	-3.5465	14.6667	13.6935	H	-2.9650	13.1279	16.5201
H	2.6993	13.7725	9.5637	H	-3.0326	15.1436	14.5366	H	-1.4143	13.9395	16.7335
C	1.8671	12.4551	11.0559	H	-4.5553	15.0938	13.6256	C	-1.9947	11.6548	11.5503
H	2.1294	13.1130	11.8816	H	-3.0028	14.9306	12.7782	H	-2.0991	10.5959	11.2772
C	1.2140	11.2466	11.3001	C	0.9912	11.8643	17.0062	H	-1.2970	12.0979	10.8273
C	1.9710	10.6932	14.9965	C	0.8038	10.9479	18.0558	H	-2.9674	12.1291	11.3966
C	3.2297	10.2947	15.5183	H	0.5873	9.9089	17.8181	C	3.3146	8.8926	12.0572
C	3.8892	9.5648	14.4938	C	0.8859	11.3517	19.3915	H	3.3439	9.6118	11.2274
C	3.0457	9.5435	13.3762	H	0.7326	10.6251	20.1861	H	2.5554	8.1433	11.7963
H	4.2808	8.3822	12.0813								

Table S10. Atomic coordinates for optimised structure of **Bf-A1** in its S_2 singlet excited state.

	x	*y*	*z*		*x*	*y*	*z*		*x*	*y*	*z*
C	0.0309	9.2013	12.1194	C	-1.4950	12.4019	15.1442	H	1.0641	12.7033	20.8148
C	-0.6098	8.1578	12.7896	C	-2.2219	12.5631	13.9569	C	1.1433	13.4371	18.7826
H	-0.7112	8.1808	13.8737	C	-1.4538	12.0150	12.9050	H	1.2760	14.4759	19.0753
C	-1.1282	7.0681	12.0749	C	0.8812	11.4044	15.5760	C	1.0922	13.1002	17.4277
H	-1.6264	6.2589	12.6035	C	5.3229	9.0538	14.5804	H	1.1864	13.8778	16.6735
C	-1.0082	7.0124	10.6742	H	5.9684	9.6948	15.1944	B	0.7353	10.5720	12.6667
H	-1.4123	6.1626	10.1301	H	5.7930	9.0299	13.5903	N	1.9164	10.2843	13.6566
C	-0.3728	8.0424	9.9779	C	5.3322	7.6311	15.1743	N	-0.2743	11.5060	13.4192
H	-0.2850	7.9903	8.8953	H	4.9089	7.6253	16.1858	C	3.8380	10.6812	16.8553
C	0.1423	9.1291	10.6996	H	6.3554	7.2390	15.2312	H	3.6296	11.7132	17.1570
C	0.8423	10.3095	10.1749	H	4.7386	6.9434	14.5598	H	4.9239	10.5423	16.8585
C	1.1329	10.6113	8.8362	C	-3.5652	13.2326	13.8132	H	3.4275	10.0405	17.6487
H	0.8375	9.9443	8.0299	H	-4.1839	13.0011	14.6897	C	-1.9655	12.8034	16.5104
C	1.8172	11.7928	8.5454	H	-4.1056	12.8145	12.9559	H	-1.7552	12.0358	17.2627
H	2.0502	12.0416	7.5133	C	-3.4853	14.7642	13.6560	H	-3.0462	12.9792	16.5026
C	2.2064	12.6629	9.5805	H	-2.9908	15.2224	14.5208	H	-1.4905	13.7266	16.8714
H	2.7384	13.5799	9.3389	H	-4.4885	15.1999	13.5672	C	-1.8144	12.0365	11.4513
C	1.9111	12.3552	10.9171	H	-2.9151	15.0390	12.7605	H	-1.8348	11.0389	11.0012
H	2.2189	13.0433	11.7030	C	0.9297	11.7636	17.0195	H	-1.1153	12.6448	10.8654
C	1.2272	11.1825	11.2357	C	0.8181	10.7741	18.0133	H	-2.8078	12.4740	11.3295
C	1.9777	10.7224	14.9681	H	0.6888	9.7361	17.7163	C	3.4000	8.9396	12.0629
C	3.2760	10.3618	15.5025	C	0.8628	11.1083	19.3692	H	3.4607	9.6351	11.2197
C	3.9485	9.6673	14.4879	H	0.7677	10.3279	20.1204	H	2.6488	8.1829	11.8108
C	3.0981	9.6240	13.3615	C	1.0270	12.4422	19.7602	H	4.3633	8.4301	12.1373
C	-0.2484	11.7586	14.7816								

Table S11. Atomic coordinates for optimised structure of **Bf-A1** in its T_1 triplet excited state.

	x	*y*	*z*		*x*	*y*	*z*		*x*	*y*	*z*
C	-2.2132	-0.1018	1.2363	C	1.6356	2.5548	0.3499	H	7.2925	-0.0056	0.0145
C	-2.0283	-0.2143	2.6145	C	0.4851	3.3862	0.4011	C	5.5039	0.4344	-1.1181
H	-1.0243	-0.2500	3.0318	C	-0.6393	2.5666	0.2430	H	6.0453	0.7737	-1.9982

C	-3.1313	-0.2818	3.4707	C	1.8857	-0.0003	-0.0041	C	4.1060	0.4367	-1.1189
H	-2.9818	-0.3689	4.5436	C	0.4781	-4.8809	-0.5968	H	3.5673	0.7784	-1.9998
C	-4.4545	-0.2386	2.9634	H	1.2344	-5.1706	-1.3391	B	-1.1397	0.0006	-0.0037
H	-5.2921	-0.2932	3.6524	H	-0.4822	-5.2084	-1.0127	N	-0.2296	-1.2544	-0.1136
C	-4.6785	-0.1279	1.6020	C	0.7458	-5.6615	0.7062	N	-0.2289	1.2562	0.0933
H	-5.6923	-0.0949	1.2137	H	1.7186	-5.3923	1.1346	C	3.0450	-3.0577	-0.5224
C	-3.5618	-0.0588	0.7349	H	0.7438	-6.7435	0.5222	H	3.6555	-2.4030	-1.1513
C	-3.5731	0.0588	-0.7039	H	-0.0225	-5.4426	1.4578	H	3.0423	-4.0503	-0.9862
C	-4.7034	0.1277	-1.5531	C	0.4809	4.8833	0.5705	H	3.5736	-3.1539	0.4360
H	-5.7108	0.0938	-1.1487	H	1.2309	5.1736	1.3189	C	3.0454	3.0571	0.5144
C	-4.5010	0.2396	-2.9178	H	-0.4825	5.2142	0.9764	H	3.6518	2.4036	1.1485
H	-5.3495	0.2942	-3.5934	C	0.7623	5.6592	-0.7323	H	3.0412	4.0510	0.9753
C	-3.1862	0.2845	-3.4460	H	1.7384	5.3868	-1.1508	H	3.5793	3.1500	-0.4414
H	-3.0538	0.3727	-4.5211	H	0.7608	6.7419	-0.5516	C	-2.0721	2.9943	0.2358
C	-2.0697	0.2169	-2.6075	H	0.0005	5.4395	-1.4903	H	-2.6506	2.5529	1.0588
H	-1.0724	0.2538	-3.0406	C	3.3775	-0.0013	0.0009	H	-2.5882	2.7186	-0.6934
C	-2.2327	0.1028	-1.2266	C	4.0973	-0.4407	1.1257	H	-2.1392	4.0807	0.3354
C	1.1767	-1.2273	-0.1542	H	3.5515	-0.7810	2.0028	C	-2.0734	-2.9912	-0.2630
C	1.6348	-2.5544	-0.3639	C	5.4951	-0.4414	1.1347	H	-2.6514	-2.5465	-1.0844
C	0.4838	-3.3843	-0.4228	H	6.0296	-0.7818	2.0186	H	-2.5897	-2.7190	0.6672
C	-0.6405	-2.5640	-0.2677	C	6.2053	-0.0044	0.0107	H	-2.1410	-4.0772	-0.3668
C	1.1772	1.2277	0.1411								

Table S12. Atomic coordinates for optimised structure of **Bf-A2** in its ground state.

	x	*y*	*z*		*x*	*y*	*z*		*x*	*y*	*z*
N	3.3803	6.5400	3.2103	H	3.2613	8.9611	0.8311	H	6.2738	1.6201	0.3600
N	5.6617	7.5077	2.7426	C	4.3369	5.9026	-0.2429	C	6.1185	2.8439	2.1328
C	2.2729	5.7603	3.1943	C	0.1168	5.4269	4.6108	H	6.5683	2.0947	2.7808
C	5.4776	8.3730	3.8343	H	-0.4155	5.1673	3.6870	C	9.0503	10.4512	1.8867
C	3.2621	7.4350	4.2872	H	-0.5391	6.1293	5.1380	H	8.6223	10.2521	0.8969
C	1.4200	6.1064	4.2745	C	8.9765	9.2007	2.7850	H	10.0909	10.7702	1.7491
C	2.0019	4.7039	2.1703	H	9.5822	8.4032	2.3384	H	8.4941	11.2861	2.3293
H	2.1230	5.0868	1.1526	H	9.4538	9.4274	3.7463	C	3.4200	7.1832	-2.0762
H	0.9799	4.3321	2.2752	C	5.7043	4.0778	2.6624	H	3.1664	7.2735	-3.1300
H	2.6833	3.8525	2.2782	H	5.8399	4.2711	3.7265	C	4.0486	9.4195	5.6286
C	6.9033	7.7239	2.2456	C	1.4804	7.7760	6.2232	H	4.3999	9.0732	6.6097
C	4.0710	6.9648	0.6575	H	2.2325	7.8532	7.0148	H	2.9931	9.6669	5.7241
C	4.2711	8.3774	4.5590	H	0.6581	7.1716	6.6155	H	4.5773	10.3420	5.3955
C	2.0319	7.1537	4.9686	H	1.0826	8.7853	6.0523	C	3.1510	8.2415	-1.1995
C	6.6853	9.1237	4.0180	C	4.0151	6.0065	-1.6014	H	2.6887	9.1516	-1.5758
C	7.4604	7.0276	1.0441	H	4.2204	5.1894	-2.2904	C	5.3740	3.5346	-0.0747
H	7.6258	5.9612	1.2338	C	7.0224	10.1287	5.0856	H	5.2494	3.3167	-1.1338
H	8.4157	7.4738	0.7587	H	6.5275	11.0953	4.9222	B	4.5583	6.5431	2.1635
H	6.7830	7.1007	0.1879	H	8.0987	10.3230	5.0946	C	0.2816	4.1549	5.4657

C	7.5722	8.7057	3.0213	H	6.7435	9.7857	6.0870	H	0.8958	3.4079	4.9489
C	5.1261	5.0460	1.8404	C	4.9653	4.7599	0.4627	H	-0.6940	3.7018	5.6809
C	3.4774	8.1278	0.1625	C	5.9527	2.5757	0.7685	H	0.7686	4.3841	6.4211

Table S13. Atomic coordinates for optimised structure of **Bf-A2** in its S_1 singlet excited state.

	x	*y*	*z*		*x*	*y*	*z*		*x*	*y*	*z*
N	3.3639	6.5880	3.1708	H	3.2544	8.9394	0.7094	H	6.2786	1.5966	0.4728
N	5.6370	7.5400	2.7001	C	4.3460	5.8464	-0.2578	C	6.1060	2.8719	2.2106
C	2.2366	5.8187	3.1338	C	0.1090	5.4606	4.5890	H	6.5523	2.1394	2.8794
C	5.4984	8.3598	3.8437	H	-0.4321	5.2098	3.6680	C	9.0224	10.5754	2.0474
C	3.2756	7.4329	4.3016	H	-0.5314	6.1687	5.1292	H	8.5471	10.5338	1.0603
C	1.4169	6.1229	4.2534	C	8.9734	9.1983	2.7443	H	10.0612	10.9016	1.9125
C	1.9410	4.8406	2.0526	H	9.5376	8.4767	2.1424	H	8.5009	11.3357	2.6403
H	1.9416	5.3167	1.0615	H	9.5075	9.2736	3.7011	C	3.4456	7.0616	-2.1392
H	0.9631	4.3791	2.2094	C	5.6883	4.1151	2.7066	H	3.1998	7.1222	-3.1965
H	2.6929	4.0403	2.0034	H	5.8158	4.3363	3.7658	C	4.0376	9.4866	5.5977
C	6.8781	7.7598	2.1745	C	1.5733	7.6776	6.3079	H	4.3572	9.2178	6.6157
C	4.0697	6.9408	0.6116	H	2.3826	7.8189	7.0313	H	2.9761	9.7384	5.6510
C	4.2807	8.4007	4.5796	H	0.8514	6.9913	6.7625	H	4.5700	10.4006	5.3251
C	2.0695	7.1217	4.9995	H	1.0646	8.6462	6.1988	C	3.1666	8.1502	-1.2941
C	6.7251	9.0620	4.0428	C	4.0359	5.9053	-1.6270	H	2.7065	9.0452	-1.7067
C	7.3655	7.1164	0.9249	H	4.2489	5.0670	-2.2864	C	5.3808	3.4918	-0.0248
H	7.3900	6.0206	1.0074	C	7.1086	9.9738	5.1770	H	5.2644	3.2458	-1.0778
H	8.3718	7.4635	0.6796	H	6.7566	11.0061	5.0380	B	4.5410	6.5734	2.1396
H	6.7094	7.3398	0.0713	H	8.1989	10.0246	5.2688	C	0.2602	4.1790	5.4375
C	7.5794	8.6914	2.9862	H	6.7186	9.6266	6.1388	H	0.8490	3.4220	4.9066
C	5.1144	5.0622	1.8559	C	4.9649	4.7351	0.4790	H	-0.7236	3.7491	5.6635
C	3.4784	8.0853	0.0713	C	5.9517	2.5632	0.8480	H	0.7657	4.3922	6.3867

Table S14. Atomic coordinates for optimised structure of **Bf-A2** in its T_1 triplet excited state.

	x	*y*	*z*		*x*	*y*	*z*		*x*	*y*	*z*
N	3.3877	6.5385	3.2147	H	3.2527	8.9962	0.8554	H	6.2302	1.6491	0.2515
N	5.6505	7.4972	2.7536	C	4.3079	5.9496	-0.2694	C	6.0909	2.8442	2.0452
C	2.2699	5.7101	3.2270	C	0.1513	5.3635	4.6941	H	6.5416	2.0825	2.6779
C	5.4502	8.3756	3.7964	H	-0.4085	5.0989	3.7880	C	9.1251	10.3838	1.9450
C	3.2544	7.4446	4.2442	H	-0.4934	6.0531	5.2507	H	8.7281	10.1711	0.9453
C	1.4286	6.0672	4.3163	C	9.0125	9.1499	2.8627	H	10.1725	10.6902	1.8367
C	2.0182	4.6366	2.2251	H	9.6229	8.3381	2.4496	H	8.5629	11.2306	2.3557
H	2.1417	5.0031	1.2006	H	9.4569	9.3915	3.8359	C	3.3872	7.2614	-2.0786
H	1.0001	4.2540	2.3324	C	5.6850	4.0710	2.5968	H	3.1283	7.3687	-3.1296
H	2.7110	3.7948	2.3475	H	5.8278	4.2469	3.6632	C	3.9003	9.6342	5.3948
C	6.9427	7.6896	2.2749	C	1.5342	7.7683	6.2448	H	4.1790	9.4731	6.4459
C	4.0525	6.9985	0.6503	H	2.3489	7.9471	6.9542	H	2.8330	9.8663	5.3762

C	4.2096	8.4687	4.4815	H	0.8063	7.1177	6.7384	H	4.4170	10.5361	5.0587
C	2.0276	7.1324	4.9765	H	1.0392	8.7336	6.0727	C	3.1281	8.3076	-1.1845
C	6.6935	9.1111	4.0244	C	3.9794	6.0750	-1.6245	H	2.6682	9.2255	-1.5447
C	7.5117	6.9634	1.1046	H	4.1771	5.2674	-2.3269	C	5.3358	3.5741	-0.1464
H	7.6726	5.9006	1.3234	C	6.9865	10.0882	5.1259	H	5.2039	3.3738	-1.2080
H	8.4716	7.4005	0.8196	H	6.5889	11.0913	4.9205	B	4.5469	6.5498	2.1478
H	6.8435	7.0110	0.2382	H	8.0674	10.1966	5.2594	C	0.3733	4.0903	5.5348
C	7.5966	8.6769	3.0611	H	6.5658	9.7648	6.0834	H	0.9806	3.3569	4.9915
C	5.1059	5.0556	1.7947	C	4.9357	4.7922	0.4139	H	-0.5858	3.6196	5.7825
C	3.4615	8.1715	0.1738	C	5.9157	2.5992	0.6775	H	0.8906	4.3242	6.4727

Table S15. Atomic coordinates for optimised structure of **Bf-A3** in its ground state.

	x	*y*	*z*		*x*	*y*	*z*		*x*	*y*	*z*
N	8.7211	2.5600	1.2561	H	11.4324	1.4851	2.7820	H	3.2584	5.2882	3.0644
N	6.4718	3.5204	0.6273	C	9.5966	5.6846	-2.1801	H	4.7852	5.6678	3.8790
C	9.5421	2.4449	3.4095	H	10.0095	6.6895	-2.1231	C	4.9556	4.4001	2.1279
C	8.5397	3.1056	-2.3360	C	5.3808	3.9329	-0.0588	C	6.7112	-0.6659	-1.5559
C	8.4307	2.8623	2.6018	C	7.1713	-0.4382	-2.8586	H	6.2374	-1.6130	-1.3071
C	6.9444	3.7747	4.3963	H	7.0539	-1.2081	-3.6179	C	6.3175	2.8264	5.2169
C	7.2160	3.4685	2.9564	C	7.7834	0.7787	-3.1895	H	6.0276	1.8641	4.8024
C	9.0259	5.0911	-1.0414	H	8.1374	0.9459	-4.2049	C	6.8615	0.3272	-0.5730
H	9.0037	5.6503	-0.1060	C	9.9452	1.9846	1.2171	H	6.4979	0.1346	0.4363
C	9.7495	2.5321	4.8948	C	7.4679	1.5459	-0.8813	C	6.0647	3.1125	6.5615
H	9.7135	3.5643	5.2588	C	7.3148	5.0132	4.9392	H	5.5780	2.3692	7.1876
H	10.7305	2.1177	5.1490	H	7.8012	5.7532	4.3086	C	10.6384	1.5087	-0.0184
H	8.9913	1.9725	5.4529	C	9.1061	3.6881	-3.4755	H	10.0678	0.7230	-0.5250
C	5.2004	3.8316	-1.5389	H	9.1412	3.1514	-4.4216	H	11.6196	1.1062	0.2486
H	5.9828	4.3736	-2.0805	C	4.4387	4.4749	0.8414	H	10.7807	2.3192	-0.7410
H	4.2307	4.2548	-1.8157	H	3.4743	4.8790	0.5579	C	6.4366	4.3495	7.0984
H	5.2348	2.7915	-1.8802	C	9.6351	4.9834	-3.3914	H	6.2402	4.5718	8.1439
C	8.4931	3.8024	-1.1031	H	10.0773	5.4446	-4.2717	B	7.7770	2.8666	0.0307
C	7.9286	1.7612	-2.2037	C	4.2329	4.8813	3.3539	C	7.0622	5.2990	6.2838
C	6.2496	3.7928	1.9922	H	4.0620	4.0769	4.0771	H	7.3543	6.2626	6.6933
C	10.4666	1.9040	2.5261								

Table S16. Atomic coordinates for optimised structure of **Bf-A3** in its S_1 singlet excited state.

	x	*y*	*z*		*x*	*y*	*z*		*x*	*y*	*z*
N	-0.1423	-1.2590	-0.0194	H	0.5579	-4.4765	0.0260	H	3.0973	4.2058	-0.2349
N	-0.1417	1.2597	0.0186	C	-3.0757	0.0410	-3.4644	H	3.7360	2.6812	-0.8669
C	1.7267	-2.5789	0.0274	H	-2.9369	0.0547	-4.5421	C	1.7279	2.5787	-0.0272
C	-3.4815	0.0066	-0.7186	C	-0.5534	2.5800	0.0119	C	-3.0734	-0.0366	3.4659
C	1.2650	-1.2356	-0.0007	C	-4.3945	-0.0317	2.9463	H	-2.9338	-0.0474	4.5435
C	3.4658	-0.0006	0.0004	H	-5.2364	-0.0384	3.6318	C	4.1901	0.1945	1.1886
C	1.9721	-0.0001	0.0011	C	-4.6074	-0.0179	1.5809	H	3.6482	0.3461	2.1194
C	-1.9635	0.0337	-2.6176	H	-5.6172	-0.0129	1.1817	C	-1.9617	-0.0277	2.6183

H	-0.9643	0.0415	-3.0468	C	-0.5546	-2.5792	-0.0142	H	-0.9622	-0.0312	3.0468
C	3.1297	-3.1237	0.0697	C	-2.1341	-0.0142	1.2350	C	5.5884	0.1934	1.1917
H	3.6811	-2.9547	-0.8643	C	4.1883	-0.1959	-1.1888	H	6.1272	0.3436	2.1244
H	3.0946	-4.2069	0.2372	H	3.6450	-0.3471	-2.1188	C	-1.9777	-3.0438	-0.0317
H	3.7329	-2.6831	0.8714	C	-4.6085	0.0129	-1.5785	H	-2.5478	-2.7123	0.8460
C	-1.9766	3.0448	0.0272	H	-5.6180	0.0040	-1.1787	H	-1.9918	-4.1386	-0.0376
H	-2.5457	2.7118	-0.8505	C	0.5742	3.3922	-0.0148	H	-2.5281	-2.7021	-0.9179
H	-1.9906	4.1396	0.0314	H	0.5599	4.4768	-0.0290	C	6.2928	-0.0013	-0.0016
H	-2.5277	2.7047	0.9134	C	-4.3965	0.0302	-2.9440	H	7.3800	-0.0017	-0.0023
C	-2.1349	0.0161	-1.2343	H	-5.2388	0.0356	-3.6290	B	-1.0499	0.0007	0.0000
C	-3.4810	-0.0092	0.7203	C	3.1312	3.1227	-0.0668	C	5.5866	-0.1955	-1.1938
C	1.2656	1.2357	0.0014	H	3.6804	2.9538	0.8685	H	6.1240	-0.3461	-2.1273
C	0.5727	-3.3919	0.0128								

Table S17. Atomic coordinates for optimised structure of **Bf-A3** in its S_2 singlet excited state.

	x	*y*	*z*		*x*	*y*	*z*		*x*	*y*	*z*
N	-0.1534	-1.2478	-0.2392	H	0.5358	-4.4738	-0.1611	H	3.1397	4.1639	-0.4517
N	-0.1524	1.2652	-0.1348	C	-3.3877	0.1362	-3.3922	H	3.7584	2.5709	-0.9360
C	1.7027	-2.6009	-0.0045	H	-3.3324	0.1817	-4.4768	C	1.7355	2.5921	-0.1499
C	-3.5432	0.0140	-0.6155	C	-0.5445	2.5796	-0.1938	C	-2.7260	-0.1271	3.5557
C	1.2365	-1.2277	-0.0756	C	-4.0760	-0.1272	3.1692	H	-2.4668	-0.1610	4.6109
C	3.4392	-0.0030	0.1184	H	-4.8592	-0.1591	3.9228	C	4.0553	0.3297	1.3382
C	1.9564	-0.0034	-0.0082	C	-4.4230	-0.0833	1.8157	H	3.4375	0.5786	2.1972
C	-2.2075	0.1133	-2.6296	H	-5.4729	-0.0813	1.5269	C	-1.7115	-0.0841	2.5855
H	-1.2424	0.1411	-3.1347	C	-0.5588	-2.5582	-0.3026	H	-0.6683	-0.0876	2.9005
C	3.0880	-3.1320	0.2193	C	-2.0345	-0.0429	1.2290	C	5.4477	0.3334	1.4587
H	3.7411	-2.9927	-0.6528	C	4.2566	-0.3296	-0.9787	H	5.9053	0.5861	2.4121
H	3.0332	-4.2056	0.4265	H	3.7963	-0.5791	-1.9311	C	-1.9565	-3.0423	-0.5137
H	3.5928	-2.6436	1.0606	C	-4.7249	0.0351	-1.3649	H	-2.6652	-2.6101	0.1992
C	-1.9517	3.0801	-0.2570	H	-5.7010	0.0096	-0.8834	H	-1.9746	-4.1293	-0.4100
H	-2.4744	2.7334	-1.1554	C	0.6056	3.3966	-0.2010	H	-2.3263	-2.7891	-1.5175
H	-1.9376	4.1717	-0.2642	H	0.5852	4.4780	-0.2683	C	6.2502	0.0074	0.3597
H	-2.5481	2.7484	0.6032	C	-4.6403	0.0974	-2.7590	H	7.3332	0.0114	0.4525
C	-2.2690	0.0534	-1.2392	H	-5.5518	0.1159	-3.3516	B	-1.0952	0.0072	-0.1091
C	-3.4040	-0.0408	0.8571	C	3.1468	3.0995	-0.1960	C	5.6490	-0.3232	-0.8600
C	1.2444	1.2270	-0.0870	H	3.6664	2.9897	0.7656	H	6.2642	-0.5712	-1.7216
C	0.5716	-3.3906	-0.1604								

Table S18. Atomic coordinates for optimised structure of **Bf-A3** in its T_1 triplet excited state.

	x	*y*	*z*		*x*	*y*	*z*		*x*	*y*	*z*
N	8.7033	2.5514	1.2673	H	11.4500	1.4563	2.7749	H	3.2484	5.2840	3.0709
N	6.4717	3.5050	0.6434	C	9.5963	5.6955	-2.1569	H	4.7910	5.6576	3.8625
C	9.5681	2.4210	3.4026	H	10.0077	6.7006	-2.0924	C	4.9271	4.3989	2.1062
C	8.5432	3.1161	-2.3324	C	5.3479	3.9245	-0.0750	C	6.7182	-0.6643	-1.5837

C	8.4262	2.8510	2.5797	C	7.1816	-0.4266	-2.8834	H	6.2452	-1.6141	-1.3432
C	6.9455	3.7886	4.4145	H	7.0675	-1.1912	-3.6486	C	6.3203	2.8536	5.2559
C	7.2116	3.4711	2.9764	C	7.7928	0.7938	-3.2041	H	6.0273	1.8874	4.8515
C	9.0231	5.0933	-1.0238	H	8.1492	0.9683	-4.2176	C	6.8645	0.3224	-0.5938
H	8.9976	5.6464	-0.0847	C	9.9658	1.9528	1.2158	H	6.4984	0.1222	0.4134
C	9.7552	2.5233	4.8850	C	7.4697	1.5449	-0.8909	C	6.0714	3.1482	6.5998
H	9.7129	3.5601	5.2380	C	7.3154	5.0302	4.9575	H	5.5862	2.4103	7.2340
H	10.7320	2.1105	5.1580	H	7.8006	5.7656	4.3197	C	10.6328	1.4765	-0.0270
H	8.9864	1.9725	5.4391	C	9.1117	3.7065	-3.4666	H	10.0539	0.6882	-0.5224
C	5.1858	3.8043	-1.5500	H	9.1503	3.1764	-4.4164	H	11.6197	1.0766	0.2226
H	5.9804	4.3300	-2.0916	C	4.4128	4.4672	0.8232	H	10.7585	2.2839	-0.7573
H	4.2231	4.2320	-1.8439	H	3.4475	4.8688	0.5390	C	6.4456	4.3891	7.1265
H	5.2163	2.7589	-1.8782	C	9.6389	5.0022	-3.3726	H	6.2531	4.6203	8.1712
C	8.4918	3.8040	-1.0942	H	10.0829	5.4697	-4.2486	B	7.7735	2.8595	0.0324
C	7.9335	1.7692	-2.2109	C	4.2273	4.8749	3.3417	C	7.0690	5.3305	6.3006
C	6.2546	3.7777	1.9728	H	4.0703	4.0678	4.0664	H	7.3635	6.2975	6.7010
C	10.4849	1.8774	2.5198								

Table S19. Atomic coordinates for optimised structure of **Bf-B1** in its ground state.

	x	*y*	*z*		*x*	*y*	*z*		*x*	*y*	*z*
S	4.3829	7.9221	16.2042	C	6.6859	5.0538	11.2454	C	7.1074	11.5838	13.2764
O	5.9344	8.3991	11.9897	C	3.4105	6.6886	10.7263	H	7.6084	12.4761	12.9104
N	4.9517	7.0377	13.8396	H	2.8558	7.5089	11.1810	C	2.8681	4.1434	16.0347
C	5.1130	8.0983	14.6289	C	6.8437	10.5417	12.3960	H	2.3261	3.4154	16.6308
C	4.2593	5.9914	14.4663	H	7.1250	10.6020	11.3488	C	6.7407	11.4968	14.6332
C	3.8624	6.3029	15.7819	C	3.2699	3.8177	14.7270	H	6.9582	12.3130	15.3147
C	3.1623	5.3926	16.5775	H	3.0366	2.8366	14.3240	B	5.6073	6.9861	12.3649
H	2.8612	5.6500	17.5880	C	2.8408	5.9877	9.6503	C	7.6519	4.0909	10.9344
C	4.6713	6.3378	11.2111	H	1.8558	6.2627	9.2799	H	7.4964	3.3808	10.1247
C	6.2050	9.3731	12.8529	C	6.0990	10.3569	15.0923	C	8.8359	4.0433	11.6831
C	6.8848	5.9793	12.3015	H	5.8153	10.2857	16.1391	H	9.5929	3.2984	11.4489
C	3.9640	4.7275	13.9337	C	3.5405	4.9330	9.0519	C	4.8115	4.5699	9.5184
H	4.2656	4.4662	12.9280	H	3.0974	4.3926	8.2185	H	5.3476	3.7513	9.0426
C	5.3699	5.2714	10.5926	C	8.0701	5.9096	13.0377	C	9.0464	4.9467	12.7313
C	5.8177	9.2862	14.2157	H	8.2512	6.6018	13.8595	H	9.9670	4.9008	13.3089

Table S20. Atomic coordinates for optimised structure of **Bf-B1** in its S_1 singlet excited state.

	x	*y*	*z*		*x*	*y*	*z*		*x*	*y*	*z*
S	4.2053	7.9827	16.1822	C	6.6356	4.9364	11.2420	C	6.9070	11.6892	13.1531
O	6.3421	8.2183	12.1709	C	3.6331	7.0091	10.7554	H	7.3862	12.5795	12.7571
N	5.0645	6.9742	13.9129	H	3.1806	7.8784	11.2269	C	3.0359	4.0557	16.1266
C	5.0519	8.1549	14.6392	C	6.9209	10.4945	12.4126	H	2.5038	3.3250	16.7288
C	4.4183	5.9338	14.5513	H	7.4056	10.4409	11.4413	C	6.2699	11.7162	14.4018
C	3.8766	6.2849	15.8140	C	3.5649	3.6938	14.8813	H	6.2515	12.6337	14.9842

C	3.1918	5.3668	16.6018	H	3.4407	2.6768	14.5189	B	5.7519	6.9163	12.5301
H	2.7855	5.6587	17.5659	C	3.0044	6.4114	9.6582	C	7.4979	3.8804	10.8835
C	4.8033	6.4516	11.2704	H	2.0613	6.8074	9.2913	H	7.2747	3.2390	10.0356
C	6.3091	9.3461	12.9111	C	5.6534	10.5714	14.9128	C	8.6610	3.6902	11.6181
C	6.9327	5.7860	12.3546	H	5.1657	10.6158	15.8833	H	9.3414	2.8844	11.3581
C	4.2538	4.6141	14.0865	C	3.5707	5.2808	9.0274	C	4.7610	4.7355	9.4906
H	4.6533	4.3098	13.1278	H	3.0720	4.8434	8.1672	H	5.2073	3.8846	8.9837
C	5.3874	5.3264	10.6066	C	8.1308	5.6033	13.0450	C	8.9769	4.5450	12.6981
C	5.6527	9.3524	14.1875	H	8.3921	6.2496	13.8797	H	9.8818	4.3635	13.2718

Table S21. Atomic coordinates for optimised structure of **Bf-B1** in its S_2 singlet excited state.

	x	*y*	*z*		*x*	*y*	*z*		*x*	*y*	*z*
S	4.1946	8.0211	16.1515	C	6.6910	4.9830	11.1780	C	6.9022	11.7006	13.1016
O	6.3949	8.2167	12.1847	C	3.5431	6.8686	10.9151	H	7.3777	12.5911	12.7017
N	5.0986	6.9819	13.9046	H	3.0353	7.6464	11.4811	C	3.0501	4.0906	16.1473
C	5.0604	8.1764	14.6211	C	6.9444	10.4988	12.3830	H	2.5137	3.3678	16.7550
C	4.4465	5.9461	14.5548	H	7.4471	10.4314	11.4221	C	6.2395	11.7421	14.3420
C	3.8859	6.3157	15.8019	C	3.5981	3.7093	14.9141	H	6.2022	12.6709	14.9051
C	3.1939	5.4088	16.5991	H	3.4835	2.6856	14.5682	B	5.7936	6.9279	12.5259
H	2.7739	5.7180	17.5518	C	2.9431	6.3528	9.7516	C	7.5817	3.9708	10.7782
C	4.8041	6.4226	11.2864	H	1.9844	6.7443	9.4212	H	7.3970	3.3897	9.8784
C	6.3339	9.3497	12.8932	C	5.6256	10.6037	14.8650	C	8.6883	3.6894	11.5787
C	6.9265	5.7259	12.3645	H	5.1204	10.6619	15.8253	H	9.3837	2.9076	11.2857
C	4.2928	4.6177	14.1136	C	3.5878	5.3613	8.9937	C	4.8334	4.8713	9.3854
H	4.7072	4.2970	13.1662	H	3.1053	4.9649	8.1046	H	5.3074	4.0719	8.8217
C	5.4384	5.3936	10.5425	C	8.0061	5.4056	13.1768	C	8.9020	4.3993	12.7719
C	5.6513	9.3710	14.1624	H	8.1959	5.9544	14.0965	H	9.7818	4.1869	13.3739

Table S22. Atomic coordinates for optimised structure of **Bf-B1** in its T_1 triplet excited state.

	x	*y*	*z*		*x*	*y*	*z*		*x*	*y*	*z*
S	4.2195	7.9894	16.1832	C	6.6549	4.9532	11.2364	C	6.9109	11.6819	13.1168
O	6.3484	8.2368	12.1522	C	3.5746	6.9541	10.8143	H	7.3814	12.5812	12.7336
N	5.0768	6.9750	13.8958	H	3.1059	7.7977	11.3188	C	3.0654	4.0723	16.1150
C	5.0619	8.1615	14.6333	C	6.9275	10.5054	12.3787	H	2.5371	3.3381	16.7157
C	4.4361	5.9449	14.5390	H	7.4067	10.4500	11.4055	C	6.2682	11.6971	14.3860
C	3.8982	6.3008	15.8076	C	3.5922	3.7064	14.8619	H	6.2550	12.6174	14.9638
C	3.2169	5.3777	16.5956	H	3.4655	2.6886	14.5045	B	5.7631	6.9082	12.4977
H	2.8121	5.6642	17.5614	C	2.9432	6.3802	9.6984	C	7.5260	3.9192	10.8756
C	4.7865	6.4391	11.2727	H	1.9929	6.7761	9.3480	H	7.3158	3.2845	10.0174
C	6.3171	9.3427	12.8782	C	5.6581	10.5696	14.9066	C	8.6850	3.7055	11.6331
C	6.9334	5.7751	12.3559	H	5.1775	10.6188	15.8793	H	9.3670	2.9040	11.3595
C	4.2736	4.6219	14.0689	C	3.5342	5.2971	9.0366	C	4.7561	4.7745	9.4799
H	4.6743	4.3281	13.1076	H	3.0426	4.8578	8.1719	H	5.2073	3.9346	8.9561
C	5.3803	5.3470	10.5939	C	8.0951	5.5555	13.0950	C	8.9704	4.5164	12.7382
C	5.6529	9.3419	14.1807	H	8.3299	6.1806	13.9552	H	9.8711	4.3397	13.3215

Table S23. Atomic coordinates for optimised structure of **Bf-B2** in its ground state.

	x	*y*	*z*		*x*	*y*	*z*		*x*	*y*	*z*
O	4.8063	2.4916	16.2008	C	6.9002	-0.1788	11.5191	H	5.4286	-1.3876	9.3468
O	6.3431	3.2102	12.4669	C	7.0910	5.3809	13.0340	C	3.0802	0.9958	10.0250
N	5.2807	1.7468	14.1602	H	7.4469	5.5087	12.0161	H	2.1058	1.3336	9.6792
C	4.5940	0.7212	14.8239	C	4.9663	1.2232	11.5425	C	7.2256	6.3928	13.9778
C	5.3805	2.7556	15.0122	C	3.6258	0.4782	17.0672	H	7.6992	7.3269	13.6871
C	4.3029	1.2035	16.1021	H	3.4142	0.8835	18.0507	C	8.3778	0.5888	13.2769
C	6.0160	4.0012	14.7200	C	6.1582	5.0381	15.6652	H	8.6167	1.2655	14.0970
C	5.5823	0.1194	10.9025	H	5.7901	4.8903	16.6760	C	6.7634	6.2305	15.2988
C	6.4869	4.1590	13.3875	B	5.9459	1.7784	12.7077	H	6.8787	7.0312	16.0223
C	3.2358	-0.8111	16.6862	C	3.7001	-0.0950	9.4039	C	7.8063	-1.1867	11.1722
H	2.7007	-1.4324	17.3978	H	3.2056	-0.6009	8.5778	H	7.5926	-1.8795	10.3609
C	4.2054	-0.5652	14.4464	C	3.7159	1.6511	11.0929	C	9.2951	-0.4186	12.9336
H	4.4211	-0.9589	13.4601	H	3.2219	2.4986	11.5673	H	10.2296	-0.5156	13.4818
C	3.5214	-1.3188	15.4044	C	7.1763	0.7231	12.5775	C	9.0075	-1.3010	11.8857
H	3.2008	-2.3250	15.1517	C	4.9563	-0.5397	9.8388	H	9.7186	-2.0808	11.6230

Table S24. Atomic coordinates for optimised structure of **Bf-B2** in its S_1 singlet excited state.

	x	*y*	*z*		*x*	*y*	*z*		*x*	*y*	*z*
O	4.8018	2.4604	16.2654	C	6.8481	-0.1927	11.5383	H	5.4260	-1.4326	9.3807
O	6.3268	3.1759	12.4928	C	7.1001	5.3492	13.0385	C	3.1477	1.0515	9.9508
N	5.2190	1.7321	14.1620	H	7.4626	5.4441	12.0180	H	2.1887	1.3957	9.5731
C	4.5756	0.7093	14.8356	C	4.9610	1.2480	11.5361	C	7.2522	6.4044	13.9620
C	5.3599	2.7791	15.0416	C	3.6698	0.4227	17.1017	H	7.7379	7.3257	13.6547
C	4.3132	1.1730	16.1388	H	3.4841	0.8130	18.0972	C	8.3803	0.6679	13.2235
C	5.9812	3.9963	14.7521	C	6.1480	5.0714	15.6707	H	8.6466	1.3764	14.0040
C	5.5743	0.1054	10.9214	H	5.7785	4.9606	16.6859	C	6.7731	6.2519	15.2724
C	6.4797	4.1639	13.4119	B	5.8879	1.7932	12.7704	H	6.8879	7.0609	15.9899
C	3.2709	-0.8769	16.7223	C	3.7446	-0.0936	9.3710	C	7.7469	-1.2333	11.2088
H	2.7612	-1.5071	17.4451	H	3.2489	-0.5920	8.5433	H	7.5190	-1.9395	10.4160
C	4.1852	-0.5829	14.4661	C	3.7640	1.7279	11.0098	C	9.2625	-0.3694	12.9039
H	4.3781	-0.9745	13.4738	H	3.2831	2.6028	11.4406	H	10.2002	-0.4682	13.4439
C	3.5269	-1.3612	15.4346	C	7.1447	0.7473	12.5834	C	8.9455	-1.3152	11.8973
H	3.2120	-2.3675	15.1718	C	4.9557	-0.5710	9.8451	H	9.6572	-2.1006	11.6615

Table S25. Atomic coordinates for optimised structure of **Bf-B2** in its S_2 singlet excited state.

	x	*y*	*z*		*x*	*y*	*z*		*x*	*y*	*z*
O	4.8018	2.4604	16.2654	C	6.8481	-0.1927	11.5383	H	5.4260	-1.4326	9.3807
O	6.3268	3.1759	12.4928	C	7.1001	5.3492	13.0385	C	3.1477	1.0515	9.9508
N	5.2190	1.7321	14.1620	H	7.4626	5.4441	12.0180	H	2.1887	1.3957	9.5731
C	4.5756	0.7093	14.8356	C	4.9610	1.2480	11.5361	C	7.2522	6.4044	13.9620
C	5.3599	2.7791	15.0416	C	3.6698	0.4227	17.1017	H	7.7379	7.3257	13.6547

C	4.3132	1.1730	16.1388	H	3.4841	0.8130	18.0972	C	8.3803	0.6679	13.2235
C	5.9812	3.9963	14.7521	C	6.1480	5.0714	15.6707	H	8.6466	1.3764	14.0040
C	5.5743	0.1054	10.9214	H	5.7785	4.9606	16.6859	C	6.7731	6.2519	15.2724
C	6.4797	4.1639	13.4119	B	5.8879	1.7932	12.7704	H	6.8879	7.0609	15.9899
C	3.2709	-0.8769	16.7223	C	3.7446	-0.0936	9.3710	C	7.7469	-1.2333	11.2088
H	2.7612	-1.5071	17.4451	H	3.2489	-0.5920	8.5433	H	7.5190	-1.9395	10.4160
C	4.1852	-0.5829	14.4661	C	3.7640	1.7279	11.0098	C	9.2625	-0.3694	12.9039
H	4.3781	-0.9745	13.4738	H	3.2831	2.6028	11.4406	H	10.2002	-0.4682	13.4439
C	3.5269	-1.3612	15.4346	C	7.1447	0.7473	12.5834	C	8.9455	-1.3152	11.8973
H	3.2120	-2.3675	15.1718	C	4.9557	-0.5710	9.8451	H	9.6572	-2.1006	11.6615

Table S26. Atomic coordinates for optimised structure of **Bf-B2** in its T_1 triplet excited state.

	x	*y*	*z*		*x*	*y*	*z*		*x*	*y*	*z*
O	4.6863	2.5200	16.2161	C	6.8751	-0.2356	11.5141	H	5.3405	-1.2250	9.2780
O	6.6013	3.0847	12.5853	C	7.1329	5.3457	12.9991	C	3.1613	1.2814	10.0694
N	5.3728	1.6824	14.2147	H	7.6116	5.3848	12.0247	H	2.2148	1.7038	9.7401
C	4.7293	0.6745	14.8880	C	5.0434	1.2928	11.5980	C	7.0926	6.4594	13.8342
C	5.3408	2.7999	15.0318	C	3.6198	0.4608	17.0711	H	7.5430	7.3965	13.5247
C	4.3058	1.2010	16.1292	H	3.3057	0.8892	18.0168	C	8.3871	0.3481	13.3207
C	5.8906	4.0252	14.7063	C	5.8664	5.1848	15.5378	H	8.6603	0.9711	14.1710
C	5.5907	0.1844	10.9079	H	5.3849	5.1319	16.5087	C	6.4527	6.3597	15.1040
C	6.5517	4.1350	13.3977	B	6.0455	1.7193	12.8176	H	6.4241	7.2323	15.7516
C	3.3532	-0.8782	16.7320	C	3.7070	0.1826	9.3954	C	7.7162	-1.2849	11.1278
H	2.8163	-1.5040	17.4382	H	3.1837	-0.2439	8.5430	H	7.4658	-1.9191	10.2801
C	4.4590	-0.6639	14.5565	C	3.8332	1.8375	11.1709	C	9.2345	-0.7044	12.9360
H	4.7759	-1.0852	13.6097	H	3.3982	2.6924	11.6866	H	10.1531	-0.8919	13.4871
C	3.7661	-1.4230	15.5006	C	7.2060	0.5854	12.6190	C	8.8978	-1.5142	11.8451
H	3.5393	-2.4617	15.2797	C	4.9247	-0.3725	9.8106	H	9.5572	-2.3272	11.5506

Table S27. Atomic coordinates for optimised structure of **Bf-B3** in its ground state.

	x	*y*	*z*		*x*	*y*	*z*		*x*	*y*	*z*
B	1.3621	3.0610	7.0782	H	-1.0054	7.4826	4.9889	H	0.1058	0.7790	5.4641
O	1.6901	2.1440	8.2168	C	-2.6144	6.6442	6.1605	C	1.2754	0.9420	3.6618
N	-0.1262	3.5531	7.3456	H	-3.3100	7.4319	5.8864	H	0.7751	0.1076	3.1751
N	-2.2088	3.4913	8.1169	C	-3.0416	5.6226	7.0077	C	2.2904	1.6333	2.9902
C	0.7886	1.3304	8.7726	H	-4.0527	5.5930	7.4000	H	2.5762	1.3352	1.9840
C	1.2435	0.1806	9.4432	C	-2.1052	4.6358	7.3216	C	2.9398	2.7111	3.6076
H	2.3128	-0.0082	9.4697	C	-3.3842	3.1821	8.8900	H	3.7247	3.2447	3.0752
C	0.3407	-0.6943	10.0367	C	-3.4238	3.5204	10.2454	C	2.5637	3.0843	4.9026
H	0.7097	-1.5839	10.5406	H	-2.5610	3.9850	10.7134	C	3.1156	4.1864	5.7308
C	-1.0398	-0.4444	9.9755	C	-4.5787	3.2457	10.9820	C	4.1379	5.0843	5.4034
H	-1.7457	-1.1385	10.4211	H	-4.6148	3.4988	12.0377	H	4.6409	5.0360	4.4397
C	-1.5034	0.7003	9.3389	C	-5.6841	2.6537	10.3615	C	4.5175	6.0583	6.3372
H	-2.5694	0.8816	9.2936	H	-6.5809	2.4434	10.9374	H	5.3106	6.7609	6.0915
C	-0.6063	1.6145	8.7459	C	-5.6381	2.3352	9.0001	C	3.8840	6.1290	7.5838

C	-0.9962	2.8542	8.0970	H	-6.4964	1.8790	8.5155	H	4.1871	6.8870	8.3027
C	-0.7884	4.6641	6.8322	C	-4.4851	2.6004	8.2563	C	2.8587	5.2232	7.9032
C	-0.3670	5.6898	5.9765	H	-4.4330	2.3520	7.2004	H	2.3737	5.2902	8.8769
H	0.6434	5.7192	5.5871	C	1.5387	2.3946	5.5990	C	2.4608	4.2496	6.9854
C	-1.2996	6.6732	5.6508	C	0.9019	1.3269	4.9607				

Table S28. Atomic coordinates for optimised structure of **Bf-B3** in its S_1 singlet excited state.

	x	*y*	*z*		*x*	*y*	*z*		*x*	*y*	*z*
B	1.2001	2.9010	6.9024	H	-1.2932	6.8376	4.2275	H	0.3952	0.7437	4.9232
O	1.5397	1.7944	7.8089	C	-2.8473	6.1563	5.5564	C	1.6845	1.2651	3.2752
N	-0.2602	3.3103	7.0897	H	-3.5671	6.8906	5.2056	H	1.2893	0.4911	2.6225
N	-2.3468	3.3145	7.9570	C	-3.2299	5.2489	6.5616	C	2.6963	2.1234	2.7993
C	0.6785	1.0992	8.5769	H	-4.2265	5.2763	6.9892	H	3.0890	1.9830	1.7959
C	1.1616	-0.0273	9.2410	C	-2.2839	4.3190	6.9817	C	3.2043	3.1380	3.6065
H	2.2086	-0.2872	9.1095	C	-3.3555	3.2837	8.9686	H	4.0053	3.7763	3.2431
C	0.3120	-0.8055	10.0452	C	-3.0299	3.5826	10.2988	C	2.6848	3.2976	4.9032
H	0.6951	-1.6818	10.5590	H	-2.0024	3.8193	10.5599	C	3.0796	4.2663	5.9236
C	-1.0368	-0.4368	10.1595	C	-4.0281	3.5678	11.2751	C	4.0728	5.2581	5.8426
H	-1.7136	-1.0371	10.7626	H	-3.7719	3.7948	12.3065	H	4.6658	5.3829	4.9405
C	-1.5389	0.6926	9.5074	C	-5.3518	3.2733	10.9282	C	4.3083	6.0655	6.9528
H	-2.5887	0.9349	9.6128	H	-6.1263	3.2659	11.6901	H	5.0739	6.8354	6.9091
C	-0.7007	1.5180	8.7075	C	-5.6733	2.9873	9.5968	C	3.5696	5.8866	8.1388
C	-1.0910	2.6886	8.0160	H	-6.6980	2.7544	9.3200	H	3.7556	6.5388	8.9882
C	-0.9744	4.2948	6.4431	C	-4.6778	2.9863	8.6162	C	2.5888	4.8906	8.2237
C	-0.6001	5.2001	5.4399	H	-4.9179	2.7434	7.5852	H	2.0257	4.7668	9.1463
H	0.3975	5.1907	5.0166	C	1.6360	2.4559	5.3766	C	2.3072	4.1008	7.1094
C	-1.5597	6.1268	5.0051	C	1.1761	1.4140	4.5706				

Table S29. Atomic coordinates for optimised structure of **Bf-B3** in its S_2 singlet excited state.

	x	*y*	*z*		*x*	*y*	*z*		*x*	*y*	*z*
B	1.2001	2.9010	6.9024	H	-1.2932	6.8376	4.2275	H	0.3952	0.7437	4.9232
O	1.5397	1.7944	7.8089	C	-2.8473	6.1563	5.5564	C	1.6845	1.2651	3.2752
N	-0.2602	3.3103	7.0897	H	-3.5671	6.8906	5.2056	H	1.2893	0.4911	2.6225
N	-2.3468	3.3145	7.9570	C	-3.2299	5.2489	6.5616	C	2.6963	2.1234	2.7993
C	0.6785	1.0992	8.5769	H	-4.2265	5.2763	6.9892	H	3.0890	1.9830	1.7959
C	1.1616	-0.0273	9.2410	C	-2.2839	4.3190	6.9817	C	3.2043	3.1380	3.6065
H	2.2086	-0.2872	9.1095	C	-3.3555	3.2837	8.9686	H	4.0053	3.7763	3.2431
C	0.3120	-0.8055	10.0452	C	-3.0299	3.5826	10.2988	C	2.6848	3.2976	4.9032
H	0.6951	-1.6818	10.5590	H	-2.0024	3.8193	10.5599	C	3.0796	4.2663	5.9236
C	-1.0368	-0.4368	10.1595	C	-4.0281	3.5678	11.2751	C	4.0728	5.2581	5.8426
H	-1.7136	-1.0371	10.7626	H	-3.7719	3.7948	12.3065	H	4.6658	5.3829	4.9405
C	-1.5389	0.6926	9.5074	C	-5.3518	3.2733	10.9282	C	4.3083	6.0655	6.9528
H	-2.5887	0.9349	9.6128	H	-6.1263	3.2659	11.6901	H	5.0739	6.8354	6.9091
C	-0.7007	1.5180	8.7075	C	-5.6733	2.9873	9.5968	C	3.5696	5.8866	8.1388

C	-1.0910	2.6886	8.0160	H	-6.6980	2.7544	9.3200	H	3.7556	6.5388	8.9882
C	-0.9744	4.2948	6.4431	C	-4.6778	2.9863	8.6162	C	2.5888	4.8906	8.2237
C	-0.6001	5.2001	5.4399	H	-4.9179	2.7434	7.5852	H	2.0257	4.7668	9.1463
H	0.3975	5.1907	5.0166	C	1.6360	2.4559	5.3766	C	2.3072	4.1008	7.1094
C	-1.5597	6.1268	5.0051	C	1.1761	1.4140	4.5706				

Table S30. Atomic coordinates for optimised structure of **Bf-B3** in its T_1 triplet excited state.

	x	*y*	*z*		*x*	*y*	*z*		*x*	*y*	*z*
B	1.2593	2.9221	6.9229	H	-1.2025	6.9284	4.3539	H	0.2183	0.8671	4.9341
O	1.5815	1.8109	7.8628	C	-2.7767	6.2205	5.6533	C	1.5369	1.2758	3.2743
N	-0.2124	3.3540	7.1464	H	-3.4863	6.9652	5.3047	H	1.0974	0.5097	2.6396
N	-2.3237	3.3403	7.9849	C	-3.1789	5.3010	6.6304	C	2.5955	2.0561	2.7944
C	0.7280	1.1243	8.6123	H	-4.1816	5.3205	7.0433	H	2.9765	1.8925	1.7891
C	1.1980	0.0032	9.3074	C	-2.2392	4.3593	7.0453	C	3.1688	3.0479	3.6015
H	2.2483	-0.2526	9.1981	C	-3.3781	3.2561	8.9522	H	3.9917	3.6480	3.2189
C	0.3429	-0.7608	10.0963	C	-3.1390	3.6268	10.2810	C	2.6713	3.2497	4.8938
H	0.7089	-1.6317	10.6296	H	-2.1483	3.9581	10.5786	C	3.1179	4.2351	5.9063
C	-1.0334	-0.3930	10.1762	C	-4.1791	3.5602	11.2106	C	4.1399	5.1848	5.7994
H	-1.7113	-1.0025	10.7685	H	-3.9941	3.8413	12.2436	H	4.7321	5.2698	4.8907
C	-1.5369	0.7099	9.5184	C	-5.4544	3.1431	10.8127	C	4.4001	6.0346	6.8828
H	-2.5907	0.9411	9.5998	H	-6.2618	3.0956	11.5382	H	5.1926	6.7755	6.8083
C	-0.6840	1.5480	8.7222	C	-5.6884	2.7874	9.4803	C	3.6470	5.9350	8.0587
C	-1.0674	2.7046	8.0465	H	-6.6762	2.4616	9.1666	H	3.8554	6.6005	8.8933
C	-0.9171	4.3443	6.5209	C	-4.6499	2.8388	8.5463	C	2.6225	4.9790	8.1603
C	-0.5267	5.2705	5.5354	H	-4.8172	2.5477	7.5134	H	2.0441	4.9121	9.0808
H	0.4765	5.2612	5.1267	C	1.5980	2.4685	5.3867	C	2.3481	4.1277	7.0901
C	-1.4740	6.2005	5.1130	C	1.0416	1.4821	4.5729				

Table S31. Atomic coordinates for optimised structure of **Bf-C** in its ground state.

	x	*y*	*z*		*x*	*y*	*z*		*x*	*y*	*z*
O	1.1652	6.6367	1.5964	H	-2.8022	7.5898	-0.4366	C	2.4260	4.4140	1.6015
N	0.2044	4.8882	0.1169	C	-1.4846	3.8606	4.3970	C	-0.5977	2.9675	5.0084
C	-0.7210	5.7896	-0.3266	H	-2.4909	3.9804	4.7921	H	-0.9155	2.3950	5.8768
C	-0.8974	7.0333	0.4335	C	3.5959	4.5378	0.8488	C	-2.0922	9.1113	0.8865
C	0.4140	3.7348	-0.5565	H	3.6323	5.2176	-0.0019	H	-2.9543	9.7511	0.7236
H	1.1652	3.0786	-0.1351	C	-1.0780	4.6002	3.2735	C	4.7378	3.7880	1.1765
C	0.2131	4.4608	2.7553	H	-1.7874	5.2853	2.8118	H	5.6452	3.8886	0.5853
C	0.1070	7.4258	1.3524	C	3.5478	2.7746	3.0384	C	-0.2826	3.4081	-1.7059
C	0.6997	2.8057	4.5043	H	3.5399	2.0930	3.8864	H	-0.0830	2.4687	-2.2088
H	1.3819	2.1069	4.9843	C	2.4149	3.5253	2.7043	C	4.7097	2.9099	2.2665
C	0.0253	8.6762	1.9879	C	-1.0646	9.5095	1.7569	H	5.5948	2.3303	2.5185
H	0.8209	8.9626	2.6698	H	-1.1244	10.4681	2.2658	C	-1.4333	5.5099	-1.5057
C	1.0972	3.5481	3.3871	C	-1.2247	4.3211	-2.1909	H	-2.1374	6.2369	-1.8903
C	-2.0004	7.8886	0.2311	H	-1.7818	4.1121	-3.0991	B	0.9963	5.1649	1.5066

Table S32. Atomic coordinates for optimised structure of **Bf-C** in its S_1 singlet excited state.

	x	*y*	*z*		*x*	*y*	*z*		*x*	*y*	*z*
O	0.7004	6.4812	2.0356	H	-2.1369	8.1181	-1.1473	C	2.2732	4.5358	1.7096
N	0.0211	4.8509	0.2791	C	-1.4367	3.2952	4.5036	C	-0.4462	2.4137	4.9852
C	-0.7901	5.8408	-0.3302	H	-2.4372	3.2567	4.9267	H	-0.6874	1.7281	5.7929
C	-0.7966	7.1727	0.2641	C	3.4069	4.9267	0.9960	C	-1.4997	9.5265	0.3264
C	0.0694	3.5934	-0.2935	H	3.3407	5.7018	0.2354	H	-2.0818	10.3286	-0.1193
H	0.7086	2.8794	0.2129	C	-1.1458	4.2018	3.4787	C	4.6288	4.2820	1.2224
C	0.1188	4.1939	2.8880	H	-1.9216	4.8743	3.1190	H	5.5017	4.5499	0.6328
C	-0.0246	7.4417	1.4379	C	3.6282	2.8955	2.9538	C	-0.6294	3.2549	-1.4179
C	0.8369	2.4261	4.4462	H	3.7249	2.1357	3.7248	H	-0.5368	2.2496	-1.8162
H	1.6035	1.7666	4.8444	C	2.3982	3.5354	2.7178	C	4.7361	3.2717	2.1992
C	0.0057	8.7181	2.0230	C	-0.7279	9.7684	1.4726	H	5.6952	2.7900	2.3686
H	0.6133	8.8556	2.9132	H	-0.7015	10.7542	1.9270	C	-1.5143	5.5010	-1.4766
C	1.1268	3.3269	3.4055	C	-1.4643	4.2305	-2.0449	H	-2.1345	6.2583	-1.9432
C	-1.5294	8.2567	-0.2594	H	-2.0379	3.9943	-2.9338	B	0.7232	5.0723	1.6266

Table S33. Atomic coordinates for optimised structure of **Bf-C** in its S_2 singlet excited state.

	x	*y*	*z*		*x*	*y*	*z*		*x*	*y*	*z*
O	1.0404	6.5888	1.7627	H	-2.6229	7.8658	-0.6378	C	2.4189	4.4367	1.6153
N	0.2213	4.9123	0.1355	C	-1.5117	3.5978	4.2682	C	-0.5756	2.7951	4.9406
C	-0.7941	5.8133	-0.2871	H	-2.5344	3.6531	4.6330	H	-0.8885	2.2058	5.7984
C	-0.8772	7.0975	0.3866	C	3.5732	4.5820	0.8492	C	-1.8575	9.3237	0.7219
C	0.3584	3.7183	-0.5390	H	3.5808	5.2367	-0.0200	H	-2.6275	10.0434	0.4580
H	1.1530	3.0752	-0.1794	C	-1.1305	4.3533	3.1442	C	4.7505	3.9083	1.2135
C	0.1939	4.3402	2.7238	H	-1.8702	4.9777	2.6469	H	5.6594	4.0535	0.6351
C	0.0919	7.4485	1.3925	C	3.6188	2.8794	3.1007	C	-0.4368	3.3516	-1.5888
C	0.7474	2.7369	4.5016	H	3.6332	2.2068	3.9545	H	-0.2601	2.4006	-2.0805
H	1.4587	2.0829	4.9998	C	2.4439	3.5631	2.7387	C	4.7690	3.0598	2.3345
C	0.0841	8.7134	2.0126	C	-0.8818	9.6574	1.6799	H	5.6850	2.5393	2.6010
H	0.8483	8.9173	2.7573	H	-0.8876	10.6318	2.1589	C	-1.6241	5.4409	-1.3552
C	1.1316	3.5001	3.3851	C	-1.4791	4.2318	-2.0201	H	-2.3941	6.1310	-1.6819
C	-1.8525	8.0736	0.0967	H	-2.1309	3.9684	-2.8455	B	0.9561	5.1419	1.4754

Table S34. Atomic coordinates for optimised structure of **Bf-C** in its T_1 triplet excited state.

	x	*y*	*z*		*x*	*y*	*z*		*x*	*y*	*z*
O	0.7445	6.5115	2.0244	H	-2.2115	8.0979	-1.0967	C	2.2918	4.5104	1.6933
N	0.0359	4.8593	0.2852	C	-1.4713	3.2857	4.4655	C	-0.4748	2.4615	5.0021
C	-0.7845	5.8590	-0.3070	H	-2.4789	3.2537	4.8735	H	-0.7107	1.7938	5.8273
C	-0.8060	7.1522	0.2764	C	3.4137	4.8345	0.9297	C	-1.5433	9.4900	0.3618
C	0.1039	3.6334	-0.2936	H	3.3432	5.5709	0.1304	H	-2.1484	10.2869	-0.0623
H	0.7523	2.9215	0.2050	C	-1.1719	4.1519	3.4007	C	4.6453	4.2076	1.1812
C	0.1167	4.1880	2.8690	H	-1.9569	4.7883	2.9942	H	5.5166	4.4583	0.5806

C	0.0047	7.4517	1.4619	C	3.6386	2.9247	2.9846	C	-0.5915	3.2899	-1.4306
C	0.8266	2.4924	4.4840	H	3.7351	2.1877	3.7789	H	-0.4825	2.2924	-1.8411
H	1.5944	1.8508	4.9111	C	2.4140	3.5517	2.7285	C	4.7537	3.2582	2.2046
C	0.0241	8.7370	2.0402	C	-0.7358	9.7624	1.5060	H	5.7092	2.7760	2.3967
H	0.6508	8.8847	2.9150	H	-0.7256	10.7538	1.9462	C	-1.5242	5.5109	-1.4774
C	1.1202	3.3560	3.4226	C	-1.4447	4.2633	-2.0415	H	-2.1611	6.2603	-1.9317
C	-1.5800	8.2408	-0.2279	H	-2.0136	4.0193	-2.9324	B	0.7625	5.0746	1.6481

Table S35. Atomic coordinates for optimised structure of **Bf-D** in its ground state.

	x	*y*	*z*		*x*	*y*	*z*		*x*	*y*	*z*
O	9.0335	2.2470	3.3419	C	11.8101	2.3235	3.2499	C	11.0663	-1.1605	2.1066
N	11.2190	3.4541	3.5352	H	12.8927	2.2690	3.3530	H	11.5838	-2.0590	1.7857
C	7.4767	6.6074	4.2999	C	9.6798	1.1634	2.9239	C	11.7771	-0.0134	2.4231
H	6.9769	7.4068	3.7566	C	8.7525	4.5457	5.7241	H	12.8629	0.0001	2.3649
C	8.2356	5.6422	3.6287	H	9.2375	3.7520	6.2915	C	8.3760	6.0838	-0.1781
C	11.9657	5.7990	3.4985	C	12.0199	4.5224	4.0710	H	8.0202	6.7283	-0.9786
H	11.3243	5.9824	2.6438	C	7.9939	5.5110	6.4068	C	9.6610	-1.1515	2.2182
C	8.8865	4.6012	4.3354	H	7.8965	5.4634	7.4891	H	9.1006	-2.0518	1.9792
C	12.7571	6.8230	4.0226	C	7.3610	6.5371	5.6949	C	8.0200	6.3617	1.1488
H	12.7193	7.8101	3.5700	H	6.7740	7.2831	6.2258	H	7.3910	7.2216	1.3699
C	11.0976	1.1569	2.8302	C	12.8527	4.2778	5.1706	C	13.5972	6.5831	5.1157
C	8.4843	5.5257	2.1700	H	12.8646	3.2950	5.6335	H	14.2086	7.3848	5.5203
C	8.9711	-0.0151	2.6287	C	9.6512	4.1500	0.5554	C	9.1880	4.9833	-0.4765
H	7.8888	-0.0138	2.7178	H	10.2867	3.3019	0.3026	H	9.4609	4.7758	-1.5088
C	13.6418	5.3086	5.6878	C	9.3064	4.4050	1.8854	B	9.6298	3.6264	3.2796
H	14.2819	5.1138	6.5439								

Table S36. Atomic coordinates for optimised structure of **Bf-D** in its S_1 excited state.

	x	*y*	*z*		*x*	*y*	*z*		*x*	*y*	*z*
O	1.3054	-0.6011	1.2467	C	1.6748	1.6062	-0.4167	C	5.2014	0.5261	0.3024
N	0.3777	1.2094	-0.1917	H	1.8314	2.4911	-1.0247	H	6.2192	0.8207	0.0581
C	-3.0803	-2.1846	1.1525	C	2.5743	-0.2435	0.9269	C	4.1309	1.2305	-0.2392
H	-3.5869	-3.0377	0.7120	C	-1.7318	0.0296	2.3178	H	4.3114	2.0760	-0.9003
C	-1.8819	-1.6762	0.5899	H	-1.2398	0.8771	2.7883	C	-0.7614	-3.5689	-2.4796
C	-1.8969	1.6388	-1.0052	C	-0.6622	2.1027	-0.5025	H	-1.0253	-4.3921	-3.1363
H	-2.0442	0.5810	-1.1870	C	-2.9147	-0.4726	2.8693	C	4.9708	-0.5603	1.1644
C	-1.2001	-0.5428	1.1644	H	-3.3360	-0.0123	3.7587	H	5.8044	-1.1110	1.5913
C	-2.9260	2.5323	-1.3100	C	-3.5875	-1.5774	2.2851	C	-1.5662	-3.2490	-1.4030
H	-3.8645	2.1447	-1.6992	H	-4.5026	-1.9445	2.7396	H	-2.4663	-3.8211	-1.1998
C	2.7861	0.8789	0.0661	C	-0.4978	3.4961	-0.3332	C	-2.7533	3.9098	-1.1389
C	-1.1847	-2.1672	-0.5688	H	0.4313	3.8784	0.0788	H	-3.5547	4.6021	-1.3819
C	3.6529	-0.9399	1.4683	C	0.8028	-1.7617	-1.9069	C	0.4198	-2.8248	-2.7336
H	3.4522	-1.7812	2.1275	H	1.7121	-1.2123	-2.1356	H	1.0304	-3.0874	-3.5933
C	-1.5280	4.3814	-0.6504	C	0.0100	-1.3972	-0.8199	B	0.1749	-0.2459	0.3406
H	-1.3762	5.4475	-0.4990								

Table S37. Atomic coordinates for optimised structure of **Bf-D** in its T_1 triplet excited state.

	x	*y*	*z*		*x*	*y*	*z*		*x*	*y*	*z*
O	1.4175	-0.8721	0.9086	C	-1.4377	-0.0260	2.3933	H	5.8157	-1.0138	1.8896
N	0.4344	1.1369	-0.2504	H	-0.8421	0.7781	2.8228	C	-1.8048	-3.1360	-1.4816
C	-3.0001	-2.1078	1.3213	C	-0.6096	1.9851	-0.6320	H	-2.6970	-3.6978	-1.2130
H	-3.6074	-2.9141	0.9158	C	-2.5834	-0.4727	3.0716	C	-2.7255	3.6973	-1.3952
C	-1.8600	-1.6565	0.6469	H	-2.8709	-0.0135	4.0146	H	-3.5383	4.3543	-1.6910
C	-1.8236	1.4581	-1.1391	C	-3.3579	-1.5090	2.5362	C	0.0162	-2.7096	-3.0347
H	-1.9343	0.3905	-1.2704	H	-4.2428	-1.8530	3.0662	H	0.5259	-2.9347	-3.9686
C	-1.0748	-0.6028	1.1762	C	-0.4849	3.3946	-0.5151	B	0.2101	-0.3416	0.2068
C	-2.8578	2.3067	-1.5175	H	0.4100	3.8293	-0.0853	C	1.7332	1.6448	-0.2899
H	-3.7726	1.8802	-1.9197	C	0.5139	-1.7041	-2.1888	H	1.8924	2.5779	-0.8125
C	2.8214	0.9546	0.2539	H	1.4127	-1.1607	-2.4761	C	2.6287	-0.3330	0.8897
C	-1.3104	-2.1304	-0.6428	C	-0.1479	-1.4044	-0.9999	C	-1.1361	-3.4209	-2.6792
C	3.7042	-1.0256	1.4756	C	5.1947	0.7664	0.7972	H	-1.5152	-4.2002	-3.3359
H	3.5049	-1.9859	1.9426	H	6.1982	1.1821	0.7615	C	4.9815	-0.4849	1.4399
C	-1.5337	4.2298	-0.8860	C	4.1503	1.4652	0.2182	H	4.3313	2.4220	-0.2646
H	-1.4234	5.3041	-0.7671								

Table S38. Atomic coordinates for optimised structure of **Bf-E** in its ground state.

	x	*y*	*z*		*x*	*y*	*z*		*x*	*y*	*z*
O	-0.1411	3.6720	2.7941	C	3.2399	13.4159	1.4631	C	4.0851	13.3217	0.3528
N	0.8991	11.7304	3.7365	C	2.3973	14.6406	4.8493	H	5.0525	13.8195	0.3399
B	1.2547	13.0695	2.8931	H	1.5606	14.5427	5.5401	C	-0.3323	9.6688	5.1552
C	3.4687	14.1546	2.7310	C	2.4299	11.9289	-0.7575	H	-0.7867	8.8529	5.7106
C	-1.0386	12.8913	3.4095	H	2.1182	11.3528	-1.6257	C	-2.5384	10.9296	4.8196
C	2.3644	14.0051	3.6070	C	-2.4044	13.0576	3.5588	H	-3.1348	10.1959	5.3532
C	1.9805	12.7656	1.4797	H	-2.9152	13.9228	3.1481	C	1.5878	12.0258	0.3631
C	-0.4397	11.7401	3.9709	C	4.5803	14.9207	3.0967	H	0.6243	11.5175	0.3503
C	1.6365	10.7216	4.1908	H	5.4288	15.0348	2.4253	C	3.6720	12.5741	-0.7589
H	2.7009	10.7480	3.9847	C	4.5938	15.5508	4.3489	H	4.3218	12.4964	-1.6275
C	-1.1353	10.7385	4.6781	H	5.4532	16.1497	4.6414	C	-3.1281	12.0586	4.2698
C	3.5090	15.4144	5.2229	C	1.0328	9.6690	4.9103	H	-4.2003	12.1953	4.3836
H	3.5287	15.9089	6.1914	H	1.6593	8.8593	5.2681				

Table S39. Atomic coordinates for optimised structure of **Bf-E** in its S_1 singlet excited state.

	x	*y*	*z*		*x*	*y*	*z*		*x*	*y*	*z*
O	-0.0425	13.5780	2.8298	C	3.2389	13.4575	1.4426	C	4.0377	13.4436	0.2926
N	0.9448	11.6428	3.7769	C	2.4574	14.5604	4.8818	H	4.9560	14.0243	0.2407
B	1.3569	12.9074	2.9673	H	1.6662	14.3827	5.6087	C	-0.4394	9.6056	5.2128
C	3.4658	14.1930	2.7048	C	2.4487	11.9297	-0.7569	H	-0.9452	8.8199	5.7614
C	-0.9636	12.8450	3.4233	H	2.1513	11.3319	-1.6153	C	-2.5594	11.0133	4.7780
C	2.4236	13.9363	3.6340	C	-2.3499	13.1092	3.5206	H	-3.2051	10.3137	5.3017
C	2.0409	12.6986	1.5080	H	-2.7805	14.0015	3.0793	C	1.6505	11.9490	0.3975

C	-0.4130	11.6801	4.0013	C	4.5297	15.0422	3.0334	H	0.7328	11.3633	0.4225
C	1.6342	10.5532	4.2857	H	5.3289	15.2407	2.3227	C	3.6355	12.6751	-0.8058
H	2.7025	10.5250	4.1088	C	4.5519	15.6482	4.2947	H	4.2471	12.6579	-1.7046
C	-1.1534	10.7307	4.6878	H	5.3722	16.3106	4.5602	C	-3.1252	12.1699	4.2075
C	3.5230	15.4100	5.2174	C	0.9613	9.5656	4.9822	H	-4.1936	12.3330	4.3061
H	3.5560	15.8825	6.1963	H	1.5419	8.7303	5.3628				

Table S40. Atomic coordinates for optimised structure of **Bf-E** in its S_2 singlet excited state.

	x	*y*	*z*		*x*	*y*	*z*		*x*	*y*	*z*
O	-0.0425	13.5780	2.8298	C	3.2389	13.4575	1.4426	C	4.0377	13.4436	0.2926
N	0.9448	11.6428	3.7769	C	2.4574	14.5604	4.8818	H	4.9560	14.0243	0.2407
B	1.3569	12.9074	2.9673	H	1.6662	14.3827	5.6087	C	-0.4394	9.6056	5.2128
C	3.4658	14.1930	2.7048	C	2.4487	11.9297	-0.7569	H	-0.9452	8.8199	5.7614
C	-0.9636	12.8450	3.4233	H	2.1513	11.3319	-1.6153	C	-2.5594	11.0133	4.7780
C	2.4236	13.9363	3.6340	C	-2.3499	13.1092	3.5206	H	-3.2051	10.3137	5.3017
C	2.0409	12.6986	1.5080	H	-2.7805	14.0015	3.0793	C	1.6505	11.9490	0.3975
C	-0.4130	11.6801	4.0013	C	4.5297	15.0422	3.0334	H	0.7328	11.3633	0.4225
C	1.6342	10.5532	4.2857	H	5.3289	15.2407	2.3227	C	3.6355	12.6751	-0.8058
H	2.7025	10.5250	4.1088	C	4.5519	15.6482	4.2947	H	4.2471	12.6579	-1.7046
C	-1.1534	10.7307	4.6878	H	5.3722	16.3106	4.5602	C	-3.1252	12.1699	4.2075
C	3.5230	15.4100	5.2174	C	0.9613	9.5656	4.9822	H	-4.1936	12.3330	4.3061
H	3.5560	15.8825	6.1963	H	1.5419	8.7303	5.3628				

Table S41. Atomic coordinates for optimised structure of **Bf-E** in its T_1 triplet excited state.

	x	*y*	*z*		*x*	*y*	*z*		*x*	*y*	*z*
O	-0.1007	13.6269	2.8128	C	3.2362	13.4355	1.4532	C	4.0547	13.3860	0.3201
N	0.9170	11.6973	3.7520	C	2.4315	14.5856	4.8742	H	4.9968	13.9291	0.2853
B	1.3022	12.9816	2.9354	H	1.6206	14.4394	5.5865	C	-0.3887	9.6348	5.1856
C	3.4646	14.1736	2.7198	C	2.4389	11.9206	-0.7541	H	-0.8644	8.8319	5.7376
C	-0.9996	12.8669	3.4168	H	2.1326	11.3328	-1.6163	C	-2.5314	10.9777	4.7911
C	2.3949	13.9656	3.6250	C	-2.4124	13.1003	3.5356	H	-3.1626	10.2675	5.3175
C	2.0110	12.7258	1.4965	H	-2.8675	13.9848	3.1033	C	1.6226	11.9722	0.3885
C	-0.4197	11.7178	3.9806	C	4.5491	14.9866	3.0648	H	0.6851	11.4178	0.3996
C	1.6571	10.6170	4.2470	H	5.3722	15.1470	2.3716	C	3.6482	12.6245	-0.7843
H	2.7213	10.6323	4.0470	C	4.5687	15.6024	4.3239	H	4.2779	12.5813	-1.6699
C	-1.1402	10.7287	4.6852	H	5.4076	16.2369	4.6001	C	-3.1410	12.1518	4.2183
C	3.5170	15.4057	5.2262	C	1.0296	9.6175	4.9406	H	-4.2125	12.2792	4.3373
H	3.5418	15.8881	6.2006	H	1.6263	8.7912	5.3135				

Table S42. Atomic coordinates for optimised structure of **BPh_2-A1** in its ground state.

	x	*y*	*z*		*x*	*y*	*z*		*x*	*y*	*z*
N	5.6458	6.7997	7.1214	H	2.5397	4.8316	11.8452	H	8.2009	10.7144	7.9006
N	3.4355	5.7982	6.3958	C	6.7682	8.4583	5.9838	H	9.7088	10.2749	8.7285
C	3.4010	6.7351	5.3491	C	7.5189	8.0882	7.1006	H	8.1501	9.6915	9.3453

C	4.4673	7.6272	5.1488	C	2.3131	5.0492	6.3146	C	7.1795	9.5094	4.9909
C	5.5815	7.6490	6.0038	C	1.9747	3.9275	7.2447	H	7.1098	9.1564	3.9579
C	5.3059	4.2181	7.3407	H	2.8659	3.3898	7.5732	H	8.2144	9.8138	5.1718
C	3.6746	7.4481	9.2390	H	1.3073	3.2159	6.7506	H	6.5564	10.4083	5.0619
H	3.9907	8.2031	8.5214	H	1.4618	4.2948	8.1427	C	1.6671	7.3047	3.4231
C	3.9046	6.0851	8.9502	C	4.7952	9.0620	1.6436	H	1.6950	8.3878	3.5733
C	3.4680	5.1684	9.9261	H	5.1307	8.7362	0.6624	H	0.6313	7.0221	3.2142
H	3.5969	4.1026	9.7624	C	3.9411	9.8860	4.1726	H	2.2525	7.0943	2.5207
C	6.7942	7.0730	7.7803	H	3.6101	10.2076	5.1570	C	6.1500	2.4774	5.8134
C	5.8397	3.4555	8.3975	C	3.0600	7.8738	10.4206	H	6.2661	2.1093	4.7959
H	5.7511	3.8118	9.4194	H	2.9045	8.9362	10.5983	C	3.8897	10.7733	3.0941
C	4.8404	8.1731	2.7212	C	2.6488	6.9354	11.3741	H	3.5170	11.7832	3.2450
H	5.2093	7.1609	2.5753	H	2.1746	7.2580	12.2982	C	6.6590	1.7401	6.8887
C	7.2322	6.3903	9.0371	C	6.5003	2.2379	8.1845	H	7.1707	0.7957	6.7182
H	6.3848	6.1318	9.6747	H	6.8920	1.6819	9.0340	C	0.1835	4.8884	4.8341
H	7.9034	7.0403	9.6053	C	8.8440	8.6531	7.5452	H	-0.4815	5.6759	4.4586
H	7.7755	5.4622	8.8186	H	9.4524	8.9005	6.6666	H	-0.3163	4.4789	5.7204
C	4.4153	8.5787	3.9942	H	9.4089	7.8854	8.0878	C	0.2990	3.7834	3.7654
C	2.1792	6.5483	4.6171	C	1.5073	5.4903	5.2312	H	0.7514	4.1728	2.8457
C	5.4884	3.6873	6.0449	C	4.3182	10.3642	1.8268	H	-0.6899	3.3805	3.5144
H	5.0968	4.2327	5.1881	H	4.2807	11.0549	0.9885	H	0.9234	2.9542	4.1192
C	2.8558	5.5775	11.1187	C	8.7184	9.9074	8.4326	B	4.5752	5.6840	7.5017

Table S43. Atomic coordinates for optimised structure of **BPh_2-A1** in its S_1 singlet excited state.

	x	*y*	*z*		*x*	*y*	*z*		*x*	*y*	*z*
N	5.6632	6.7668	7.0717	H	2.6925	4.9298	11.9301	H	8.1146	10.6565	8.1251
N	3.4102	5.8602	6.4255	C	6.7459	8.5177	6.0147	H	9.6718	10.2109	8.8540
C	3.3966	6.7487	5.3301	C	7.5207	8.0939	7.0978	H	8.1641	9.5049	9.4706
C	4.4641	7.6637	5.1080	C	2.2768	5.1107	6.3643	C	7.0998	9.6545	5.0986
C	5.5801	7.6699	5.9914	C	1.9155	4.0524	7.3462	H	7.3922	9.3131	4.0973
C	5.2375	4.1974	7.3092	H	2.7710	3.4125	7.5870	H	7.9403	10.2215	5.5099
C	3.7909	7.4910	9.2543	H	1.1111	3.4253	6.9539	H	6.2668	10.3514	4.9608
H	4.0941	8.2312	8.5158	H	1.5705	4.4840	8.2974	C	1.7478	7.1767	3.2956
C	3.9706	6.1199	8.9680	C	5.1253	9.2463	1.7132	H	1.4200	8.2119	3.4560
C	3.5502	5.2245	9.9713	H	5.7138	9.0386	0.8230	H	0.9053	6.6295	2.8622
H	3.6398	4.1542	9.8124	C	3.6107	9.7590	4.0023	H	2.5412	7.2130	2.5420
C	6.8228	7.0209	7.7351	H	3.0223	9.9623	4.8937	C	5.9625	2.4331	5.7470
C	5.7635	3.4007	8.3454	C	3.2404	7.9441	10.4571	H	6.0361	2.0698	4.7238
H	5.7201	3.7513	9.3720	H	3.1226	9.0119	10.6310	C	3.5666	10.6453	2.9229
C	5.1726	8.3648	2.7965	C	2.8434	7.0258	11.4362	H	2.9429	11.5340	2.9800
H	5.7962	7.4757	2.7437	H	2.4177	7.3697	12.3760	C	6.4644	1.6631	6.8027
C	7.2825	6.2785	8.9405	C	6.3631	2.1564	8.1058	H	6.9281	0.6982	6.6113
H	6.4615	6.0908	9.6394	H	6.7523	1.5765	8.9403	C	0.1915	4.8795	4.8219
H	8.0616	6.8409	9.4616	C	8.8329	8.6657	7.5576	H	-0.4372	5.6374	4.3392
H	7.7010	5.2960	8.6753	H	9.4028	9.0186	6.6895	H	-0.3598	4.5566	5.7134

C	4.4152	8.6075	3.9553	H	9.4421	7.8770	8.0156	C	0.3429	3.6755	3.8661
C	2.1979	6.5117	4.5652	C	1.5006	5.4976	5.2274	H	0.8550	3.9688	2.9425
C	5.3640	3.6701	6.0054	C	4.3229	10.3912	1.7731	H	-0.6419	3.2731	3.5984
H	4.9797	4.2416	5.1623	H	4.2873	11.0788	0.9321	H	0.9244	2.8712	4.3314
C	3.0001	5.6606	11.1848	C	8.6855	9.8294	8.5627	B	4.5721	5.6954	7.4925

Table S44. Atomic coordinates for optimised structure of **BPh$_2$-A1** in its S_2 singlet excited state.

	x	*y*	*z*		*x*	*y*	*z*		*x*	*y*	*z*
N	-0.4183	1.2119	0.2624	H	-5.2740	-0.2018	-2.8419	H	1.0277	5.4597	-1.0830
N	-0.3670	-1.2289	-0.2991	C	1.3908	2.5615	0.3875	H	0.1468	6.7449	-0.2337
C	1.0328	-1.2029	-0.2656	C	0.1903	3.3599	0.6065	H	-0.7433	5.4474	-1.0576
C	1.7219	0.0200	-0.0205	C	-0.7746	-2.5397	-0.5592	C	2.7657	3.1453	0.3424
C	0.9764	1.2290	0.2228	C	-2.2044	-2.9585	-0.7013	H	3.3346	2.9230	1.2566
C	-2.1158	-0.3256	1.4372	H	-2.8359	-2.5474	0.0915	H	2.7253	4.2334	0.2420
C	-1.5755	0.8679	-2.4717	H	-2.2731	-4.0486	-0.6469	H	3.3559	2.7452	-0.4881
H	-0.5345	1.1711	-2.3885	H	-2.6373	-2.6454	-1.6592	C	2.9104	-3.0518	-0.4336
C	-2.2162	0.2625	-1.3722	C	5.3268	0.1972	1.1906	H	3.4620	-2.8031	-1.3509
C	-3.5546	-0.1199	-1.5427	H	5.8615	0.2725	2.1344	H	2.9116	-4.1419	-0.3425
H	-4.0895	-0.6220	-0.7444	C	3.9375	-0.0094	-1.2204	H	3.4968	-2.6457	0.3969
C	-0.8903	2.5168	0.5292	H	3.3988	-0.0842	-2.1621	C	-1.9627	-1.1624	3.7355
C	-3.4596	-0.0102	1.6889	C	-2.2449	1.0958	-3.6782	H	-1.3632	-1.6079	4.5266
H	-4.0683	0.4600	0.9255	H	-1.7183	1.5702	-4.5039	C	5.3343	0.0308	-1.2212
C	3.9299	0.1634	1.1861	C	-3.5855	0.7223	-3.8204	H	5.8747	-0.0160	-2.1636
H	3.3861	0.2094	2.1268	H	-4.1122	0.9024	-4.7544	C	-3.3131	-0.8694	3.9551
C	-2.3328	2.8807	0.6906	C	-4.0584	-0.2913	2.9256	H	-3.7739	-1.0822	4.9167
H	-2.9629	2.4282	-0.0794	H	-5.1078	-0.0486	3.0785	C	0.3661	-4.8364	-0.9008
H	-2.4424	3.9657	0.6102	C	0.1684	4.8439	0.8401	H	1.2498	-5.0985	-1.4959
H	-2.7328	2.5776	1.6653	H	1.0455	5.1345	1.4318	H	-0.4987	-5.1247	-1.5105
C	3.2096	0.0567	-0.0182	H	-0.7047	5.1109	1.4463	C	0.3689	-5.6802	0.3916
C	1.5057	-2.5243	-0.4490	C	0.3465	-3.3511	-0.6547	H	1.2416	-5.4482	1.0131
C	-1.3759	-0.8876	2.4966	C	6.0357	0.1327	-0.0144	H	0.3948	-6.7511	0.1550
H	-0.3274	-1.1334	2.3490	H	7.1224	0.1617	-0.0130	H	-0.5287	-5.4845	0.9899
C	-4.2356	0.1088	-2.7478	C	0.1494	5.6727	-0.4629	B	-1.2826	-0.0295	-0.0019

Table S45. Atomic coordinates for optimised structure of **BPh$_2$-A1** in its T_1 triplet excited state.

	x	*y*	*z*		*x*	*y*	*z*		*x*	*y*	*z*
N	5.6505	6.7891	7.0775	H	2.6538	4.9103	11.9066	H	8.1376	10.6846	8.1184
N	3.4269	5.8466	6.4017	C	6.7648	8.5072	6.0207	H	9.6906	10.2342	8.8519
C	3.4104	6.7273	5.3482	C	7.5280	8.1139	7.1114	H	8.1788	9.5393	9.4694
C	4.4658	7.6523	5.1180	C	2.2694	5.0709	6.3307	C	7.1153	9.6078	5.0642
C	5.5715	7.6524	6.0125	C	1.9176	4.0089	7.3134	H	7.2698	9.2369	4.0440
C	5.2579	4.2066	7.3189	H	2.7826	3.3922	7.5723	H	8.0360	10.1056	5.3814
C	3.7576	7.4804	9.2439	H	1.1395	3.3585	6.9053	H	6.3295	10.3683	5.0045
H	4.0630	8.2235	8.5094	H	1.5376	4.4382	8.2500	C	1.7314	7.2136	3.3358

C	3.9512	6.1113	8.9579	C	4.9789	9.1656	1.6567	H	1.5390	8.2781	3.5149
C	3.5274	5.2131	9.9569	H	5.4575	8.8963	0.7184	H	0.8076	6.7666	2.9578
H	3.6251	4.1433	9.7980	C	3.7555	9.8369	4.0719	H	2.4779	7.1613	2.5361
C	6.8355	7.0602	7.7625	H	3.2783	10.1028	5.0124	C	6.0099	2.4422	5.7681
C	5.7850	3.4183	8.3606	C	3.1917	7.9293	10.4415	H	6.0929	2.0760	4.7467
H	5.7323	3.7727	9.3857	H	3.0641	8.9962	10.6140	C	3.7060	10.7258	2.9940
C	5.0247	8.2813	2.7386	C	2.7926	7.0080	11.4158	H	3.1906	11.6770	3.1017
H	5.5387	7.3285	2.6348	H	2.3554	7.3475	12.3518	C	6.5127	1.6818	6.8294
C	7.2856	6.3193	8.9732	C	6.3979	2.1797	8.1300	H	6.9870	0.7207	6.6451
H	6.4625	6.1445	9.6714	H	6.7870	1.6069	8.9694	C	0.1818	4.8637	4.8032
H	8.0656	6.8818	9.4929	C	8.8453	8.6895	7.5621	H	-0.4464	5.6276	4.3304
H	7.6996	5.3363	8.7122	H	9.4113	9.0351	6.6894	H	-0.3661	4.5384	5.6958
C	4.4147	8.6015	3.9626	H	9.4549	7.9006	8.0191	C	0.3269	3.6686	3.8398
C	2.1820	6.5024	4.5768	C	1.4967	5.4752	5.2111	H	0.8303	3.9695	2.9136
C	5.3975	3.6743	6.0184	C	4.3183	10.3924	1.7813	H	-0.6585	3.2652	3.5771
H	5.0114	4.2377	5.1709	H	4.2813	11.0819	0.9417	H	0.9142	2.8618	4.2937
C	2.9626	5.6439	11.1646	C	8.7032	9.8561	8.5602	B	4.5740	5.6953	7.4909

Table S46. Atomic coordinates for optimised structure of **BPh_2-A2** in its ground state.

	x	*y*	*z*		*x*	*y*	*z*		*x*	*y*	*z*
N	0.8098	1.7664	2.6499	H	2.1453	0.2962	-1.0374	C	-0.0624	5.4610	3.5114
N	-1.2932	3.0492	2.1610	H	3.5421	-0.2905	-0.1337	C	0.5928	6.6641	3.8015
C	1.7076	0.9481	3.2511	H	3.4849	1.3848	-0.6704	C	1.9343	6.6567	4.1958
C	2.5830	0.3937	2.2799	C	0.7147	2.6538	-1.0625	C	2.6044	5.4327	4.2847
C	2.2242	0.9293	1.0393	H	0.3328	3.5816	-1.4866	C	1.9330	4.2396	3.9908
C	1.1056	1.7924	1.2766	H	0.3527	1.8281	-1.6891	C	-0.9310	2.1123	4.5971
C	0.2844	2.4978	0.3765	H	1.8005	2.6793	-1.1458	C	-0.8974	2.6730	5.8852
C	-0.9409	3.0459	0.8011	C	-2.1950	3.6721	-1.4461	C	-1.5240	2.0614	6.9786
C	-2.0489	3.5519	0.0466	H	-3.2360	3.8733	-1.7124	C	-2.2108	0.8551	6.8085
C	-3.0523	3.8587	0.9705	H	-1.8979	2.7562	-1.9672	C	-2.2614	0.2724	5.5362
C	-2.5484	3.5506	2.2628	H	-1.5924	4.4908	-1.8609	C	-1.6299	0.8972	4.4564
C	1.7717	0.6658	4.7227	C	-4.4356	4.3801	0.6743	B	-0.2137	2.7940	3.2986
H	2.7477	0.2454	4.9782	H	-4.7304	5.1086	1.4405	H	2.4959	3.3120	4.0552
H	1.0041	-0.0565	5.0261	H	-4.4236	4.9377	-0.2692	H	3.6522	5.4046	4.5774
H	1.6195	1.5617	5.3269	C	-5.5117	3.2796	0.5937	H	2.4499	7.5874	4.4209
C	3.6924	-0.5880	2.5616	H	-6.4960	3.7131	0.3778	H	0.0554	7.6063	3.7135
H	3.4373	-1.1957	3.4377	H	-5.5844	2.7274	1.5382	H	-1.1000	5.5130	3.1916
H	3.7772	-1.2967	1.7284	H	-5.2764	2.5579	-0.1978	H	-0.3693	3.6112	6.0432
C	5.0652	0.0749	2.7903	C	-3.2955	3.7551	3.5463	H	-1.4759	2.5264	7.9613
H	5.8342	-0.6819	2.9885	H	-4.1540	4.4097	3.3750	H	-2.6993	0.3753	7.6535
H	5.0357	0.7609	3.6453	H	-2.6752	4.2029	4.3253	H	-2.7917	-0.6663	5.3877
H	5.3742	0.6516	1.9105	H	-3.6724	2.8064	3.9460	H	-1.6825	0.4252	3.4759
C	2.8750	0.5658	-0.2670	C	0.5774	4.2081	3.6042				

Table S47. Atomic coordinates for optimised structure of **BPh_2-A2** in its S_1 singlet excited state.

	x	*y*	*z*		*x*	*y*	*z*		*x*	*y*	*z*
N	0.8072	1.7902	2.6666	H	1.9802	0.4382	-1.0794	C	-0.0525	5.4941	3.5191
N	-1.2826	3.0653	2.1774	H	3.2620	-0.4431	-0.2451	C	0.6045	6.6985	3.7992
C	1.6744	0.9206	3.2572	H	3.4917	1.2509	-0.6697	C	1.9482	6.6923	4.1866
C	2.5268	0.3425	2.2632	C	0.9153	3.0192	-0.9581	C	2.6188	5.4688	4.2810
C	2.1731	0.8982	1.0279	H	0.5322	3.9936	-1.2745	C	1.9459	4.2741	3.9972
C	1.1103	1.8236	1.2911	H	0.6599	2.3006	-1.7504	C	-0.9139	2.1665	4.6283
C	0.3727	2.6530	0.3971	H	2.0066	3.0884	-0.9287	C	-0.8643	2.7421	5.9094
C	-0.9226	3.0723	0.8149	C	-2.1642	3.5559	-1.4578	C	-1.4849	2.1478	7.0162
C	-2.0506	3.4998	0.0400	H	-3.2150	3.5663	-1.7633	C	-2.1813	0.9446	6.8671
C	-3.0699	3.7895	0.9537	H	-1.6951	2.6890	-1.9354	C	-2.2485	0.3473	5.6020
C	-2.5649	3.5178	2.2657	H	-1.6957	4.4522	-1.8879	C	-1.6232	0.9549	4.5089
C	1.7310	0.6189	4.7180	C	-4.4608	4.2629	0.6356	B	-0.2014	2.8310	3.3178
H	2.6751	0.1246	4.9614	H	-4.7897	4.9955	1.3840	H	2.5054	3.3440	4.0679
H	0.9130	-0.0470	5.0286	H	-4.4524	4.7968	-0.3221	H	3.6673	5.4428	4.5716
H	1.6437	1.5184	5.3340	C	-5.5032	3.1258	0.5650	H	2.4649	7.6241	4.4046
C	3.6090	-0.6687	2.5196	H	-6.4938	3.5288	0.3207	H	0.0671	7.6408	3.7104
H	3.3639	-1.2654	3.4061	H	-5.5781	2.5966	1.5219	H	-1.0934	5.5408	3.2069
H	3.6496	-1.3780	1.6828	H	-5.2329	2.3928	-0.2042	H	-0.3284	3.6782	6.0512
C	5.0105	-0.0470	2.7036	C	-3.3264	3.7085	3.5343	H	-1.4241	2.6240	7.9928
H	5.7596	-0.8311	2.8687	H	-4.2108	4.3242	3.3492	H	-2.6651	0.4781	7.7222
H	5.0321	0.6317	3.5642	H	-2.7278	4.1868	4.3153	H	-2.7868	-0.5891	5.4698
H	5.3048	0.5252	1.8163	H	-3.6675	2.7505	3.9517	H	-1.6887	0.4723	3.5345
C	2.7518	0.5237	-0.3070	C	0.5892	4.2420	3.6142				

Table S48. Atomic coordinates for optimised structure of **BPh$_2$-A2** in its S_2 singlet excited state.

	x	*y*	*z*		*x*	*y*	*z*		*x*	*y*	*z*
N	0.8072	1.7902	2.6666	H	1.9802	0.4382	-1.0794	C	-0.0525	5.4941	3.5191
N	-1.2826	3.0653	2.1774	H	3.2620	-0.4431	-0.2451	C	0.6045	6.6985	3.7992
C	1.6744	0.9206	3.2572	H	3.4917	1.2509	-0.6697	C	1.9482	6.6923	4.1866
C	2.5268	0.3425	2.2632	C	0.9153	3.0192	-0.9581	C	2.6188	5.4688	4.2810
C	2.1731	0.8982	1.0279	H	0.5322	3.9936	-1.2745	C	1.9459	4.2741	3.9972
C	1.1103	1.8236	1.2911	H	0.6599	2.3006	-1.7504	C	-0.9139	2.1665	4.6283
C	0.3727	2.6530	0.3971	H	2.0066	3.0884	-0.9287	C	-0.8643	2.7421	5.9094
C	-0.9226	3.0723	0.8149	C	-2.1642	3.5559	-1.4578	C	-1.4849	2.1478	7.0162
C	-2.0506	3.4998	0.0400	H	-3.2150	3.5663	-1.7633	C	-2.1813	0.9446	6.8671
C	-3.0699	3.7895	0.9537	H	-1.6951	2.6890	-1.9354	C	-2.2485	0.3473	5.6020
C	-2.5649	3.5178	2.2657	H	-1.6957	4.4522	-1.8879	C	-1.6232	0.9549	4.5089
C	1.7310	0.6189	4.7180	C	-4.4608	4.2629	0.6356	B	-0.2014	2.8310	3.3178
H	2.6751	0.1246	4.9614	H	-4.7897	4.9955	1.3840	H	2.5054	3.3440	4.0679
H	0.9130	-0.0470	5.0286	H	-4.4524	4.7968	-0.3221	H	3.6673	5.4428	4.5716
H	1.6437	1.5184	5.3340	C	-5.5032	3.1258	0.5650	H	2.4649	7.6241	4.4046
C	3.6090	-0.6687	2.5196	H	-6.4938	3.5288	0.3207	H	0.0671	7.6408	3.7104
H	3.3639	-1.2654	3.4061	H	-5.5781	2.5966	1.5219	H	-1.0934	5.5408	3.2069

H	3.6496	-1.3780	1.6828	H	-5.2329	2.3928	-0.2042	H	-0.3284	3.6782	6.0512
C	5.0105	-0.0470	2.7036	C	-3.3264	3.7085	3.5343	H	-1.4241	2.6240	7.9928
H	5.7596	-0.8311	2.8687	H	-4.2108	4.3242	3.3492	H	-2.6651	0.4781	7.7222
H	5.0321	0.6317	3.5642	H	-2.7278	4.1868	4.3153	H	-2.7868	-0.5891	5.4698
H	5.3048	0.5252	1.8163	H	-3.6675	2.7505	3.9517	H	-1.6887	0.4723	3.5345
C	2.7518	0.5237	-0.3070	C	0.5892	4.2420	3.6142				

Table S49. Atomic coordinates for optimised structure of **BPh$_2$-A2** in its T_1 triplet excited state.

	x	*y*	*z*		*x*	*y*	*z*		*x*	*y*	*z*
N	5.6111	6.8727	7.2178	C	6.9393	8.3272	6.0193	H	8.0803	10.0044	5.3194
N	3.4281	5.9726	6.4186	C	7.4166	8.2894	7.3232	H	6.6129	9.7035	4.3855
C	3.6891	6.5353	5.1881	C	2.1061	5.5380	6.4148	C	2.2582	7.0961	3.0287
C	4.9615	7.0824	4.8678	C	1.4365	4.8368	7.5494	H	2.7170	8.0868	2.9432
C	5.8099	7.4047	5.9620	H	2.0712	4.0638	7.9908	H	1.1896	7.2063	2.8200
C	5.3251	4.2537	7.0618	H	0.5120	4.3670	7.2041	H	2.6789	6.4762	2.2258
C	3.3635	6.8993	9.4412	H	1.1810	5.5308	8.3608	C	5.2175	1.9486	6.1975
H	3.2588	7.7497	8.7685	C	2.8478	7.0014	10.7366	H	4.6234	1.1428	5.7706
C	4.0116	5.7359	8.9804	H	2.3537	7.9171	11.0554	C	6.5846	1.7646	6.4274
C	4.1162	4.6723	9.8932	C	2.9673	5.9242	11.6237	H	7.0635	0.8185	6.1859
H	4.6083	3.7526	9.5835	H	2.5687	5.9958	12.6332	C	0.0732	5.5865	4.7911
C	6.5966	7.3841	8.0564	C	7.3275	2.8207	6.9647	H	-0.3007	6.3899	4.1448
C	6.7026	4.0360	7.2703	H	8.3947	2.7017	7.1418	H	-0.5599	5.6048	5.6861
H	7.3178	4.8377	7.6725	C	8.5531	9.0920	7.9004	C	-0.1141	4.2384	4.0665
C	6.7583	7.0295	9.4964	H	9.2501	9.3633	7.0994	H	0.4708	4.2069	3.1398
H	5.9974	7.5150	10.1210	H	9.1293	8.4719	8.5989	H	-1.1687	4.0814	3.8096
H	7.7404	7.3538	9.8510	C	1.5034	5.8713	5.1686	H	0.2104	3.4015	4.6961
H	6.6649	5.9543	9.6726	C	8.0978	10.3730	8.6277	B	4.5948	5.6741	7.4560
C	2.4679	6.4988	4.3895	H	7.5580	11.0405	7.9457	C	5.3671	7.3664	3.4390
C	4.6101	3.1710	6.5096	H	8.9619	10.9183	9.0261	H	5.0998	8.3695	3.0780
H	3.5479	3.2794	6.3020	H	7.4300	10.1382	9.4648	H	6.4486	7.2589	3.3188
C	3.6054	4.7562	11.1951	C	7.4378	9.2162	4.9165	H	4.9056	6.6456	2.7587
H	3.7058	3.9108	11.8730	H	8.0251	8.6714	4.1656				

Table S50. Atomic coordinates for optimised structure of **BF$_2$-A1** in its ground state.

	x	*y*	*z*		*x*	*y*	*z*		*x*	*y*	*z*
F	0.3011	4.9937	9.4055	C	-2.8043	1.6340	9.3388	H	-5.7022	2.9630	12.4026
F	-0.6813	5.1630	7.3366	C	-3.3789	0.2551	9.4947	C	-0.6102	-0.2316	8.1620
N	0.7879	3.2783	7.7490	H	-2.8219	-0.3368	10.2301	C	-2.7897	5.3476	9.6895
N	-1.4494	3.4120	8.8430	H	-3.3625	-0.3079	8.5567	H	-2.1984	5.6805	10.5522
C	4.0105	2.4235	6.1119	H	-4.4162	0.3110	9.8357	H	-3.8411	5.5475	9.9094
H	4.5533	1.5166	6.4038	C	-1.5432	2.0148	8.7724	H	-2.4890	5.9586	8.8341
H	4.6205	3.2617	6.4702	C	-1.2133	-2.2555	6.9624	C	-2.5734	3.8910	9.4267
C	1.8513	1.3542	7.1082	H	-1.5990	-2.7336	6.0657	C	-1.1031	-0.8631	7.0113
C	3.9292	2.4859	4.5739	C	0.6623	1.8822	7.7118	H	-1.4020	-0.2638	6.1550

H	4.9328	2.4649	4.1320	C	2.4191	5.0399	7.0429	C	-0.8274	-3.0298	8.0615
H	3.4312	3.4046	4.2419	H	1.9309	5.5103	6.1794	H	-0.9115	-4.1127	8.0226
H	3.3638	1.6354	4.1751	H	3.4987	5.0908	6.8814	C	-0.4942	1.2592	8.2164
C	-3.4402	2.8158	9.7440	H	2.1685	5.6362	7.9240	C	-0.3328	-2.4047	9.2110
C	2.6652	2.4499	6.7897	C	-0.2269	-1.0120	9.2621	H	-0.0303	-2.9992	10.0691
C	1.9755	3.6195	7.1957	H	0.1560	-0.5284	10.1572	B	-0.2577	4.2500	8.3351
C	2.2040	-0.0812	6.8424	C	-4.7037	2.8626	11.9599	C	-4.7792	2.9464	10.4227
H	1.5666	-0.5194	6.0658	H	-4.0697	3.6587	12.3678	H	-5.4569	2.1665	10.0555
H	2.0930	-0.7059	7.7336	H	-4.2839	1.9021	12.2813	H	-5.2443	3.8988	10.1406
H	3.2397	-0.1619	6.5014								

Table S51. Atomic coordinates for optimised structure of **BF_2-A1** in its S_1 singlet excited state.

	x	*y*	*z*		*x*	*y*	*z*		*x*	*y*	*z*
F	0.3581	5.0450	9.3550	C	-2.8122	1.6695	9.3350	H	-5.6883	2.7806	12.4305
F	-0.7507	5.2026	7.3552	C	-3.4386	0.3073	9.4102	C	-0.6123	-0.2297	8.1589
N	0.7468	3.3191	7.6879	H	-3.0263	-0.2990	10.2276	C	-2.7106	5.3853	9.7762
N	-1.4094	3.4424	8.8976	H	-3.2950	-0.2644	8.4889	H	-1.9248	5.7694	10.4410
C	3.9864	2.4715	6.0780	H	-4.5155	0.3966	9.5842	H	-3.6784	5.5707	10.2462
H	4.5856	1.6482	6.4873	C	-1.5380	2.0364	8.7901	H	-2.6517	5.9766	8.8529
H	4.5373	3.3904	6.3080	C	-0.9960	-2.2759	6.8940	C	-2.5210	3.9335	9.5022
C	1.8631	1.3876	7.1166	H	-1.2135	-2.7646	5.9476	C	-0.8843	-0.8841	6.9450
C	3.9042	2.3077	4.5437	C	0.6561	1.9061	7.6910	H	-1.0168	-0.2967	6.0398
H	4.9110	2.2851	4.1098	C	2.3423	5.0963	6.9543	C	-0.8295	-3.0378	8.0559
H	3.3539	3.1388	4.0881	H	1.6126	5.6564	6.3543	H	-0.9131	-4.1207	8.0161
H	3.3943	1.3760	4.2739	H	3.3156	5.1638	6.4644	C	-0.4971	1.2540	8.2134
C	-3.4133	2.8563	9.7908	H	2.4043	5.6083	7.9241	C	-0.5547	-2.3971	9.2693
C	2.6447	2.4975	6.7497	C	-0.4511	-1.0048	9.3204	H	-0.4201	-2.9811	10.1763
C	1.9263	3.6755	7.1185	H	-0.2351	-0.5122	10.2652	B	-0.2615	4.2834	8.3251
C	2.2710	-0.0458	6.9366	C	-4.6899	2.6817	11.9881	C	-4.7434	2.9848	10.4739
H	1.7737	-0.5181	6.0791	H	-4.0164	3.3749	12.5048	H	-5.4638	2.3025	10.0048
H	2.0357	-0.6553	7.8138	H	-4.3332	1.6622	12.1734	H	-5.1434	3.9944	10.3282
H	3.3496	-0.1121	6.7625								

Table S52. Atomic coordinates for optimised structure of **BF_2-A1** in its T_1 triplet excited state.

	x	*y*	*z*		*x*	*y*	*z*		*x*	*y*	*z*
F	0.2929	5.0014	9.3986	C	-2.8262	1.6452	9.3563	H	-5.7307	2.9995	12.3905
F	-0.6888	5.1598	7.3324	C	-3.3686	0.2580	9.5179	C	-0.6114	-0.2538	8.1562
N	0.7769	3.2696	7.7625	H	-2.7254	-0.3615	10.1534	C	-2.8198	5.3612	9.6328
N	-1.4450	3.4002	8.8296	H	-3.4566	-0.2643	8.5585	H	-2.0431	5.7832	10.2827
C	4.0404	2.4306	6.1319	H	-4.3604	0.2875	9.9770	H	-3.7922	5.5473	10.0937
H	4.5839	1.5306	6.4424	C	-1.5353	2.0316	8.7716	H	-2.7727	5.9120	8.6853
H	4.6415	3.2778	6.4820	C	-1.2839	-2.2768	6.9726	C	-2.6083	3.9026	9.4200
C	1.8778	1.3686	7.1007	H	-1.7224	-2.7456	6.0952	C	-1.1737	-0.8844	7.0345

C	3.9649	2.4699	4.5929	C	0.6575	1.9027	7.7192	H	-1.5271	-0.2768	6.2047
H	4.9711	2.4464	4.1578	C	2.4392	5.0525	7.1007	C	-0.8312	-3.0637	8.0367
H	3.4655	3.3820	4.2459	H	1.7385	5.6347	6.4895	H	-0.9159	-4.1464	7.9908
H	3.4050	1.6119	4.2036	H	3.4307	5.1230	6.6485	C	-0.4949	1.2376	8.2178
C	-3.4642	2.8215	9.7432	H	2.4717	5.5315	8.0870	C	-0.2689	-2.4491	9.1603
C	2.6901	2.4593	6.7985	C	-0.1611	-1.0564	9.2168	H	0.0858	-3.0525	9.9922
C	2.0073	3.6312	7.2058	H	0.2769	-0.5832	10.0925	B	-0.2636	4.2404	8.3318
C	2.2066	-0.0693	6.8377	C	-4.7302	2.9027	11.9526	C	-4.8065	2.9515	10.4140
H	1.4872	-0.5364	6.1552	H	-4.1078	3.7161	12.3433	H	-5.4711	2.1541	10.0620
H	2.2003	-0.6673	7.7559	H	-4.2982	1.9554	12.2953	H	-5.2810	3.8916	10.1087
H	3.1985	-0.1566	6.3862								

Table S53. Atomic coordinates for optimised structure of **BF_2-A2** in its ground state.

	x	*y*	*z*		*x*	*y*	*z*		*x*	*y*	*z*
F	0.4397	4.8385	9.6260	H	1.2900	-0.6125	6.2933	H	-4.4931	1.8512	12.1624
F	-0.5357	5.2379	7.5850	H	2.4234	-0.7000	7.6450	H	-5.8689	2.9698	12.2454
N	0.8296	3.2468	7.8285	H	2.9773	-0.1574	6.0631	C	-2.7618	5.3766	9.6625
N	-1.3988	3.4087	8.9256	C	-2.7960	1.6576	9.3804	H	-1.8586	5.8705	10.0306
C	3.9438	2.3791	6.0035	C	-3.4248	0.2966	9.4881	H	-3.5663	5.5454	10.3823
H	4.4681	1.4384	6.2086	H	-2.9898	-0.2986	10.3019	H	-3.0430	5.8691	8.7225
H	4.6051	3.1720	6.3748	H	-3.3203	-0.2847	8.5665	C	-2.5391	3.9108	9.4559
C	1.8324	1.3214	7.1124	H	-4.4938	0.3890	9.6989	C	-0.4486	1.2333	8.3525
C	3.7768	2.5425	4.4800	C	-1.5043	2.0119	8.8706	B	-0.1615	4.2208	8.4953
H	4.7515	2.5138	3.9778	C	0.6896	1.8523	7.7955	C	-4.8111	3.0126	10.3341
H	3.2973	3.4979	4.2358	C	2.4585	4.9978	7.0859	H	-5.4958	2.2852	9.8805
H	3.1555	1.7407	4.0635	H	1.9365	5.5049	6.2643	H	-5.2129	3.9985	10.0717
C	-3.4349	2.8536	9.7403	H	3.5294	5.0305	6.8700	C	-0.5342	-0.2717	8.4020
C	2.6422	2.4119	6.7626	H	2.2690	5.5735	7.9960	H	-1.0101	-0.6740	7.4974
C	1.9941	3.5821	7.2242	C	-4.8473	2.8455	11.8659	H	0.4532	-0.7222	8.4835
C	2.1402	-0.1086	6.7643	H	-4.2088	3.5885	12.3586	H	-1.1164	-0.6072	9.2584

Table S54. Atomic coordinates for optimised structure of **BF_2-A2** in its S_1 singlet excited state.

	x	*y*	*z*		*x*	*y*	*z*		*x*	*y*	*z*
F	0.4871	4.8268	9.6995	H	1.1645	-0.6192	6.3845	H	-4.5647	1.7457	12.0301
F	-0.4729	5.2832	7.6680	H	2.5049	-0.6974	7.5312	H	-5.9315	2.8735	12.1423
N	0.8501	3.2607	7.8679	H	2.7882	-0.1558	5.8788	C	-2.7252	5.4020	9.6716
N	-1.3648	3.4298	8.9549	C	-2.7981	1.6742	9.3510	H	-1.8896	5.8437	10.2287
C	3.9047	2.3733	5.9479	C	-3.4336	0.3135	9.3973	H	-3.6476	5.5860	10.2266
H	4.4206	1.4233	6.1318	H	-3.1384	-0.2637	10.2848	H	-2.7851	5.9447	8.7174
H	4.5848	3.1600	6.2972	H	-3.1821	-0.2900	8.5196	C	-2.5193	3.9421	9.4546
C	1.8171	1.3223	7.0922	H	-4.5239	0.4057	9.4293	C	-0.4008	1.2136	8.4496
C	3.6914	2.5419	4.4269	C	-1.4840	2.0216	8.8991	B	-0.1195	4.2322	8.5543
H	4.6526	2.4975	3.9007	C	0.7075	1.8538	7.8264	C	-4.8177	3.0290	10.2713
H	3.2214	3.5051	4.1983	C	2.4754	5.0043	7.1063	H	-5.5001	2.3394	9.7573
H	3.0466	1.7498	4.0294	H	1.8604	5.5693	6.3909	H	-5.1959	4.0369	10.0663

C	-3.4346	2.8764	9.7079	H	3.5105	5.0346	6.7576	C	-0.4200	-0.2796	8.6449
C	2.6293	2.4136	6.7396	H	2.4116	5.5377	8.0624	H	-0.8777	-0.8214	7.8043
C	2.0033	3.5972	7.2345	C	-4.8989	2.7615	11.7906	H	0.5930	-0.6684	8.7672
C	2.0766	-0.1078	6.7085	H	-4.2695	3.4644	12.3484	H	-0.9788	-0.5462	9.5445

Table S55. Atomic coordinates for optimised structure of **BF_2-A2** in its T_1 triplet excited state.

	x	*y*	*z*		*x*	*y*	*z*		*x*	*y*	*z*
F	0.4610	4.8216	9.6663	H	1.1805	-0.5923	6.3291	H	-4.5218	1.8618	12.1417
F	-0.5011	5.2477	7.6290	H	2.4484	-0.7125	7.5508	H	-5.9094	2.9671	12.2051
N	0.8337	3.2340	7.8552	H	2.8449	-0.1693	5.9210	C	-2.7528	5.3828	9.6733
N	-1.3761	3.3981	8.9363	C	-2.8191	1.6685	9.3680	H	-1.9239	5.8160	10.2456
C	3.9374	2.3633	5.9578	C	-3.4344	0.3011	9.4140	H	-3.6826	5.5749	10.2131
H	4.4373	1.4018	6.1215	H	-3.0756	-0.2936	10.2648	H	-2.7920	5.9202	8.7170
H	4.6251	3.1269	6.3420	H	-3.2249	-0.2739	8.5061	C	-2.5573	3.9207	9.4648
C	1.8416	1.3227	7.0862	H	-4.5211	0.3776	9.5149	C	-0.4189	1.2118	8.4130
C	3.7409	2.5814	4.4443	C	-1.4817	2.0280	8.8835	B	-0.1405	4.2084	8.5274
H	4.7043	2.5446	3.9219	C	0.6932	1.8664	7.8196	C	-4.8370	3.0087	10.3041
H	3.2817	3.5559	4.2412	C	2.4828	4.9933	7.1011	H	-5.5070	2.2689	9.8495
H	3.0912	1.8092	4.0162	H	1.8486	5.5601	6.4066	H	-5.2450	3.9889	10.0315
C	-3.4536	2.8561	9.7284	H	3.5109	5.0358	6.7337	C	-0.4535	-0.2926	8.5695
C	2.6515	2.4023	6.7417	H	2.4357	5.5099	8.0666	H	-0.9080	-0.8163	7.7169
C	2.0262	3.5805	7.2192	C	-4.8831	2.8508	11.8368	H	0.5536	-0.6934	8.6978
C	2.0873	-0.1087	6.7074	H	-4.2572	3.6037	12.3303	H	-1.0126	-0.5784	9.4623

8. NMR spectra

NMR spectra of new compounds

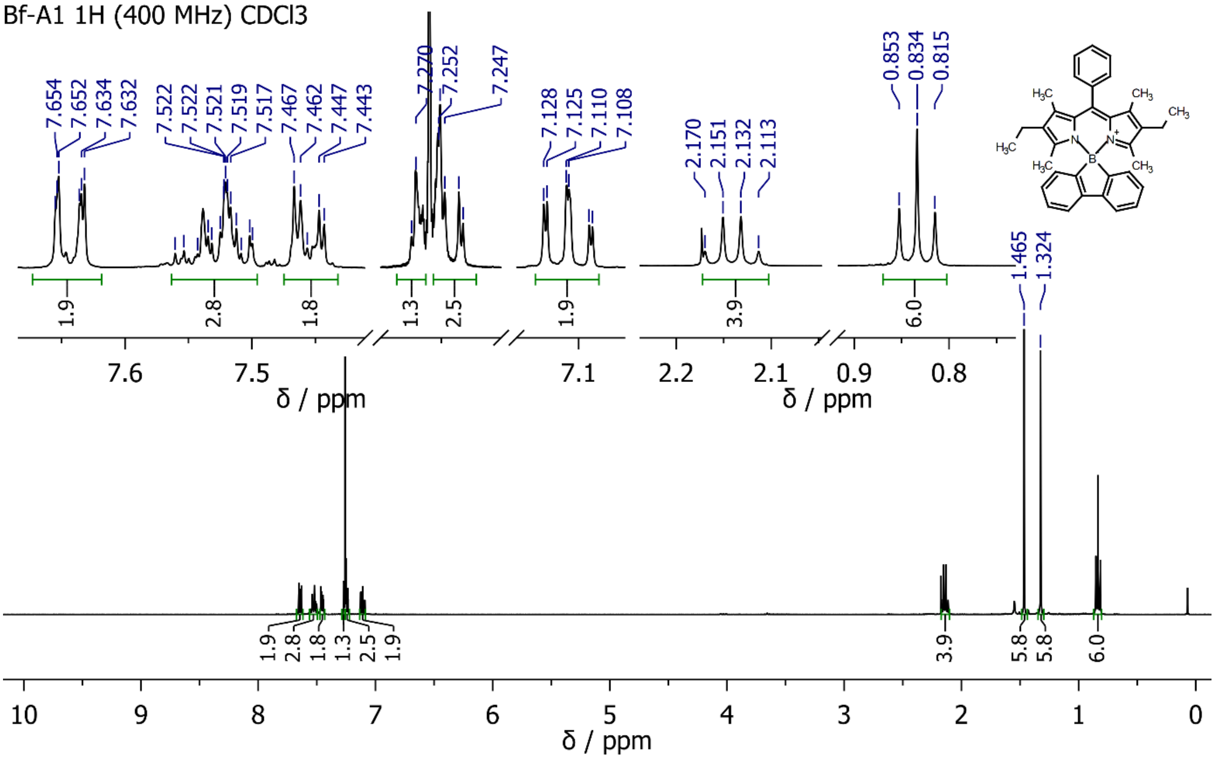

Figure S56. ^{1}H NMR (400 MHz) spectra of **Bf-A1** in $CDCl_3$.

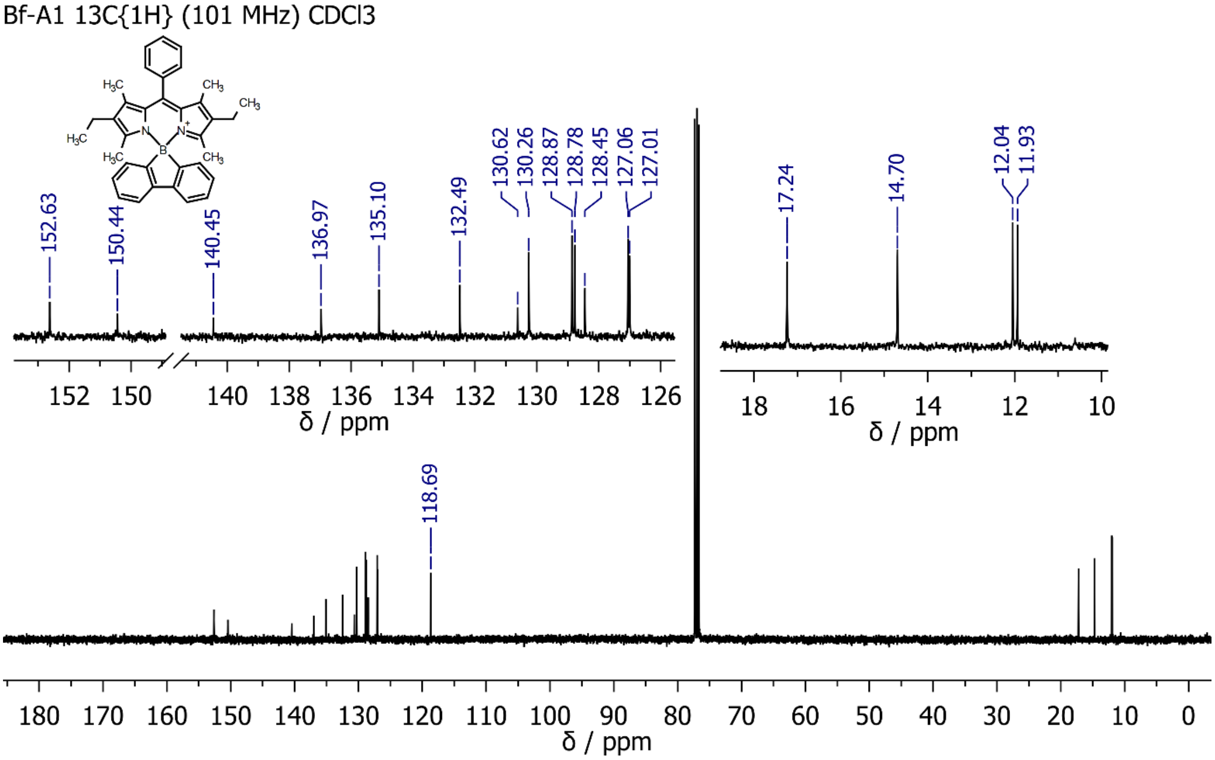

Figure S57. ^{13}C{1H} NMR (101 MHz) spectra of **Bf-A1** in $CDCl_3$.

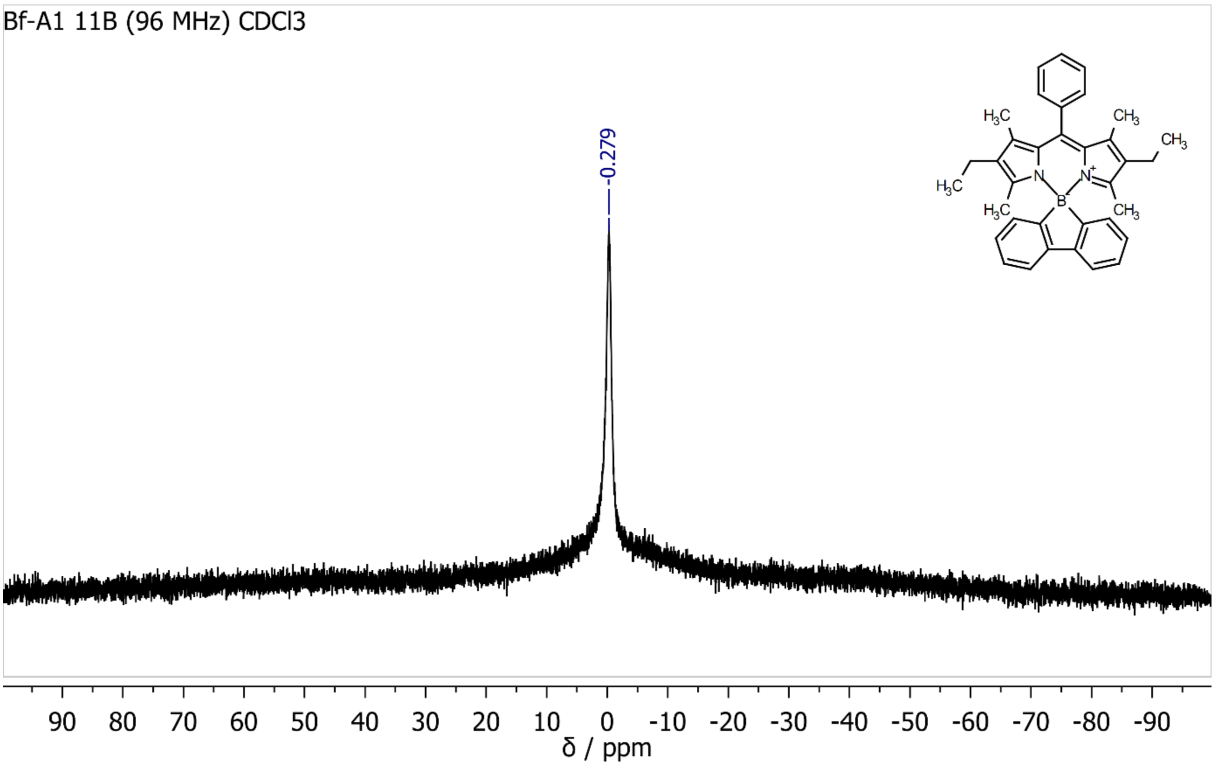

Figure S58. ^{11}B NMR (96 MHz) spectra of **Bf-A1** in $CDCl_3$.

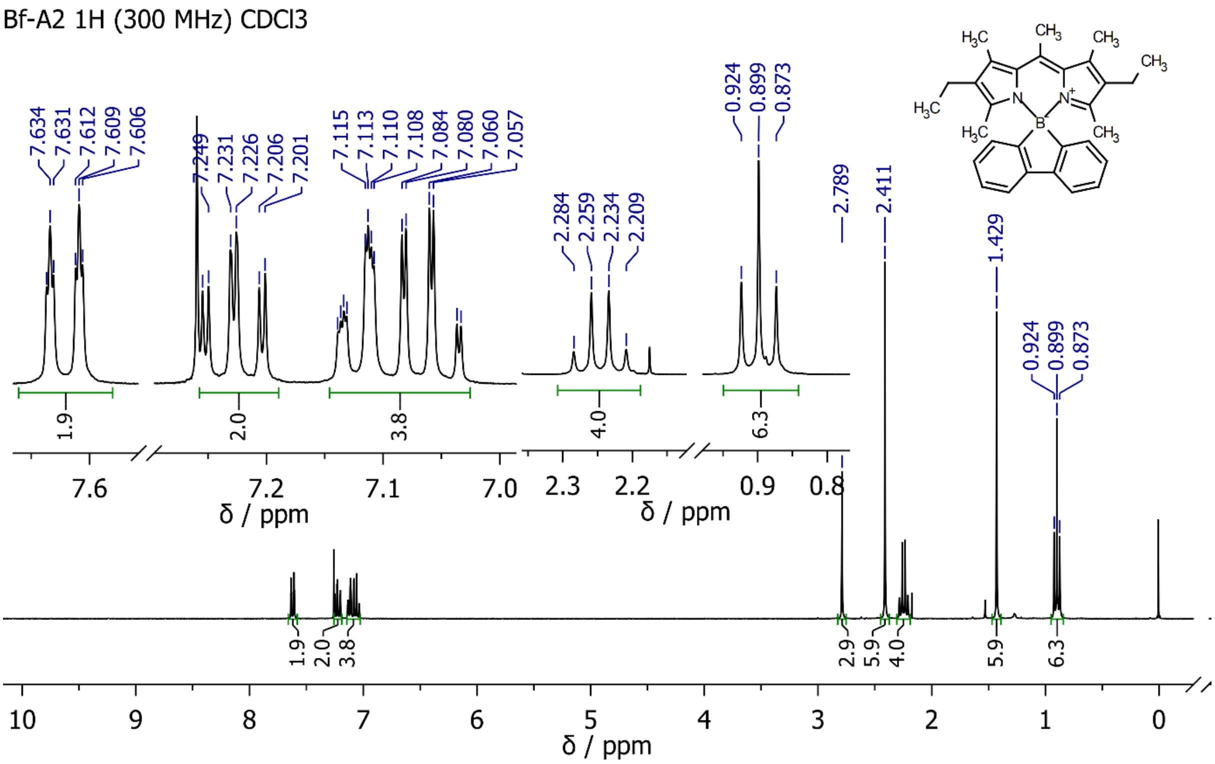

Figure S59. ^{1}H NMR (300 MHz) spectra of **Bf-A2** in $CDCl_3$.

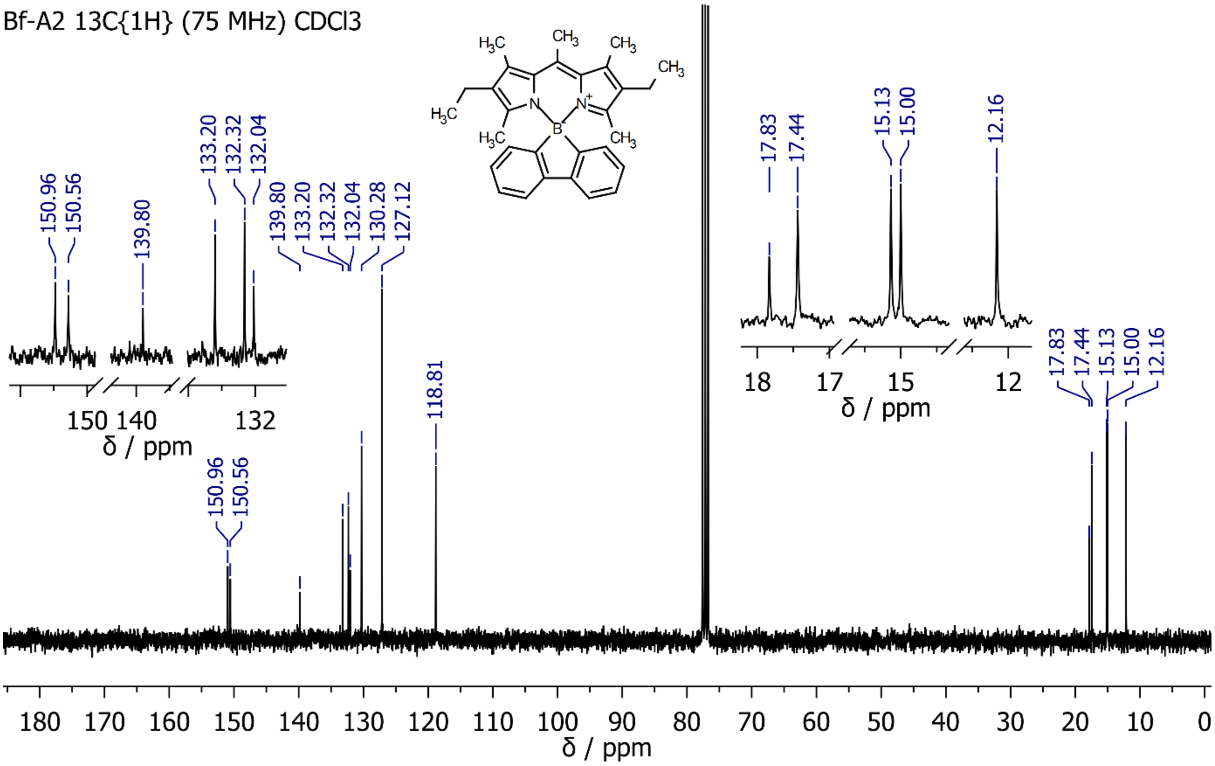

Figure S60. $^{13}C\{1H\}$ NMR (75 MHz) spectra of **Bf-A2** in $CDCl_3$.

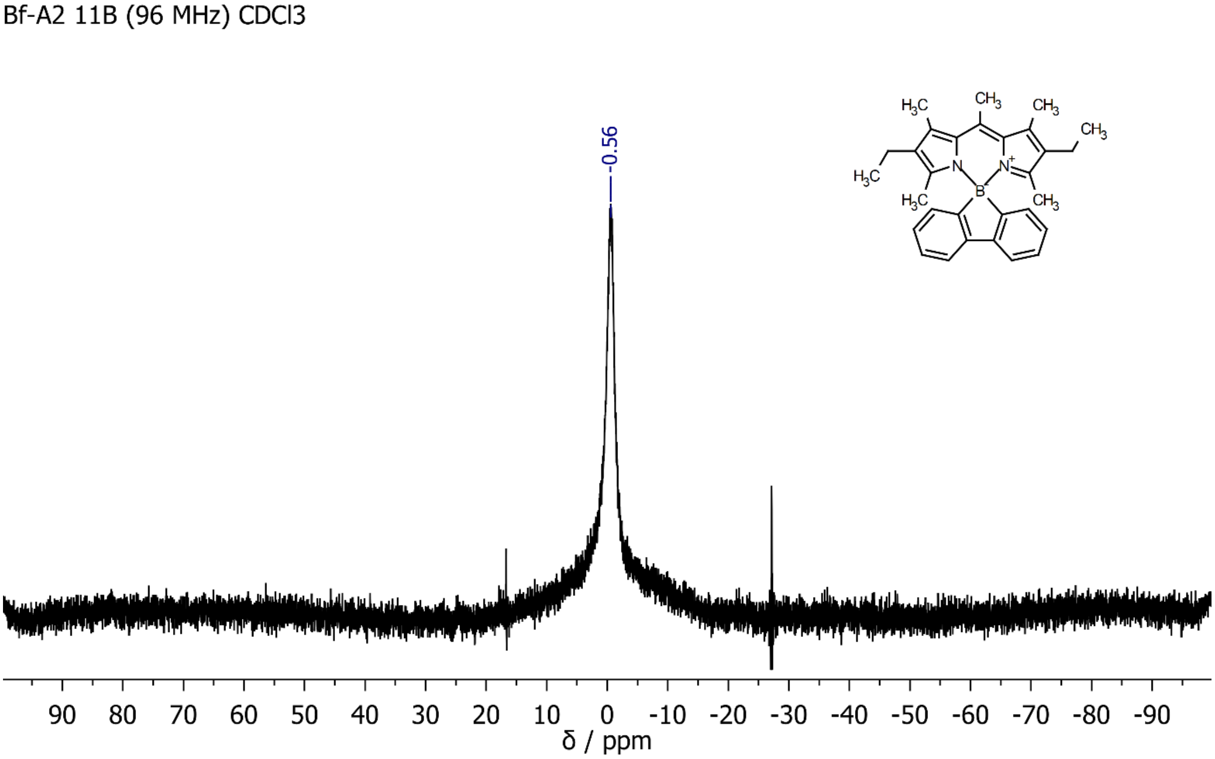

Figure S61. ^{11}B NMR (96 MHz) spectra of **Bf-A2** in $CDCl_3$.

1H NMR spectra of other BODIPY complexes and ligands

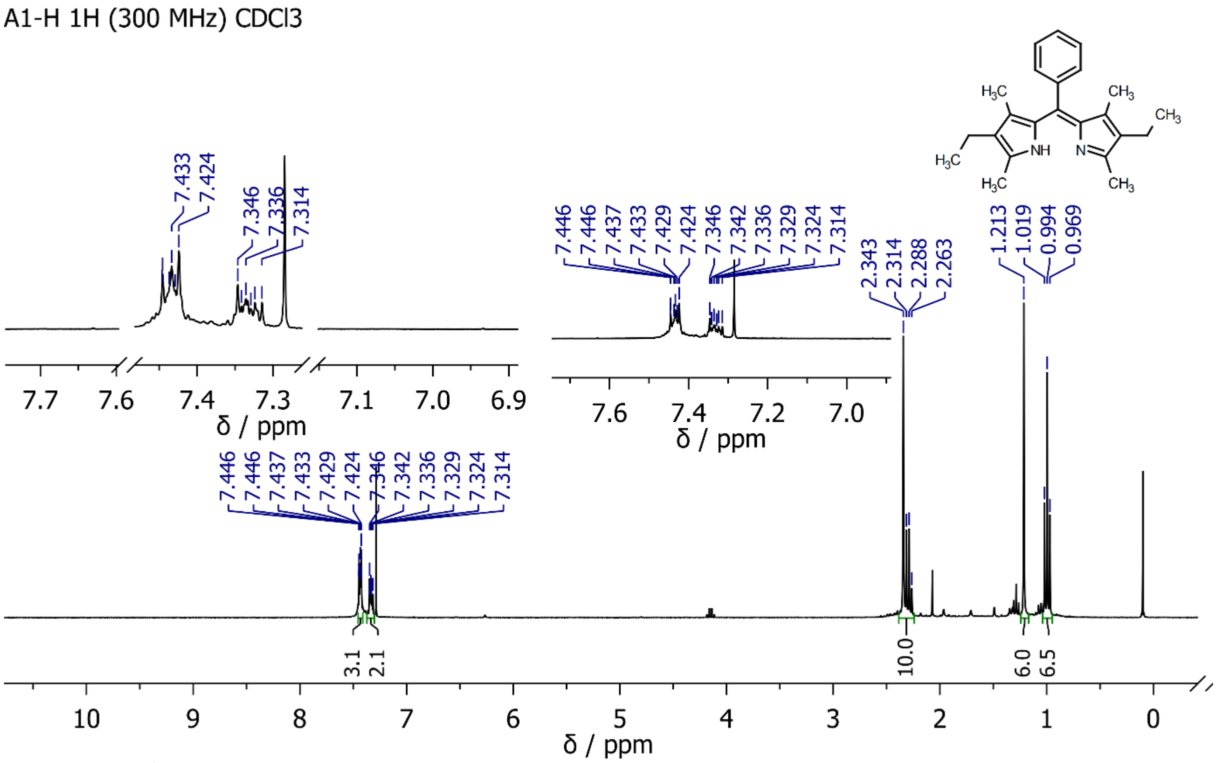

Figure S62. ^{1}H NMR (300 MHz) spectra of **A1-H** in $CDCl_3$.

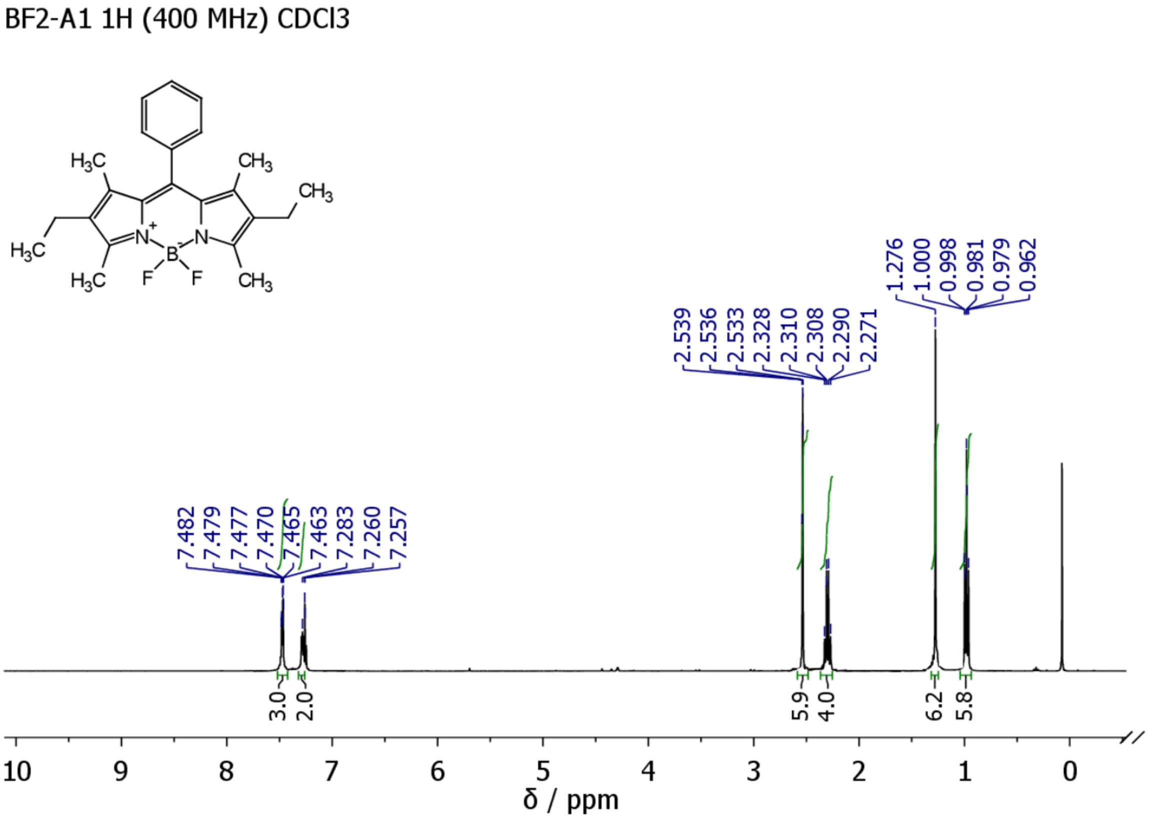

Figure S63. ^{1}H NMR (400 MHz) spectra of **BF_2-A1** in $CDCl_3$.

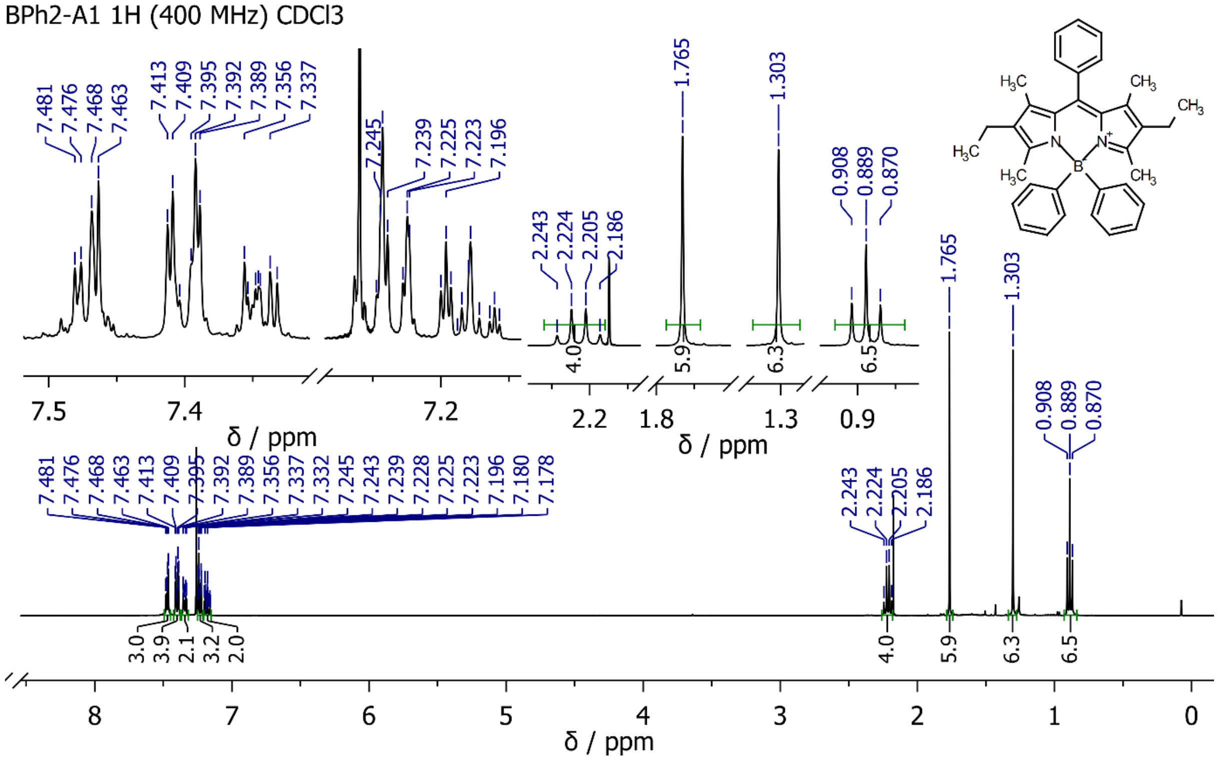

Figure S64. ^{1}H NMR (400 MHz) spectra of **BPh$_2$-A1** in $CDCl_3$.

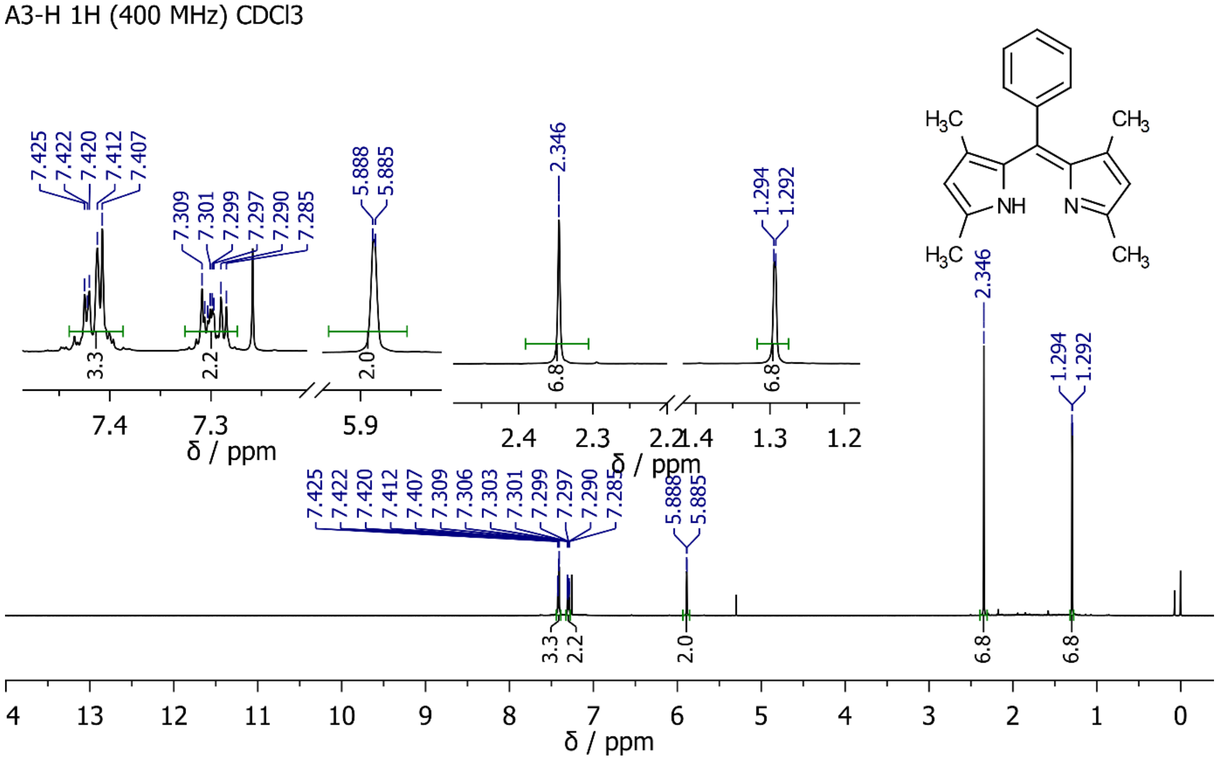

Figure S65. ^{1}H NMR (400 MHz) spectra of **A3-H** in $CDCl_3$.

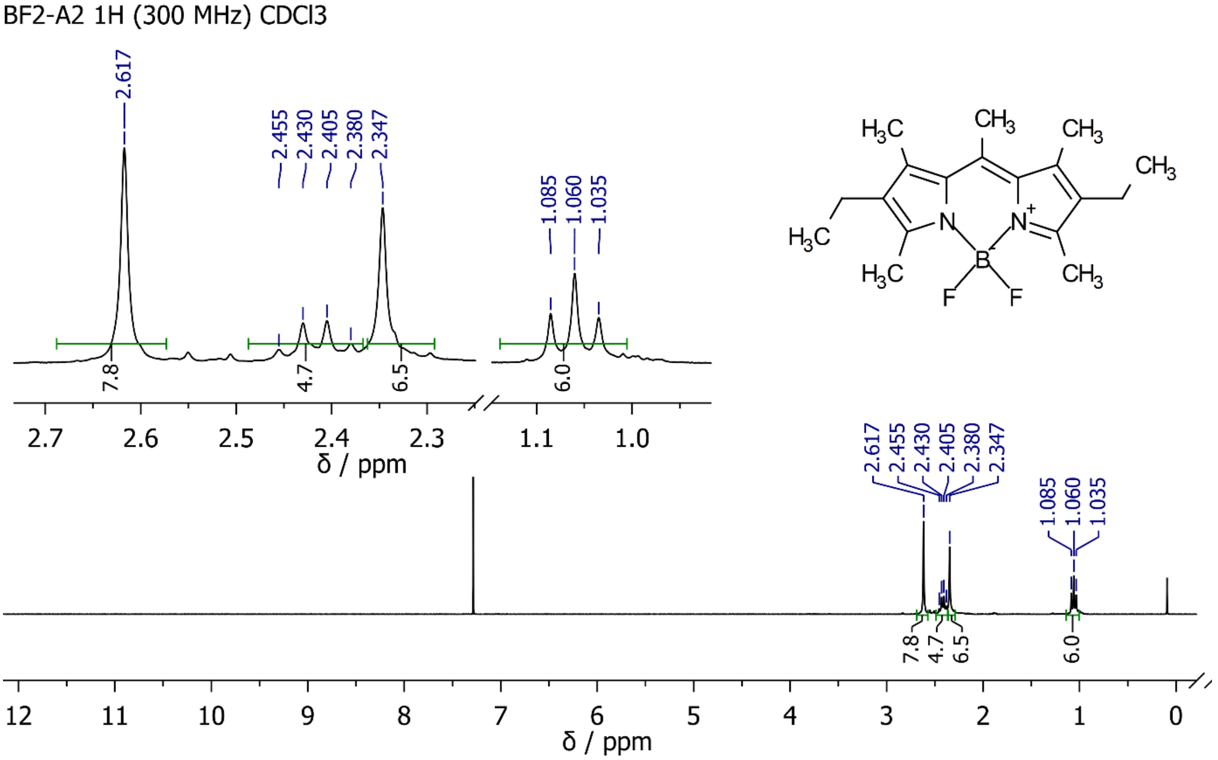

Figure S66. ^{1}H NMR (300 MHz) spectra of **BF_2-A2** in $CDCl_3$.

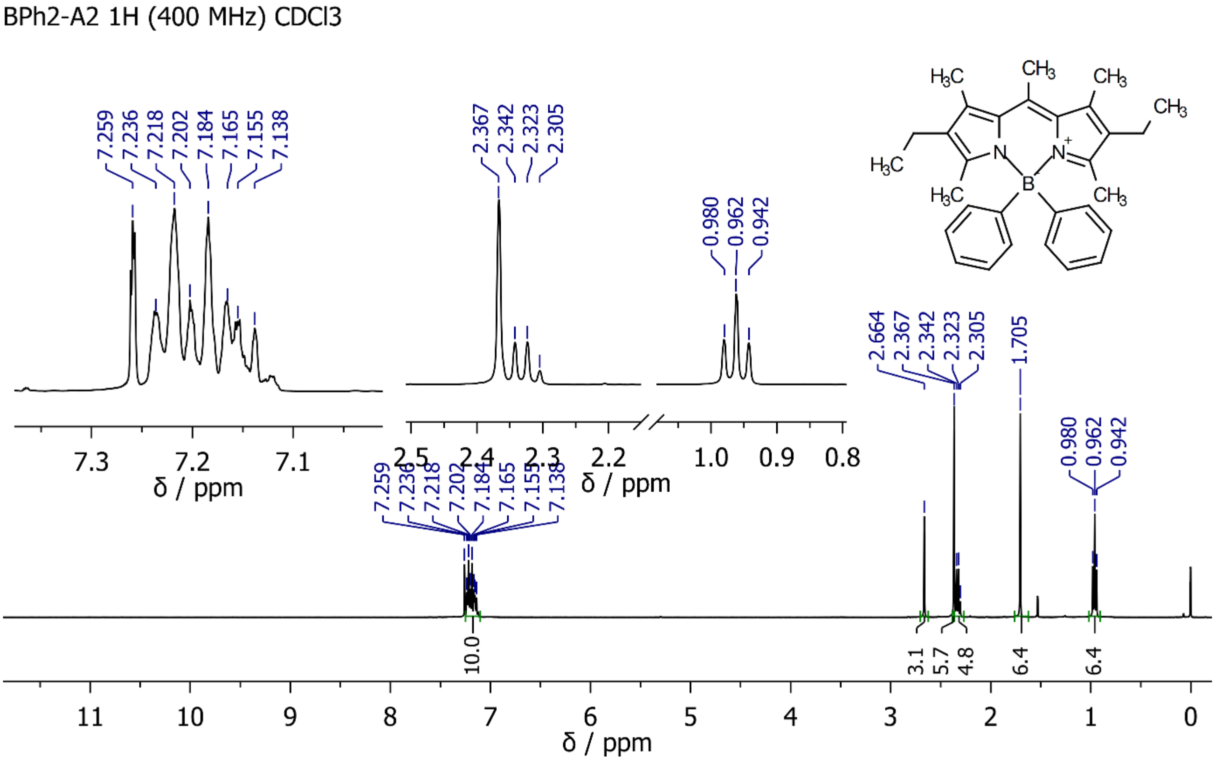

Figure S67. ^{1}H NMR (400 MHz) spectra of **BPh_2-A2** in $CDCl_3$.

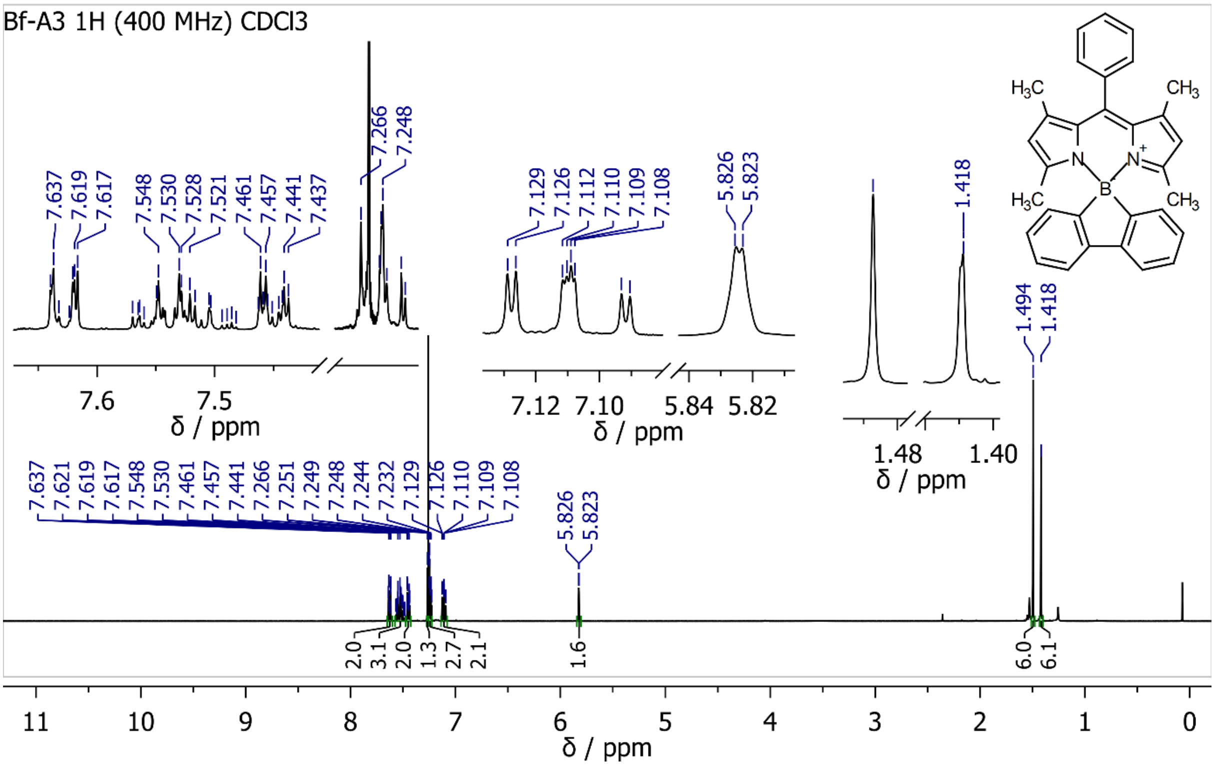

Figure S68. ^{1}H NMR (400 MHz) spectra of **Bf-A3** in $CDCl_3$.

9. References for Supporting Information.

(1) Würth, C.; Grabolle, M.; Pauli, J.; Spieles, M.; Resch-Genger, U. Relative and Absolute Determination of Fluorescence Quantum Yields of Transparent Samples. *Nat. Protoc.* **2013**, *8* (8), 1535–1550.

(2) Resch-Genger, U.; Rurack, K. Determination of the Photoluminescence Quantum Yield of Dilute Dye Solutions (IUPAC Technical Report). *Pure Appl Chem* **2013**, *85* (10), 22.

(3) Fischer, M.; Georges, J. Fluorescence Quantum Yield of Rhodamine 6G in Ethanol as a Function of Concentration Using Thermal Lens Spectrometry. *Chem. Phys. Lett.* **1996**, *260* (1), 115–118.

(4) Lakowicz, J. R. *Principles of Fluorescence Spectroscopy*; Springer science & business media, 2013.

(5) Rurack, K.; Spieles, M. Fluorescence Quantum Yields of a Series of Red and Near-Infrared Dyes Emitting at 600−1000 Nm. *Anal. Chem.* **2011**, *83* (4), 1232–1242.

(6) Velapoldi, R. A.; Tønnesen, H. H. Corrected Emission Spectra and Quantum Yields for a Series of Fluorescent Compounds in the Visible Spectral Region. *J. Fluoresc.* **2004**, *14* (4), 465–472.

(7) Aguiar, A.; Farinhas, J.; da Silva, W.; Susano, M.; Silva, M. R.; Alcácer, L.; Kumar, S.; Brett, C. M. A.; Morgado, J.; Sobral, A. J. F. N. Simple BODIPY Dyes as Suitable Electron-Donors for Organic Bulk Heterojunction Photovoltaic Cells. *Dyes Pigments* **2020**, *172*, 107842.

(8) Davies, L. H.; Harrington, R. W.; Clegg, W.; Higham, L. J. BR2BodPR2: Highly Fluorescent Alternatives to PPh3 and PhPCy2. *Dalton Trans.* **2014**, *43* (36), 13485–13499.

(9) López Arbeloa, F.; López Arbeloa, T.; López Arbeloa, I.; García-Moreno, I.; Costela, A.; Sastre, R.; Amat-Guerri, F. Photophysical and Lasing Properties of Pyrromethene567 Dye in Liquid Solution.: Environment Effects. *Chem. Phys.* **1998**, *236* (1), 331–341.

(10) Duran-Sampedro, G.; Esnal, I.; Agarrabeitia, A. R.; Bañuelos Prieto, J.; Cerdán, L.; García-Moreno, I.; Costela, A.; Lopez-Arbeloa, I.; Ortiz, M. J. First Highly Efficient and Photostable E and C Derivatives of 4,4-Difluoro-4-Bora-3a,4a-Diaza-s-Indacene (BODIPY) as Dye Lasers in the Liquid Phase, Thin Films, and Solid-State Rods. *Chem. – Eur. J.* **2014**, *20* (9), 2646–2653.

(11) Yang, L.; Ravi shekar, Y.; Hutton, S.; Lough, A.; Yan, H. Reactions of BODIPY Fluorophore with Cupric Nitrate. *Synlett* **2014**, *25*, 2661–2664.

(12) Urban, M.; Durka, K.; Górka, P.; Wiosna-Sałyga, G.; Nawara, K.; Jankowski, P.; Luliński, S. The Effect of Locking π-Conjugation in Organoboron Moieties in the Structures of Luminescent Tetracoordinate Boron Complexes. *Dalton Trans.* **2019**, *48* (24), 8642–8663.

(13) Baciocchi, E.; Giacco, T. D.; Elisei, F.; Gerini, M. F.; Guerra, M.; Lapi, A.; Liberali, P. Electron Transfer and Singlet Oxygen Mechanisms in the Photooxygenation of Dibutyl Sulfide and Thioanisole in MeCN Sensitized by N-Methylquinolinium Tetrafluoborate and 9,10-Dicyanoanthracene. The Probable Involvement of a Thiadioxirane Intermediate in Electron Transfer Photooxygenations. *J. Am. Chem. Soc.* **2003**, *125* (52), 16444–16454.

(14) Bonesi, S. M.; Fagnoni, M.; Albini, A. Photosensitized Electron Transfer Oxidation of Sulfides: A Steady-State Study. *Eur. J. Org. Chem.* **2008**, *2008* (15), 2612–2620.

(15) Bonesi, S. M.; Manet, I.; Freccero, M.; Fagnoni, M.; Albini, A. Photosensitized Oxidation of Sulfides: Discriminating between the Singlet-Oxygen Mechanism and

Electron Transfer Involving Superoxide Anion or Molecular Oxygen. *Chem. – Eur. J.* **2006**, *12* (18), 4844–4857.
(16) CrysAlis, P. Agilent Technologies Ltd. *Yarnton Oxfs. Engl.* **2014**.
(17) Sheldrick, G. M. SHELXT - Integrated Space-Group and Crystal-Structure Determination. *Acta Crystallogr. Sect. Found. Adv.* **2015**, *71* (Pt 1), 3–8.
(18) Sheldrick, G. M. Crystal Structure Refinement with SHELXL. *Acta Crystallogr. Sect. C Struct. Chem.* **2015**, *71* (Pt 1), 3–8.
(19) Dolomanov, O. V.; Bourhis, L. J.; Gildea, R. J.; Howard, J. a. K.; Puschmann, H. OLEX2: A Complete Structure Solution, Refinement and Analysis Program. *J. Appl. Crystallogr.* **2009**, *42* (2), 339–341.
(20) Kubota, Y.; Uehara, J.; Funabiki, K.; Ebihara, M.; Matsui, M. Strategy for the Increasing the Solid-State Fluorescence Intensi-ty of Pyrromethene–BF2 Complexes. *Tetrahedron Lett.* **2010**, *51* (47), 6195–6198.
(21) Yuan, K.; Wang, X.; Mellerup, S. K.; Kozin, I.; Wang, S. Spiro-BODIPYs with a Diaryl Chelate: Impact on Aggregation and Luminescence. *J. Org. Chem.* **2017**, *82* (24), 13481–13487.
(22) Frisch, M. J.; Trucks, G. W.; Schlegel, H. B.; Scuseria, G. E.; Robb, M. A.; Cheeseman, J. R.; Scalmani, G.; Barone, V.; Petersson, G. A.; Nakatsuji, H.; Li, X.; Caricato, M.; Marenich, A. V.; Bloino, J.; Janesko, B. G.; Gomperts, R.; Mennucci, B.; Hratchian, H. P.; Ortiz, J. V.; Izmaylov, A. F.; Sonnenberg, J. L.; Williams; Ding, F.; Lipparini, F.; Egidi, F.; Goings, J.; Peng, B.; Petrone, A.; Henderson, T.; Ranasinghe, D.; Zakrzewski, V. G.; Gao, J.; Rega, N.; Zheng, G.; Liang, W.; Hada, M.; Ehara, M.; Toyota, K.; Fukuda, R.; Hasegawa, J.; Ishida, M.; Nakajima, T.; Honda, Y.; Kitao, O.; Nakai, H.; Vreven, T.; Throssell, K.; Montgomery Jr., J. A.; Peralta, J. E.; Ogliaro, F.; Bearpark, M. J.; Heyd, J. J.; Brothers, E. N.; Kudin, K. N.; Staroverov, V. N.; Keith, T. A.; Kobayashi, R.; Normand, J.; Raghavachari, K.; Rendell, A. P.; Burant, J. C.; Iyengar, S. S.; Tomasi, J.; Cossi, M.; Millam, J. M.; Klene, M.; Adamo, C.; Cammi, R.; Ochterski, J. W.; Martin, R. L.; Morokuma, K.; Farkas, O.; Foresman, J. B.; Fox, D. J. *Gaussian 16 Rev. C.01*; Wallingford, CT, 2016.
(23) Becke, A. D. Density-functional Thermochemistry. III. The Role of Exact Exchange. *J. Chem. Phys.* **1993**, *98* (7), 5648–5652.
(24) Lee, C.; Yang, W.; Parr, R. G. Development of the Colle-Salvetti Correlation-Energy Formula into a Functional of the Electron Density. *Phys. Rev. B* **1988**, *37* (2), 785–789.
(25) Krishnan, R.; Binkley, J. S.; Seeger, R.; Pople, J. A. Self-consistent Molecular Orbital Methods. XX. A Basis Set for Correlated Wave Functions. *J. Chem. Phys.* **1980**, *72* (1), 650–654.
(26) Hanwell, M. D.; Curtis, D. E.; Lonie, D. C.; Vandermeersch, T.; Zurek, E.; Hutchison, G. R. Avogadro: An Advanced Semantic Chemical Editor, Visualization, and Analysis Platform. *J. Cheminformatics* **2012**, *4*, 17.
(27) Tomasi, J.; Mennucci, B.; Cammi, R. Quantum Mechanical Continuum Solvation Models. *Chem. Rev.* **2005**, *105* (8), 2999–3094.
(28) Martin, R. L. Natural Transition Orbitals. *J. Chem. Phys.* **2003**, *118* (11), 4775–4777.
